# Evaluating the Utilities of Foundation Models in Single‐Cell Data Analysis

**DOI:** 10.1002/advs.202514490

**Published:** 2026-03-23

**Authors:** Tianyu Liu, Kexing Li, Yuge Wang, Hongyu Li, Hongyu Zhao

**Affiliations:** ^1^ Interdepartmental Program in Computational Biology & Bioinformatics Yale University Connecticut USA; ^2^ Department of Biostatistics Yale University Connecticut USA

**Keywords:** benchmark, deep learning, foundation model, large language model, single‐cell data

## Abstract

Foundation Models (FMs) have made significant strides in both industrial and scientific domains. In this paper, we evaluate the performance of FMs for single‐cell sequencing data analysis through comprehensive experiments across eight downstream tasks pertinent to single‐cell data. Overall, the top FMs include scGPT, Geneformer, and CellFM by considering model performances and user accessibility among ten single‐cell FMs. However, by comparing these FMs with task‐specific methods, we found that single‐cell FMs may not consistently excel than task‐specific methods in all tasks, which challenges the necessity of developing foundation models for single‐cell analysis. In addition, we evaluated the effects of hyperparameters, initial settings, and stability for training single‐cell FMs based on a proposed **scEval** framework, and provide guidelines for pre‐training and fine‐tuning to enhance the performances of single‐cell FMs. Our work summarizes the current state of single‐cell FMs, points to their constraints and avenues for future development, and offers a freely available evaluation pipeline to benchmark new models and improve method development.

## Graphical abstract

This study delivers the first systematic, task‐level evaluation of single‐cell foundation models across eight core analytical tasks. By benchmarking 10 leading models with the scEval framework, it reveals where foundation models truly add value, where task‐specific methods still dominate, and provides concrete, reproducible guidelines to steer the next generation of reliable single‐cell Foundation Models.

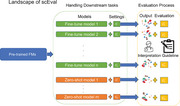

## 1 Introduction

Single‐cell sequencing technologies offer high‐throughout observations into complex biological systems at the cell level with multimodal data [1, 2]. They help elucidate disease mechanisms and potential treatments [3, 4, 5]. In line with the central dogma of molecular biology, these technologies enable the characterization of various molecules, such as DNA (e.g., scDNA‐seq) [6], RNA (e.g., scRNA‐seq) [7, 8], and proteins (e.g., Cite‐seq) [9]. Furthermore, single‐cell sequencing can facilitate epigenetic studies, including chromatin accessibility (e.g., scATAC‐seq) [10, 11] and methylation [12, 13]. These technologies have been rated as among the most impactful ones in recent years [14, 15].

With the development of single‐cell technologies, expansive single‐cell datasets have been collected, and they present challenges in integration, interpretation, and downstream utilization. Therefore, there is a need to build a Foundation Model (FM), which can benefit from prior knowledge for single‐cell data analysis. In a similar motivation, natural language processing (NLP) also boasts extensive datasets, where FMs such as pre‐trained Large Language Models (LLMs) have shown great success in addressing NLP tasks or multimodal tasks [16]. Numerous LLMs, including GPT‐4 [17] and LLaMA [18], excel at diverse language‐related tasks such as question answering and sentence generation, which has received widespread attention from both the AI community and society [19]. Moreover, these LLMs showcase impressive performance in zero‐shot learning, thereby enabling them to address tasks beyond their original training scope, such as solving mathematical problems [20]. Similarly, a good FM in single‐cell data analysis should also handle multiple tasks with a unified framework.

Indeed, notable parallels exist between studies based on single‐cell sequencing data and those in NLP. Both fields leverage advanced technologies, such as transformer architectures, which have proven effective in handling a variety of tasks within each domain [21, 22, 23, 24]. Similarly, both disciplines emphasize the importance of analyzing intra‐data and inter‐data relationships, which in the context of single‐cell analysis might involve interactions among genes or cells [23, 25, 26, 27]. Moreover, the concept of representation learning is central to both fields: effective embeddings of cells and genes can be developed using techniques analogous to those used for generating token embeddings in NLP [28]. Lastly, the success of both fields hinges on access to high‐quality databases [16, 29], underscoring the need for careful data selection during model training. These synergies suggest that integrating methodologies from NLP may significantly advance the analysis and interpretation of single‐cell data.

While FMs have seen marked success in the realms of DNA analysis [30] and biomedical NLP [31, 32], their application in single‐cell research remains largely uncharted. There is a limited number of robust pre‐trained models (known as single‐cell FMs) capable of managing multiple tasks in single‐cell research. Some single‐cell FMs focus on cell‐type annotation or gene function prediction, including scBERT [33], CellLM [34], and Geneformer [35], while others aim to create an FM in this area that can handle multiple tasks, including scGPT [36], scFoundation [37], tGPT [38], GeneCompass [39], SCimilarity [40], UCE [41], CellFM [42], and CellPLM [43]. Details of these models can be found in Appendices E and F. Furthermore, no studies to date have comprehensively evaluated the utility of these models and provided guidance for model training. Little has been done to compare NLP‐focused LLMs with those used for single‐cell research to gain insight into scaling laws and zero‐shot (or few‐shots) learning abilities of the latter.

**FIGURE 1:**
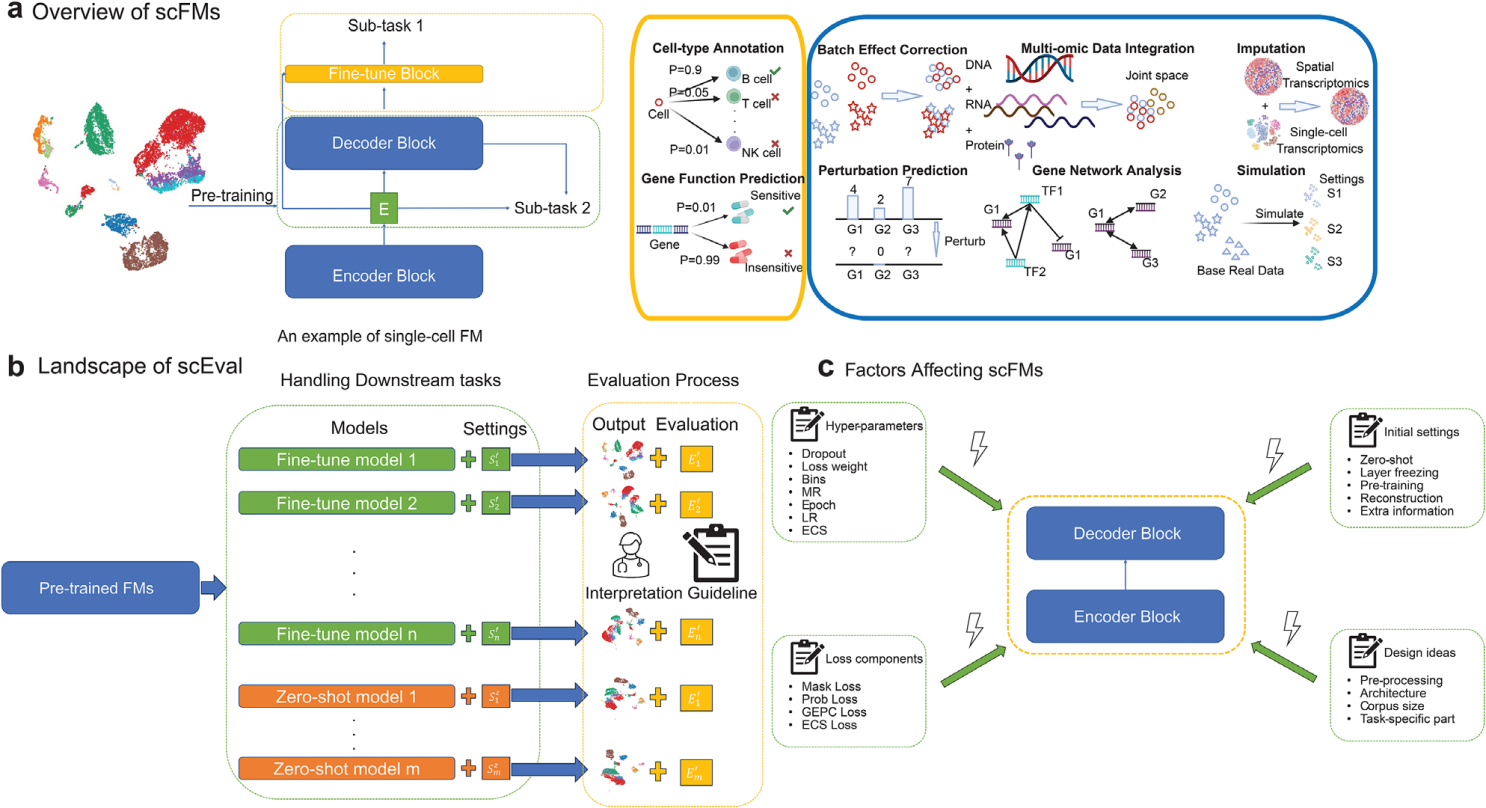
Overview of single‐cell FMs, landscape of scEval and factors affecting single‐cell FMs. (a): Overview of single‐cell FMs describing the typical structure of FMs and general tasks for single‐cell data analysis. The right two blocks represent two types of downstream tasks. Yellow block: Sub‐task 1, including Cell‐type Annotation and Gene Function Prediction (top to bottom). Blue block: Sub‐task 2, including Batch Effect Correction, Multi‐omic Data Integration, Imputation (From left to right, top row), Perturbation Prediction, Gene Network Analysis, and Simulation (From left to right, bottom row). (b): The landscape of scEval shows the workflow of our systematic evaluation. Here, we consider models based on fine‐tuning design with settings $S_{1}^{f}$ to $S_{n}^{f}$, and they have corresponding evaluation pipelines $E_{1}^{f}$ to $E_{n}^{f}$. We also consider models based on zero‐shot‐learning design with settings $S_{1}^{z}$ to $S_{m}^{z}$, and they have corresponding evaluation pipelines $E_{1}^{z}$ to $E_{m}^{z}$. The reason for using the above notations to represent the process of evaluation is that different models can perform different tasks, while different tasks have different evaluation scenarios. (c): Factors, which can affect the performance of single‐cell FMs. These factors are summarized based on different designs of single‐cell FMs. The known factors can be classified into four different types. Details of hyper‐parameters can be found in Appendix A. Details of initial settings can be found in Appendix B.

Here, we investigate the overlap of the pre‐training stages of different single‐cell FMs, and present a framework for assessing various single‐cell FMs and tasks (shown in Figure 1a), termed as Single‐cell Large Language Model Evaluation (**scEval**), shown in Figure 1b. Using scEval, we not only compare different single‐cell FMs across various datasets and tasks as a horizontal comparison but also identify critical parameters and strategies for the fine‐tuning process of specific models as a vertical comparison. We also examine the potential contributions of model scaling of single‐cell FMs, substantiating that the latter also possesses distinctive abilities. To help the audience better understand FMs, we provide a glossary summary of common terms in Artificial Intelligence (AI) and Machine Learning (ML) in Supporting information file 1.

## 2 Results

### 2.1 Overview of Our Evaluations

We evaluated the performance and user accessibility of eleven open‐source single‐cell FMs (scGPT, Geneformer, scBERT, CellLM, tGPT, SCimilarity, CellPLM, UCE, scFoundation, and CellFM, and GeneCompass) by assessing their outputs on 8 tasks with 29 datasets with both zero‐shot setting and possible fine‐tuning setting. We did not evaluate all the models with all the datasets with the reasons provided in Supporting Information files 2 and 3, but tried our best to implement the missing functions for evaluation. The tasks that can be performed for different models, the overall ranks, as well as user accessibility are summarized in Tables 1 a–c. We also compared their performance with state‐of‐the‐art (SOTA) task‐specific methods. Our workflow can be summarized in Extend Data Figure 1. The top three methods include scGPT, Geneformer, and CellFM by considering both usability and performance. For each task, we evaluate single‐cell FMs based on their default settings for a fair comparison. Moreover, we discuss the effect of different parameter settings on model performance and investigate the contribution of different loss functions of scGPT and initial settings by ablation tests. We also consider the contribution of model scales to the performance of FMs. Finally, we evaluate the stability and usability of different single‐cell FMs and make recommendations for preferred models. The detailed design of scEval is explained in the Methods section. To ensure fairness, we selected metrics for different tasks based on their benchmarking analysis, and we fixed all models to be from versions before December 1, 2025.

**TABLE 1:** Overall comparisons of model capacity and performance. (a) Evaluations for the capacity of different single‐cell FMs. We utilized different signs to denote the default settings we tested in scEval. Details are explained in the table legend. The icons are inspired by [44]. (b) Evaluations for both performance and usability of different single‐cell FMs. We record the ranks of different methods across different tasks and user accessibility evaluations and compute the final overall rank. A lower rank means a better model. The top three methods are boldfaced. (c) User accessibility evaluation of different single‐cell FMs. A smile face represents the selected single‐cell FM can process the corresponding task by default.

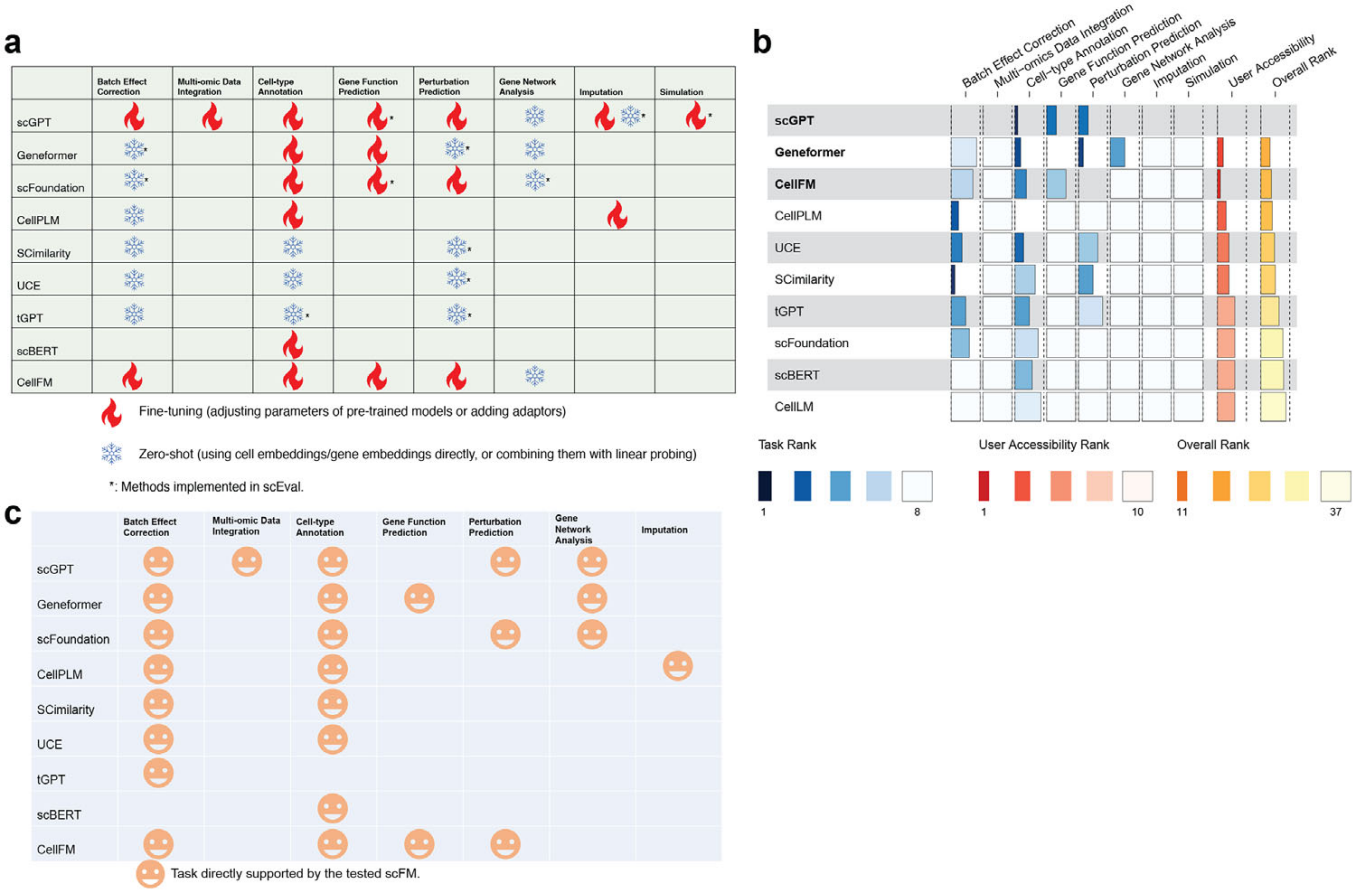

### 2.2 Comparisons of Pre‐Training Datasets for Different Single‐Cell FMs

In this section, we investigated and compared the pre‐training steps of different single‐cell FMs from three dimensions, including the scale of pre‐training datasets, the diversity of pre‐training datasets, and the major human tissues or organs overlap of pre‐training datasets. Based on Figure S2, we found that GeneCompass had the largest pre‐training datasets, followed by scFoundation. However, GeneCompass has running issues, so we could not fully reproduce their results. Therefore, we emphasize that the foundation for evaluating an scFM is ensuring its operational viability; therefore, we do not consider GeneCompass as the primary subject of discussion in this manuscript. Interestingly, the pre‐training scales of UCE, scGPT, Geneformer, SCimilarity, and tGPT are comparable, which implies that having 20–30 million may be acceptable for constructing pre‐training datasets. Moreover, SCimilarity had the largest diversity in the datasets conditioned on diseases compared with other methods. Most of the single‐cell FMs chose to include cancer cells in their pre‐training process, which was possibly due to many single‐cell studies focused on cancer [45]. UCE had the largest diversity in the datasets from different species, while the rest of the single‐cell FMs focused more on human. In addition, few models considered the use of multi‐omic data information, except tGPT, CellPLM, and UCE. However, in the pre‐training process, UCE and tGPT omitted the extra information (for example, the spatial location of spatial transcriptomic data) provided by these datasets and treated the data the same as scRNA‐seq data. Using different pre‐training pipelines for multi‐omic datasets might improve the performance of current models on addressing related tasks. In Figure S3, we computed the overlap of major human tissues or organs used for pre‐training across different models. Geneformer had the largest diversity in this comparison because we treat it as a reference method. UCE had the largest overlap compared with Geneformer. scBERT and CellLM shared major human tissues or organs because they both used PanglaoDB [46] for pre‐training. Interestingly, scGPT did not use many types of tissues for pre‐training. Considering its relatively better performance, including as many types of datasets as possible may not necessarily lead to better performance in our evaluation for selected tasks. Therefore, careful task‐specific data collection and data ablation analysis is needed for the pre‐training stage of new models.

Furthermore, we analyzed the relationship between these statistics and model performance. Here, we considered two major tasks, batch effect correction, and cell‐type annotation, for our comparison. We compared four FMs for batch effect correction and seven FMs for cell‐type annotation, thus offering statistical meaning to perform further investigation. For each task, we computed the Spearman correlation under three settings, including performance versus pre‐training data scale, performance versus the number of major tissues for pre‐training, and performance versus the number of model parameters. However, all the six correlations computed based on the comparisons did not show statistical significance (*p*‐value > 0.05). Therefore, the relationship between the performance of single‐cell FMs on downstream tasks and their pre‐training settings might be affected by many factors, including pre‐training strategies, data‐cleaning pipelines, rules of training‐testing datasets splitting, and others. Therefore, we focus on task‐driven analysis and offer guidelines for model development based on their performances on different tasks.

Finally, we include a table that represents whether our evaluation datasets are used for the pre‐training of single‐cell FMs with known pre‐training information in Supporting Information file 2. This table shows that most of the datasets were not included by all single‐cell FMs, so we had a sparse table.

### 2.3 Evaluation Based on Cell‐Perspective Tasks Shows That Single‐Cell FMs can Reduce Batch Effect and Annotate Cell Types

#### 2.3.1 Batch Effect Correction

For this task, we intend to reduce batch effect of scRNA‐seq datasets. We considered scGPT, tGPT, UCE, SCimilarity, CellPLM, Geneformer, scFoundation, CellFM, Harmony [48], and ResPAN [49] for this task. We also provided a detailed analysis of the influence of various hyperparameters on the performance of scGPT on batch effect correction. The description of the evaluation metric $S_{final}$ can be found in Appendix D. We computed $S_{final}$ based on the weighted average of metrics for evaluating the level of batch effect removal and the metrics for evaluating the level of biological variance conservation.

We first compared the performances of different single‐cell FMs based on the zero‐shot setting for this task, shown in Figure S4. According to this figure, task‐specific methods such as Harmony and ResPAN surpass the rest of the single‐cell FMs, implying that the ability of FMs to remove the batch effect is limited under the zero‐shot setting. Moreover, since scGPT also supports fine‐tuning mode for correcting batch effect, we also compared the zero‐shot setting and fine‐tuning setting of scGPT for batch effect correction. According to Figure S5a the fine‐tuning mode has a better overall score compared with the zero‐shot mode, and thus fine‐tuning scGPT can help in reducing batch effects in scRNA‐seq data analysis.

**FIGURE 2:**
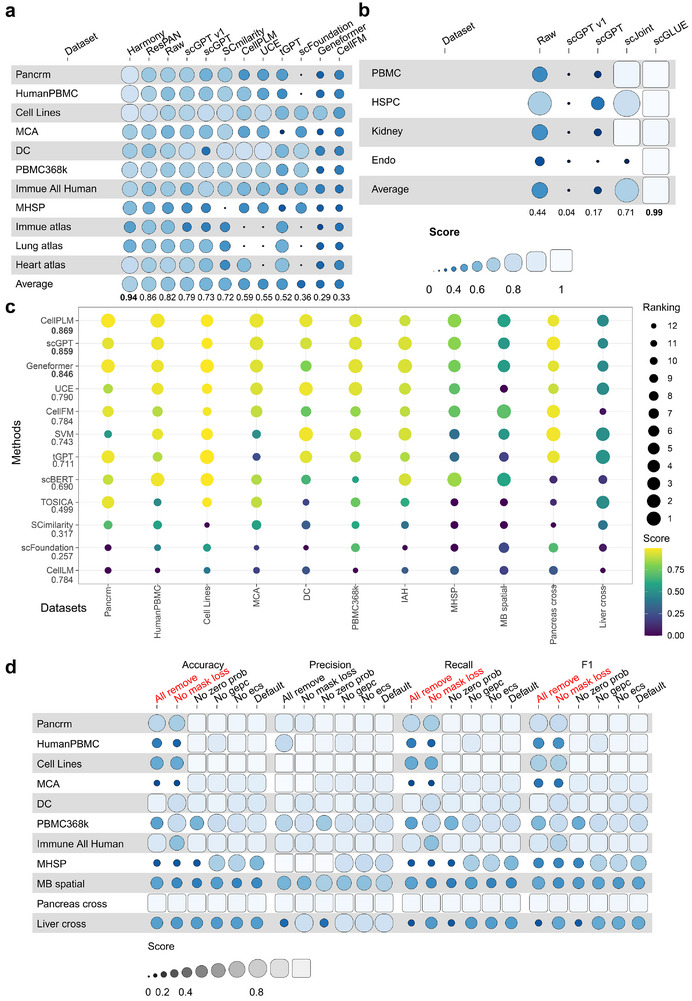
Experimental results of single‐cell FMs and benchmarking methods for cell‐level tasks. (a) An overall assessment of raw data (The “Raw” label in this figure) and data after batch effect correction based on different methods. scGPT v1 represents the scGPT model with smaller pre‐training datasets compared to scGPT. (b) An overall assessment of raw data and data after multi‐omic data integration based on different methods. (c) Comparison among models in the cell‐type annotation task. The scores on the left represent the average accuracy of different models across different datasets. (d) Ablation tests of the loss function components for cell‐type annotation. The red components are significant (one‐sided Wilcoxon Rank‐sum Test, p‐value = 0.03, *n* = 11). The panels (a) and (b) follow the default settings of funkyheatmap [47] for visualization.

We further compared different single‐cell FMs based on their default setting. As shown in Figure 2a, scGPT outperformed ResPAN in one of the 11 datasets and outperformed Harmony in 2 of the 11 datasets, while Harmony had an overall best performance. In the comparison of FMs, scGPT v1 had the best performance, and scGPT v1 outperformed the scGPT model on average, raising the issue of the need for increasing the size of pre‐training datasets for this task. Moreover, only Harmony and ResPAN achieved better $S_{final}$ compared with the raw datasets on average. Therefore, the ability of FMs to remove the batch effect is limited. Moreover, these FMs had worse performance in reducing the batch effect for large‐scale datasets. One reason is that their biology conservation scores were lower than those of raw datasets (shown in Figure S6). UCE and scFoundation could not handle large‐scale datasets due to running errors, which also raised problems in model accessibility. Figures S8 and S9 show the Uniform Manifold Approximation and Projection (UMAP) [50] plots for the raw data and the scGPT results. We could still observe the batch effect in the output of scGPT for Pancrm, HumanPBMC, MCA, MHSP, DC, Lung atlas, Immune atlas, and Heart atlas datasets. We also evaluated the performances of single‐cell FMs and task‐specific methods for preserving the trajectory information during the batch effect correction process, shown in Figure S7, and the metric used here is trajectory score. Based on this figure, Harmony still had the best performance, followed by scGPT.

We provide a detailed analysis of the impact of various hyper‐parameters on the performance of scGPT in batch effect correction based on Figure S10. A smaller learning rate tended to lead to better performance across all datasets. The optimal number of training epochs varied across datasets, with a larger number of epochs being beneficial for most datasets. This result contradicts recent research advocating for a single‐epoch training approach [51], suggesting that the optimal number of epochs might be context‐dependent. Increasing the number of bins is generally associated with an increase in the final score. The impact of the mask ratio and dropout rate on model performance is unclear, suggesting that further investigation is needed to understand their influence. These observations may improve the application of scGPT for batch effect correction in single‐cell data analysis and may also inform fine‐tuning of other similar models.

Figure S11a presents the comparison of scores across different initial settings for the batch effect correction task using scGPT. We can see that scGPT is capable of performing zero‐shot learning in batch effect correction. For the Cell Lines dataset, the zero‐shot learning approach even achieved the highest score, indicating that it may be an effective method for certain datasets. Moreover, pre‐training significantly contributes to the performance of scGPT in the batch effect correction task. Without pre‐training, the model's performance notably decreased. Using cross‐entropy as the loss function for gene expression reconstruction yielded better results than the mean square error (MSE) loss for most datasets. Freezing weights is not crucial for batch effect correction. Interestingly, the encoder structure appears to play a more important role in the training process, as freezing the encoder layer led to a larger decrease in the score. Incorporating the cell type information as a human label into the training process enhanced performance for most datasets. These observations suggest that each dataset may require unique tuning, underscoring the importance of adaptable methodologies in single‐cell RNA‐seq data analysis.

Figure S11b shows the performance metrics versus the choices of different optimizers for the batch effect correction. Adam and AdamW [52] had comparable performance in this task, while SGD [53], Sophia‐G [54] (a novel optimizer that is designed for training FMs) and Lion [55] (an optimized version of Adam) were worse. Therefore, the optimizers in the Adam family are preferred.

Figure S11c illustrates the impact of different loss function components on the performance of batch effect correction using scGPT. Here, we consider Mask Loss (It means we mask the gene expression levels of some genes and reconstruct them to compute the loss), Prob Loss (i.e., we predict whether some genes are expressed in some cells to compute the loss), GEPC Loss (i.e., we predict the gene expression levels based on the cell embeddings to compute the loss), ECS Loss (i.e., we bring similar cells closer together and push different cells farther apart in the embedding space to compute the loss), and one task‐specific loss function, known as the gradient reverse loss (i.e., we reverse the gradient of batch label classifier to perform adversarial training). We compared the scGPT without certain loss functions with its default mode to perform ablation tests. Using all components of the loss function did not always yield the best results, with the exceptions of the Pancrm, MCA, and MHSP datasets. Using only the gradient reverse loss function resulted in the worst performance. The GEPC loss seemed to play a crucial role in the performance of the batch effect correction task. These results suggest the need for a careful composition of the loss function when training single‐cell FMs for batch effect correction, with each loss function component contributing differently to model performance.

#### 2.3.2 Multi‐Omic Data Integration

For this task, we intend to integrate unpaired scRNA‐seq datasets with scATAC‐seq datasets. We considered scGPT, scJoint [56], and scGLUE [57] for evaluation. We assessed the integration quality through the same score as batch effect correction because these two tasks have similar targets, that is, integrating datasets from different domains while preserving biological variation. The results presented in Figure 2b summarize the evaluation results for the multi‐omics integration task. Overall, scGLUE surpassed scGPT and achieved the best performance. In addition, Figure S13 shows that the results of scGPT did not have better biology conservation scores than the raw condition across all the datasets. Therefore, there exists a performance gap between scGPT and task‐specific models. Based on our analysis, scGPT still performed better than scGPT v1, which implied that larger pre‐training datasets might help integrate multi‐omic datasets. We also observed a batch effect for the results of scGPT based on UMAP plots shown in Figure S14. In conclusion, single‐cell FMs did not show an advantage in handling datasets for multi‐omic data integration.

We illustrate the effect of initial settings in Figure S11d. Different from the case with batch effect correction, the cross‐entropy loss function led to worse performance compared to the MSE loss for this task. Interestingly, pre‐training did not significantly affect the performance for this task since training from scratch also had similar performance. The encoder part of the single‐cell FM played a more important role than the decoder since we observed a larger performance drop by only freezing the encoder part. Including cell types or human labels in the training process proved beneficial, likely providing the model with more precise and useful information for the task. The zero‐shot learning approach did not perform as well for this task as it did for batch effect correction. Therefore, we need more consideration for the design of single‐cell FMs for this task.

We illustrate the evaluation metrics versus different parameter settings in Figure S15. scGPT did not perform well on this task as shown by the low score (below 0.5), even if we tried to search for better hyperparameters. Certain hyperparameters affected the training process. A smaller weight for the loss function, a larger dropout rate, and more epochs improved the model's performance. The number of bins and mask ratio did not exhibit monotonicity, making it difficult to draw conclusions. Setting a high learning rate and ECS weight decreased the performance of scGPT. Moreover, based on Figure S11e, the optimizers in the Adam family are still preferred because of better performance, which is similar to what we found for the batch effect correction task. Since the patterns discovered in the multi‐omic data integration task are not exactly the same as what we discovered in the batch effect correction task, we believed that specific design focusing on scATAC‐seq data is required.

#### 2.3.3 Cell‐Type Annotation

For this task, we intend to annotate cell types for scRNA‐seq datasets. We considered all open‐source FMs, TOSICA [23], and ${\text{SVM}}_{\text{rej}}$ [58, 59] for this task. We assessed the performance of different single‐cell FMs in assigning cell types based on the four metrics (Accuracy, Precision, Recall, and F1 score) discussed in Appendix D.2. These metrics are widely used in the evaluation for cell‐type annotation.

We first compared the performances of different single‐cell FMs based on the zero‐shot setting for this task, shown in Figure S12. According to this figure, SCimilarity, CellPLM, and UCE have the overall highest annotation accuracy based on logistic regression. Considering SCimilarity was trained with cell‐type labels, introducing cell states in the model training stage might help in performing this task and single‐cell FMs have the potential to work as powerful tools for cell‐type annotation. Moreover, since scGPT also supports fine‐tuning mode for predicting cell types, we also compared the zero‐shot setting and fine‐tuning setting of scGPT for batch effect correction. In Figures S16a,b, we explored the capacity of using cell embeddings from scGPT and Geneformer to annotate cell types (the zero‐shot learning mode) and compared the results with the fine‐tuning mode. We found that on average the fine‐tuning mode surpassed the zero‐shot learning mode for annotating cell types (*p*‐value = 1.9e‐3 for scGPT, *p*‐value = 1.9e‐3 for Geneformer, based on the Wilcoxon rank‐sum test). Therefore, it is still necessary to consider fine‐tuning single‐cell FMs for cell‐type annotation tasks.

We further compared different single‐cell FMs based on their default setting. The UMAPs for the raw data and scGPT are shown in Figures S17 and S18. Since these plots were generated based on the same cell embeddings, they could be used as visualizations for the observed and predicted distributions of cell types. Figure 2c displays the Accuracy for different settings for these models. On average, models with pre‐training performed better than those without pre‐training. This observation and overall rank align well with results of weighted F1 score shown in Figure S19. However, CellLM and scFoundation did not perform well across all the datasets, mainly because these two methods had running errors for Pancrm, HumanPBMC, PBMC368k, and Liver cross‐datasets, which raised the issue of the reliability and usability of these models again. The default setting of SCimilarity also did not perform well in this task, which might be due to their pre‐training process, which contained too many sub‐cell types and only utilized 10X sequencing data [40], and thus the default neighbor‐based sub‐cell‐type annotation approach cannot work well in the real application. Moreover, for the intra‐dataset prediction task, CellPLM, scGPT, and Geneformer were comparable, although they had different pre‐training settings. For the inter‐dataset prediction task, CellPLM, scGPT, Geneformer, tGPT, and UCE were better than scBERT. Therefore, different single‐cell FMs also had large divergences in performance, but CellPLM, scGPT, and Geneformer had good performances across different datasets.

In Figure S20, we compared the performance of models with different hyper‐parameter settings. Higher loss weight, learning rate, ECS threshold, mask ratio, and smaller epochs tended to lead to worse performance of scGPT. There was little correlation between the number of bins and the performance of scGPT. We observed the consistency in the performance of different single‐cell FMs under the condition of altering their shared hyper‐parameters. For Geneformer and scBERT, a lower learning rate and higher epochs also tended to lead to better performance.

We also considered different initial settings for model training. The first setting is *Freeze all*, where we froze all the weights of pre‐trained layers. The second setting is *Default*, where we used the default fine‐tuning settings. The third setting is *From scratch*, where we did not use the pre‐trained weights. Figure S21a shows the score versus initial settings across different datasets. Here we considered scGPT and scBERT. We omitted Geneformer because it requires pre‐training weights as input. It can be seen that pre‐training always improved results for scGPT, especially for the cross‐dataset conditions. However, there was a little benefit of pre‐training for scBERT. For both cases, freezing the pre‐training layers and preventing them from being involved in the fine‐tuning process was not recommended. In some cases, the fine‐tuning performance of such freezing was worse than training from scratch. Transfer learning for different species is possible because, for the MCA dataset, pre‐training based on human data can help predict cell types for the mouse. For the same type of GPU, the training process of scGPT was faster than scBERT and Geneformer, with more GPU memory usage, according to Figure S21b.

In Figure S22, we froze the front layers or scGPT (from 0 to 11) to investigate the relation between the number of freezing layers and annotation accuracy. We found that freezing layers had comparable or even slightly better performance than full‐parameter fine‐tuning. Although the metrics under different numbers of freezing layers are close and high, we still found that freezing five layers can lead the best performance. Therefore, we can freeze parts of layers for cell‐type annotation based on single‐cell FMs.

In Figure S23, we show the performance of CellPLM, Geneformer, scGPT, and scBERT based on different optimizers across four datasets. Overall, Adam, AdamW, and Lion were comparable. Sophia‐G was worse than them but better than SGD, and they were both unstable. Moreover, Geneformer did not support Lion and Sophia‐G as optimizers, and thus the optimizers in Adam family are more preferred in fine‐tuning single‐cell FMs.

Moreover, we explored the contribution of different loss function components toward the cell‐type Annotation task based on ablation tests, and the results are shown in Figure 2d. Here, we included three extra metrics, and details can be found in Appendix D. Based on the Accuracy of Figure 2d, the inclusion of mask loss is important. Moreover, the default setting is generally good across different tasks. Based on precision and recall of Figure 2d, the effect of different loss function terms had less effect on precision and more effect on recall. Such a difference could affect the final F1 score. Removing the GEPC loss function terms improved the cell type prediction for the DC, MHSP, and MB spatial datasets, and did not affect the prediction performance for the other datasets.

Therefore, most single‐cell FMs can handle the cell‐type annotation task with suitable pre‐training data and model structure, but investigation for the pre‐training framework is needed to understand their specific performance differences across all the datasets.

### 2.4 Evaluation Based on Gene‐Perspective Tasks Shows That Single‐Cell FMs can Handle Tasks Related to Functions of Genes

#### 2.4.1 Gene Function Prediction

**FIGURE 3:**
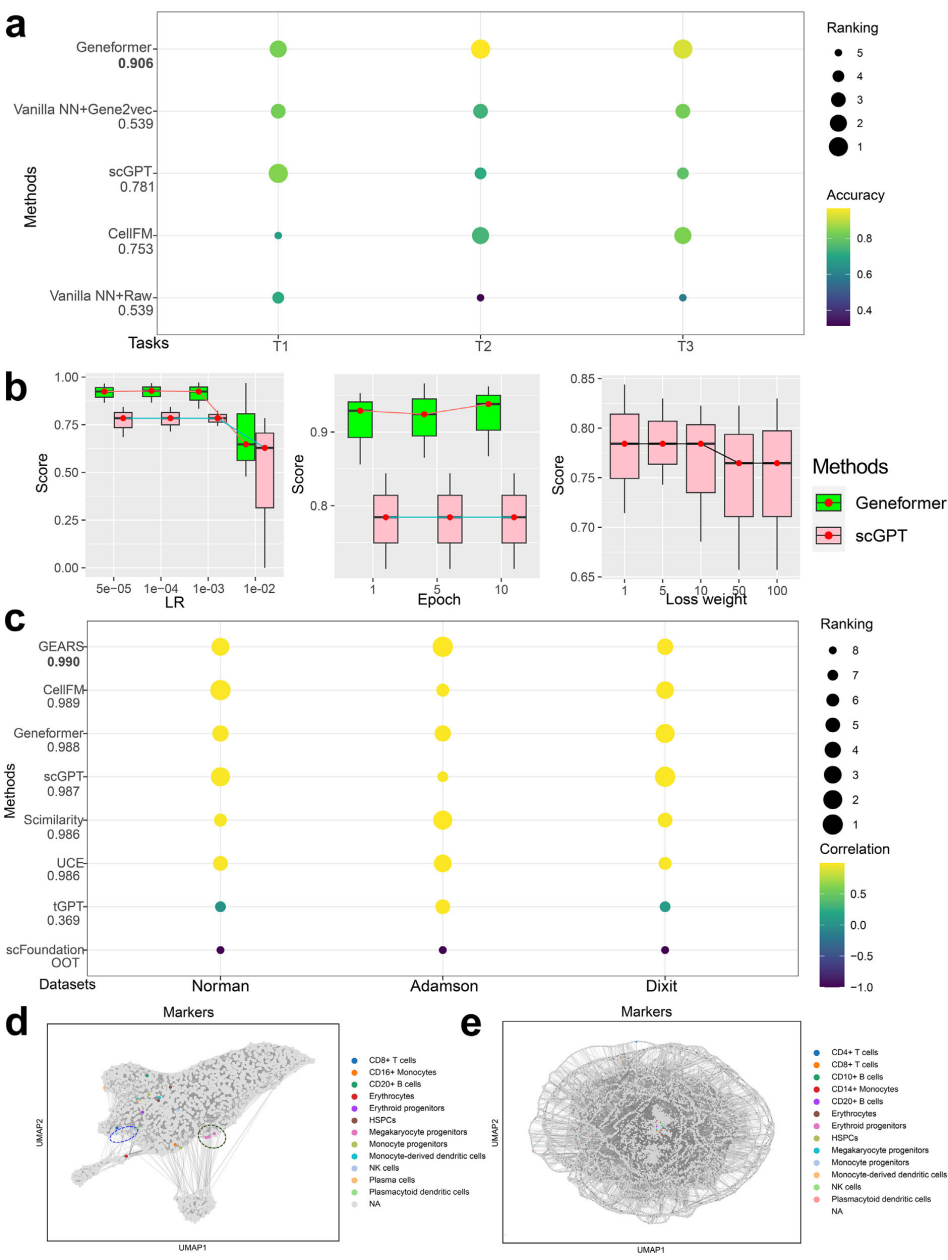
Experimental results of single‐cell FMs and benchmarking methods for gene‐level tasks. (a) Comparisons among Geneformer, scGPT, and *vanilla* NN in the Gene Function Prediction task. (b) The effect of hyper‐parameters including Loss weight, Bins, and Learning rate for scGPT and Geneformer in the Gene Function Prediction task (sample size n=3 per box). (c) Correlation metrics of different methods. A higher correlation means lower rank and better performance. The numbers corresponding to the settings represent the average value across two datasets. (d) Dataset‐level gene embeddings from scGPT colored by the marker genes of different cell types. (e) Dataset‐level gene embeddings from Geneformer colored by the marker genes of different cell types.

For this task, we intend to predict the functions of genes. We considered Geneformer, scGPT, scFoundation, CellFM, and vanilla NN based on raw expression data and vanilla NN based on Gene2vec [60] for this task. scFoundation met running errors while generating the gene embeddings for datasets used in this task. We split the genes for training/testing and evaluated the results with the same four metrics as the cell‐type annotation task because they are both classification problems. The results are shown in Figure 3a. On average, Geneformer and scGPT performed well in this task. Moreover, the accuracy scores of scGPT and vanilla NN based on Gene2vec were comparable, while there was a performance gap between single‐cell FMs and *Vanilla* NN based on raw data. Therefore, using initial gene embeddings with prior information is meaningful for single‐cell FMs.

Figures 3b and S24 show the accuracy of different hyper‐parameter settings. Smaller learning rate and loss weight tended to have more accurate results. Geneformer was more sensitive to Epoch compared to scGPT. For scGPT, pre‐training contributed more than fine‐tuning in this task as increasing epochs did not affect the model performance. Only tuning the number of bins, mask ratio, dropout rate, and ECS threshold did not affect the prediction results.

In Figure S25a, we considered different initial settings for model training. It can be seen that pre‐training always improved performances of scGPT. Meanwhile, freezing the whole model did not affect the performance of scGPT.

Figure S25b shows the performance of scGPT based on different optimizers. Adam and AdamW were comparable, while Lion was worse than them but better than SGD and Sophia‐G.

Figure S25c shows the ablation test results of scGPT for this task. There was no significant difference by comparing the default setting and those without certain components. Therefore, the task‐specific loss function is the most important design for this task.

#### 2.4.2 Perturbation Prediction

For this task, we intend to predict the gene expression levels under perturbations. We considered scGPT, Geneformer, tGPT, SCimilarity, UCE, scFoundation, CellFM, and GEARS [61] for this task. We implemented a linear‐regression‐based model to predict perturbed gene expression levels from cell embeddings for those FMs without this function. We used mean Pearson correlation (MPC) and mean squared errors (MSE) as the metric to evaluate the performances across all genes, and details can be found in Appendix D. Therefore, we can assess the prediction performance at the gene level for both perturbed genes and unperturbed genes. The datasets include two perturbation conditions: a single‐gene perturbation and a double‐gene perturbation, and two gene sets: all gene sets and differentially expressed genes (DEGs).

We first compared the performances of different single‐cell FMs based on the zero‐shot setting for this task, shown in Figure S26a for MPC and (b) for MSE. According to these figures, Geneformer, scFoundation, and scGPT have the best perturbation prediction performance based on cell embeddings. Other methods such as tGPT show very high MSE for predicting perturbation effect based on the Norman and the Dixit datasets, implying that not every single‐cell FM has good embeddings to perform this task. Moreover, since scGPT supports the fine‐tuning mode for perturbation prediction, we also compare the MPC between the zero‐shot mode and fine‐tuning mode based on scGPT for perturbation prediction, shown in Figure S5b. This figure implies that there is no big difference between predicting the perturbation effect directly from cell embeddings or with the fine‐tuning setting based on scGPT, and thus cellular embeddings from these single‐cell FMs might be helpful for developing tools focusing on addressing problems from perturbation prediction.

We further compared single‐cell FMs and task‐specific methods based on their default settings. Based on our experiments shown in Figures 3c and S27, most of the FMs (except tGPT) and GEARS were comparable in this task. By evaluating the model performances based on DEGs shown in Figures S28a,b, we find that GEARS still outperforms the rest of single‐cell FMs, followed by scGPT. Geneformer, SCimilarity, and UCE have similar performances. Moreover, the running time of scFoundation for this task was too long to finish and tGPT is not a suitable predictor, which suggest the challenges of deploying FMs in solving this task. Since GEARS has robust performance, the requirement of developing FMs for handling such task is not clear. To understand the contributions of single‐cell FMs in this task, we further conducted statistical analyses by comparing the performances (PCC or MSE) of different methods with one‐sided Wilcoxon Rank Sums test, and we visualize the −log(p−value) in Figure S29a,b. Here we found that GEARS single‐cell FMs generally cannot surpass GEARS significantly, but fine‐tuning models can generally benefit this task. Therefore, we believe that dataset‐specific adaption is still meaningful for this task and task‐specific expert models such as GEARS are recommended.

Figure S30 also summarizes results for different initial settings of scGPT for the Norman, Adamson, and Dixit datasets. The default setting performed best for these datasets across different settings. This indicates that the initial configuration of scGPT works well for this task. The performance was comparable between training from scratch and training from pre‐trained weights. Freezing the decoder part of the model performed better than freezing the encoder part, which implied that the encoder part is important for perturbation prediction. Interestingly, the ability of zero‐shot learning of scGPT towards this task was not suitable. Therefore, the contribution of using non‐perturbed data as pre‐training datasets for perturbation prediction related to perturb‐seq datasets is not significant. We may need to investigate more types of perturbation, including drug‐level conditions or disease‐level conditions, to check the importance of introducing prior information for perturbation analysis.

Regarding the effect of hyperparameters, Figure S31 shows that scGPT is sensitive to adjusting the learning rate and epochs. Decreasing learning rate and increasing the number of epochs improved MPC. Higher learning rate caused a running error for scGPT. We did not identify patterns for other hyperparameters. Figure S32a shows that AdamW, Sophia‐G, Adam, and Lion had comparable performance for scGPT in perturbation prediction. SGD could significantly reduce the performance of scGPT. Therefore, the use of single‐cell FMs for perturbation prediction does not require a complicated design.

#### 2.4.3 Gene Network Analysis

For this task, we intend to evaluate the quality of gene networks inferred from gene embeddings. We considered scGPT, Geneformer, CellFM, and scFoundation in this section. First, we clarified the difference between gene regulatory networks (GRNs) and gene co‐expression networks (GCNs), and the networks we evaluated here were GCNs. In general, GRNs characterize causal relationships among genes. The GRNs defined in scGPT are in fact GCNs, because the construction process is based on embedding similarity. For the inference of GCNs, two types of them can be defined: Type 1 GCN (Tissue‐specific GCN) and Type 2 GCN (Cell‐type specific GCN). We used the Immune Human Atlas dataset to evaluate the performance of inferring these two types of GCNs. The known information including marker genes [62], cell types [62], and Reactome pathways [63] was utilized to evaluate the performance of scGPT on the GCN inferences. We also included scWGCNA [64] as a task‐specific method for comparison.

Type 1 GCN is generated by applying the single‐cell FM to the entire dataset under a zero‐shot learning framework to create gene embeddings. The similarity is then computed to infer gene‐gene relationships based on these embeddings. The quality of the GCN is evaluated based on the relationships between marker genes for different cell types.

Type 2 GCN is created by applying the single‐cell FM to generate cell‐type‐specific gene embeddings under the zero‐shot learning framework, and the same type of similarity is used to infer gene–gene relationships based on these embeddings [36]. The quality of this GCN is evaluated based on the gene ontology enrichment analysis (GOEA) [65] for specific gene sets. These GCNs can provide valuable insights into the understanding of gene interactions and regulation in specific tissues or cell types, which could have broad applications in biology and medicine [66, 67].

In the analysis of the Immune Human Atlas dataset, we initially focused on Type 1 GCN, with the results presented in Figure 3d,e). The neighboring relationships within this dataset are colored according to the distribution of marker genes. We collected marker genes based on the source paper of this dataset [62] and filtered them to have marker genes with similar expression patterns for each cell type. As the gene‐gene relationship was determined based on *k*‐nearest neighbors, it can be viewed as a form of gene co‐expression relationship. From Figure 3d, only marker genes from two cell types showed the co‐embedded and isolated relationship. They are Monocyte‐derived dendritic cells and Megakaryocyte progenitors. Figure 3e shows that the embeddings from Geneformer for maker genes were all co‐embedded. Therefore, the embeddings of scGPT are better than the embeddings of Geneformer on preserving the cell‐type‐specific information. Figure S33a,b, on the other hand, represent the cluster labels based on the Leiden clustering method [68]. These clusters can be interpreted as groups of genes that share common functions, or “gene co‐function clusters.” We first analyzed the cluster information from the gene embeddings from scGPT. For marker genes from other cell types, some of them are in different clusters shown in these two figures, and some genes are co‐embedded with other cell types' marker genes. There are two isolated groups (9 and 12), but no marker genes are identified in these two groups. For the gene embeddings from Geneformer, we found that most of the marker genes from different cell types are embedded in the same cluster, while the rest of clusters did not contain much cell‐type‐related information. Therefore, the clustering result of scGPT was also more biologically meaningful than Geneformer, possibly due to the number of selected genes. Geneformer generated all genes for embeddings by default, while scGPT only focused on highly variable genes.

We also evaluated and explored GCNs based on the human immunology system quantitatively, which is known for its complexity due to interactions among various cell types. The original analysis of GCN from [36] focused on HLA genes and CD genes. We did not analyze HLA genes because they are highly polymorphic and thus carry a higher risk for having errors of reads [69, 70, 71, 72], so the network result may not be reliable. Therefore, we selected genes co‐embedded with CD3 genes, and utilized the embeddings of these genes to compute the GCNs. The results of scGPT and Geneformer for CD3‐related genes are shown in Figure S33g,h. The value above the edges represents the strength of correlation. To visualize the important interaction, we use the threshold of strong correlation from scGPT (0.4). A publicly accessible database containing pathway information—the Immune System R‐HSA‐168256 pathway from the Reactome 2022 database [63]—was used as a reference for validation. The GCN was constructed based on the correlation between the two genes. For the CD3‐related genes, there was nearly no overlap between discovered pathways and the whole set (7/1943) for scGPT, Geneformer (8/1943), CellFM (0/1943), and scFoundation (0/1943), indicating poor inference of GCN. However, the results of scWGCNA have very high overlap (913/1943), and thus traditional method better captured pathway‐specific information from genes. To ensure the conclusion is consistent for GCNs under different resolutions, we also adjusted the resolutions, and Figure S34a,b shows that scWGCNA's overlap was always higher than the overlap from other single‐cell FMs. To explore the similarity of gene embeddings from singlecell FMs, we visualize the detailed pathway information from scGPT and Geneformer in Figure S35a,b, and there is no overlapped pathways between the results from the two models. Moreover, for all the pathways involved in the selected gene set, we computed the ratio between the number of significant pathways and all pathways, and recorded the results in Figure S33f. We found that gene embeddings from scGPT had a higher ratio, suggesting a better quality of scGPT output. These analyses highlight single‐cell FMs might not contribute much to construct meaningful GCNs due to the insufficient quality of gene embeddings.

Figure S33c,d focuses on gene embeddings categorized by cell types from scGPT, while Figure S33e shows the cell‐type‐specific gene embeddings from Geneformer. By treating cell types as observed labels, we also computed normalized mutual information (NMI) and adjusted Rand index (ARI) for gene embeddings from different methods. Figure S33c,d shows that gene embeddings of scGPT (NMI = 0.049, ARI = 0.035) from different cell types tended to be co‐embedded and there was no apparent difference. The distribution of the remaining genes on the UMAP results was relatively random, and we also observed a random distribution of gene embeddings from Geneformer (NMI = 0, ARI = 0). Furthermore, the NMI and ARI scores of two types of gene embeddings are also low. One reason could be that the quality of gene embeddings was unsatisfactory. Since scGPT and Geneformer adopt the zero‐shot learning scheme embeddings, it does not incorporate cell‐type‐specific information for a specific dataset. The other reason might be that the complex biological network in the human immune system makes the communication between cell–cell or gene–gene difficult to decompose [73, 74]. Additional analysis is needed for generating gene embeddings. This analysis illustrates the complexity of cellular functionality and the difficulty of clearly defining these relationships based on gene embeddings. Despite these challenges, the scGPT model still demonstrates its potential in identifying functional similarities between different cell types.

In conclusion, our results highlight the importance of critical evaluation and cross‐referencing in the development of GCNs inference, as well as the potential and limitations of using single‐cell FMs for this purpose.

### 2.5 Evaluation Based on Imputation and Simulation Analysis Shows That Further Improvement of Single‐Cell FMs is Needed

#### 2.5.1 Imputation

For this task, we intend to impute gene expression profiles of different datasets. We considered imputation for two different types of datasets, known as scRNA‐seq datasets and spatial transcriptomic datasets. To impute scRNA‐seq datasets, we intend to evaluate the performances of single‐cell FMs for filling technical zeros, which contain ∼ 20 000 genes. To impute spatial transcriptomics, we intend to evaluate the performances of single‐cell FMs for filling ∼ 20 000 unobserved genes, which are observed in scRNA‐seq data. We compared CellPLM, scGPT, and Tangram [75] in this task. The evaluation metrics for this task based on clustering and correlation are provided in Appendix D. The evaluation of clustering performance and gene expression correlation can assess the preservation of biological variation after imputing. The imputation results for scRNA‐seq are summarized in Figure S37a, which implies that the imputation function of scGPT for scRNA‐seq data introduced more noise into the original sequencing data, suggesting the unreliability of the decoder's output.

According to Figure S37b, scGPT performed well in the spatial transcriptomic data imputation task compared to the SOTA spatial imputation method, Tangram [75, 76]. Moreover, CellPLM did not outperform Tangram evaluated based on cell clustering with biology conservation score and correlation. Its proportion of significant genes was lower than scGPT. Therefore, CellPLM might overfit scRNA‐seq data under this task setting. Based on the evaluation of correlation and significance proportion, the imputation results of scGPT are better than the results of Tangram. Moreover, the scores of these two metrics based on the zero‐shot learning version were even better than the pre‐training version with scRNA‐seq data. However, based on the results of the average bio score evaluation, the raw data had better scores. This could be caused by the sources of the spatial clustering labels, which were generated from gene expression clusters rather than expert annotation. Such methods could introduce bias before and after imputation.

Figure S38 shows the results of DEGs based on pre‐imputation data and post‐imputation data. Results in Figure S38a showed that scRNA‐seq imputation was not reliable because the expression patterns of all genes were similar after imputation based on scGPT. However, based on Figure S38b, no mitochondrial (MT) genes were included in the DEGs after imputation based on scGPT. These genes were identified as DEGs in the raw dataset. A high proportion of MT genes is indicative of low‐quality data, which means cells with such patterns are apoptotic or lysing [77]. Therefore, the MT genes should be omitted in the downstream analysis by filtering [78, 79]. Moreover, based on Figure S38b,c, the DEG patterns after imputation based on scGPT and Tangram are similar. Thus, scGPT has the potential to produce biologically meaningful imputation for spatial transcriptomic datasets.

#### 2.5.2 Simulation Analysis

For this task, we intend to simulate synthetic scRNA‐seq datasets. We considered scGPT, Splatter [80], and scDesign3 [81] for this task. We evaluated the output of scGPT against the output of scDesign3 and Splatter. For conditions incorporating batch effects, we employed the same metrics used in the evaluation of batch effect correction. In scenarios without batch effects, our metrics are primarily focused on assessing the preservation of biological information. As shown in Figure S37c– e, scDesign3 outperformed scGPT and Splatter across two conditions of the simulation task. However, scGPT performed better than Splatter for simulating datasets without batch effect. In particular, scDesign3 had a more pronounced superiority in generating simulation data without batch effects, in comparison to scGPT. This is consistent with the results shown in Figures S37d,e and S39. The gene–gene correlation from scDesign3 was also more similar to the gene–gene correlation of the raw data. The gene–gene correlation from scGPT has null value due to missing the gene expression certain genes, which is a problem caused by the decoder outputs from scGPT. Therefore, the simulation task needs to be improved for single‐cell FMs as reference‐based simulators.

In addition, we present UMAPs of the output produced by different methods in Figures S40 and S41 that illustrate the advantage of scDesign3. The embeddings of scGPT with the no batch effect settings tended to preserve the batch effect, while the embeddings with batch effect tended to remove the batch effect. The embeddings of Splatter with no batch effect also mixed different cell‐type information.

### 2.6 Model Scaling Contributes to Single‐Cell FMs but Their Stability Need to be Improved

#### 2.6.1 Exploring Model Scaling Analysis

In this section, we explored the contributions of model scaling based on CellPLM, scBERT, Geneformer, and scGPT. These methods have finetuned versions and do not suffer from running errors, and also demonstrate promising performances based on our previous evaluation. The scaling law implies that models with large‐scale parameter size might have better performances for certain tasks [82, 83, 84]. Due to computational constraints, we are unable to test scaling effects within a single model family and instead investigate trends across models with different parameter scales and configurations. Here, we considered three scenarios to investigate the possible contribution of model scaling: cross‐data cell type prediction, cross‐species analysis, and spatial transcriptomic analysis. Our hypothesis is that models have task‐specific benefit from scaling the computational scales of models, which is summarized based on our previous observations and analyses.

Inspired by [37], we compared models with large parameter size of pre‐training to models of small parameter size. For the task of cross‐data cell type prediction, we compared other single‐cell FMs with Vanilla neural networks (NNs) to identify any contributions of model scaling. For the task of cross‐species analysis, we compared other single‐cell FMs with expert model SATURN [85]. As for the task of spatial data batch effect correction, we examined the statistics derived from our correction evaluation to verify our hypothesis and the expert methods include ResPAN and Novae [86]. Novae is an FM pre‐trained with spatial transcriptomics data and thus it is a meaningful baseline to evaluate the contribution of transferring knowledge from single‐cell transcriptomics to analyze spatial transcriptomics.

**FIGURE 4:**
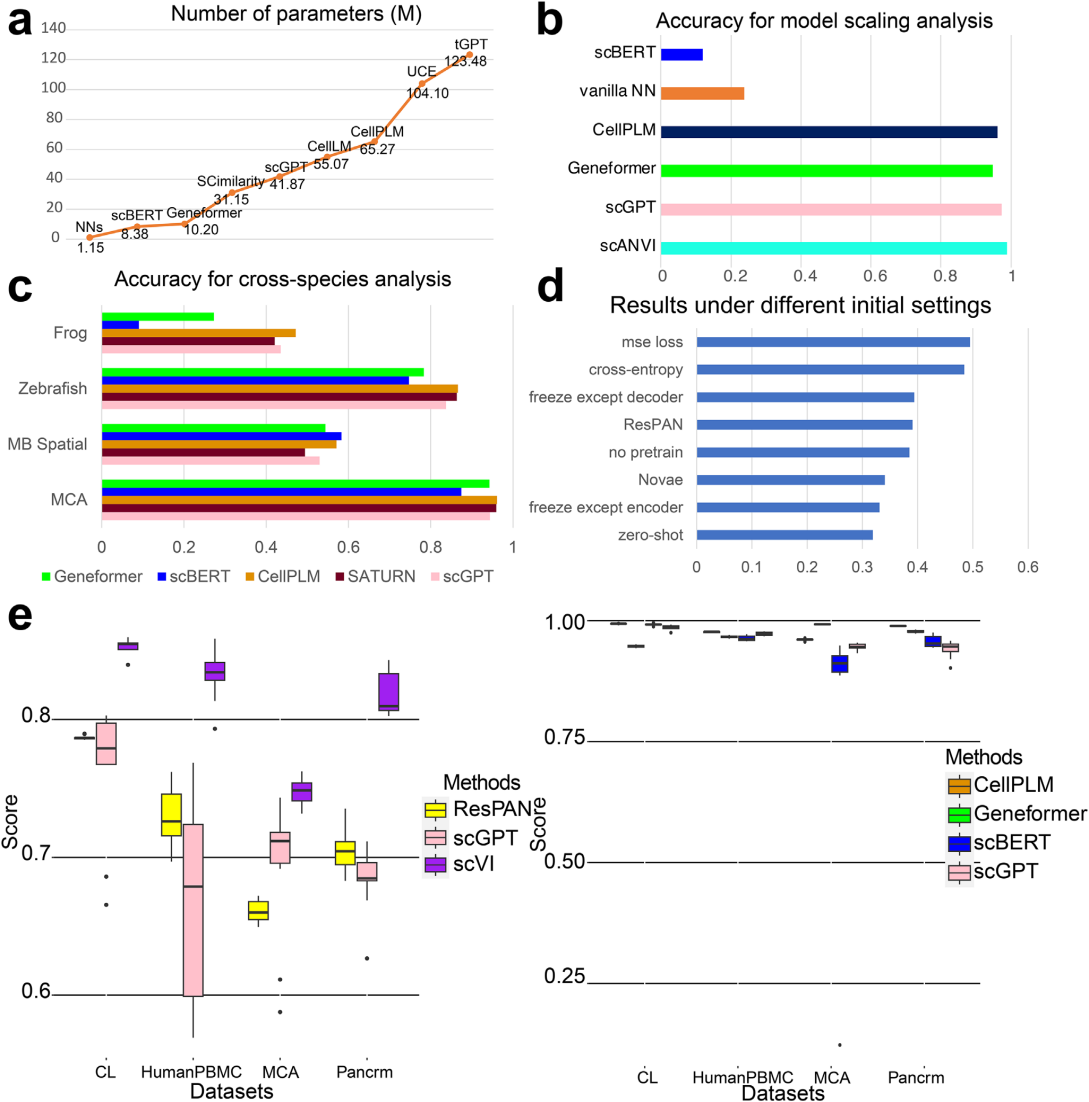
Different comparison groups for model scaling analysis and stability analysis. (a) The model scale of different methods, defined by the number of parameters (unit: Million). (b) Accuracy of FMs and *vanilla* NN in cell‐type annotation task. The dataset here is the Pancreas cross‐dataset. (c) Accuracy of FMs and SATURN in cell‐type annotation task across species. (d) Overall score comparison including ResPAN, Novae and different settings of scGPT. The dataset here is the human spatial transcriptomic dataset. (e) Different batch correction scores of different models based on changing random seeds (left) and different average classification scores of different models based on changing random seeds (right). The bold black line represents the median value, while the length of each box can be interpreted as the variance level (sample size n=10 per box).

In the first scenario, we compared single‐cell FMs to *vanilla* neural networks (NNs) with smaller parameter counts. Figure 4a presents model sizes, and Figure 4b shows their performance. We observed that scGPT and Geneformer outperform *vanilla* NNs and scANVI [87], suggesting that, in some cases, models with parameter counts above 10 million may yield performance gains. However, this trend is not uniform: scANVI, which is a smaller yet specialized model, performs comparably or even better than some larger single‐cell FMs (e.g., scBERT), indicating that architecture and task‐specific design remain key performance factors. Thus, while model scale may contribute positively in certain scenarios, it is not the sole determinant of effectiveness.

In the second scenario, the desired contribution of model scaling mirrors that of the first task. To perform cross‐species study, we only focus on orthologous genes across different species, which are extracted based on the Ensembl database [88] and the proportion is recorded in Figure S42. Nonetheless, there was no enhancement in performance from Figure 4c. The cross‐species cell‐type annotation task is difficult, also suggested by [85, 89], as a representative task in analyzing cross‐species information transition. Figure 4c shows that SATURN is a strong baseline and it outperforms single‐cell FMs such as Geneformer and scBERT, while is comparable with scGPT. However, CellPLM can still outperform SATURN in most of the datasets used for cross‐species analysis. Therefore, single‐cell FMs, including CellPLM and scGPT showed advantages in this task.

In the third scenario, we considered two possible tasks. First, in line with the batch effect correction task, we postulated that using Human scRNA‐seq data for pre‐training could aid in batch effect correction for human spatial transcriptomic data. Second, resonating with the cell‐type annotation task, we hypothesized that pre‐training on Human scRNA‐seq data might assist in cell‐type annotation for mouse spatial transcriptomic data. Figure 4c shows that we did not detect any contributions of model scaling in the MB Spatial data annotation task. Figure 4d suggests the contribution of model scaling for batch effect correction. scGPT under different loss functions all outperform Novae, while Novae performs better than the zero‐shot mode of scGPT in batch effect correction. Therefore, including single‐cell transcriptomics and spatial transcriptomics data jointly for model training can contribute to spatial transcriptomics analysis in batch effect correction. The fine‐tuning process appeared beneficial in reducing the batch effect inherent in the spatial data, whereas scenarios with model freezing except decoder yielded subpar results. The performance of scGPT in the integration of spatial data was better than that of ResPAN. Therefore, we observed the potential contribution of model scaling in the batch effect correction for spatial transcriptomic data.

#### 2.6.2 Stability Analysis

To analyze the stability of single‐cell FMs, we selected Batch Effect Correction and Cell‐type Annotation as two representative tasks and varied the seeds from 0 to 9 of single‐cell FMs to investigate the model stability. All of the methods used in this section are fixed with their default hyper‐parameters. These two tasks are the main tasks in single‐cell data analysis and have solid metrics for evaluation. Ideally, the results of different single‐cell FMs should not vary substantially across different datasets. We also considered stability for other benchmarked tools. Our experiment results summarized in Figure 4e showed that the stability for single‐cell FMs is task‐specific.

Based on the left panel of Figure 4e, the variance of scVI [90] and ResPAN was generally lower than that of scGPT, and scVI and ResPAN also had a higher score on average. Therefore, single‐cell FMs were not as stable as SOTA deep‐learning‐based methods for the batch effect correction task.

The right panel of Figure 4e suggests that the variance of Geneformer and CellPLM was generally smaller than that of scGPT and scBERT. All four models had high median average scores. Moreover, the variance of scBERT was relatively large in the experiments based on the MCA dataset, which implies that single‐cell FMs might fail under certain random seeds.

The statistical analyses of pairwise comparison in this section are shown in Figure S43a,b, where expert models have significantly better performances in batch effect correction across different initialization stages and datasets, while CellPLM and Geneformer have generally good performances in annotating cell types across different initialization stages and datasets.

## 3 Discussion

In this paper, we have evaluated the performance of 10 single‐cell FMs on eight different tasks for single‐cell data analysis. The ranks of these single‐cell FMs are shown in Tables 1 a,b, where we not only considered the broader functions of the models, but also their usability. Based on our evaluation results, open‐source models have higher ranks than closed‐source models. Open‐source models with tutorials are more friendly to researchers, and these models also receive a number of stars and likes based on Figure S44. Tutorials associated with these models also enhance their accessibility to researchers.

Based on our experimental results, the performance and contribution of single‐cell FMs are task‐specific. For the Batch Effect Correction and Multi‐omic Data Integration tasks, single‐cell FMs did not outperform task‐specific SOTA methods. Moreover, the correction results of atlas‐level datasets were worse than those of the raw datasets. For the Cell‐type Annotation task, we found that CellPLM, scGPT, and Geneformer had comparable performance. Moreover, these methods were better than the other models in this task. For the Gene Function Prediction task, Geneformer also performed well and better than classical models for prediction. We also showed that the pre‐trained model can have better performance in this task. For the Perturbation Prediction task, the function of model pre‐training is not apparent, and GEARS is comparable with other single‐cell FMs in this task, questioning the need of the necessity for developing single‐cell FMs to understand perturbations. For the Gene Network Analysis task, we did not observe the great contribution of gene embeddings from single‐cell FMs for either tissue‐specific or cell‐type‐specific analysis. Constructing the cell‐type‐specific gene networks is still challenging for single‐cell FMs reflected by the low clustering scores, and thus it is meaningful to explore methods for improving the function of network discovery. For the Simulation task, scGPT also did not perform very well. scDesign3, as a task‐specific method, is better for simulating reference‐based scRNA‐seq datasets. For the Imputation task, we showed that scGPT did not perform well in the scRNA‐seq imputation task, but it outperformed Tangram in the spatial transcriptomic data imputation task both under zero‐shot learning and fine‐tuning frameworks. Such a finding suggests that single‐cell FMs can transfer knowledge across different omic data. Furthermore, we observed the contributions of large‐scale parameters for single‐cell FMs in specific tasks. For the cross‐data and cross‐species cell‐type annotation tasks, the performance of single‐cell FMs is much better than the performance of baselines with smaller parameter sizes. Moreover, scGPT can also be used to analyze spatial transcriptomic data for batch effect correction, which has attracted much attention in recent years [91]. In our stability analysis, we found that single‐cell FMs were not very stable in the batch effect correction task. Moreover, in the evaluation for the Cell‐type Annotation task, we found that different single‐cell FMs had different variance levels. The variance under different random seeds was driven by datasets. Considering the problems of stability, we still have difficulty refining it into a toolbox (e.g., Seurat [92] or Scanpy [79]) with various functions.

Therefore, by considering the definition of a foundation model, there are evident limitations in their construction and training steps. A more comprehensive understanding of such FMs is necessary to address these issues. In this manuscript, we tried different approaches to discover insights from model components and training settings to possibly enhance the performances of single‐cell FMs. Our conclusions, combined with the recommendations of models for different tasks, are summarized in Table 2. Considering the difference in resources required for zero‐shot learning and fine‐tuning, as well as the difference in model effectiveness, we suggest trying to apply the model's zero‐shot learning mode to obtain the embeddings required for the task at first, and then consider fine‐tuning model for the given task if the performance of zero‐shot learning mode is not satisfactory and we can obtain a good task‐specific loss function. Based on our analyses, the task‐specific loss function component is always the most important loss function component, and the second‐most‐important loss function is mask loss, which helps the model reconstruct the original expression values. The rest of loss functions are not as informative as the previously mentioned choices. We also explored more advanced parameter‐efficient fine‐tuning (PEFT) frameworks such as LoRA [93] for scGPT in Batch Effect Correction and Cell‐type Annotation. Figure S45 shows that LoRA slowed down the fine‐tuning process and did not improve related scores. Thus, the training of single‐cell FMs is more nuanced than that of general FMs. While it is important to consider the similarities between these two model types, we must also consider the differences rooted in the datasets and domain‐specific knowledge.

**TABLE 2:** Insights from the benchmarking results of scEval. We organize the table by tasks and model settings.

Topic	Summary	Recommendation
**Applications**		
Batch Effect Correction	Overall, all scFMs performed worse than task‐specific method.	Selecting task‐specific method (Harmony) as a starting point.
Multi‐omic Data Integration	Overall, all scFMs performed worse than task‐specific methods.	Selecting task‐specific method (GLUE) as a starting point, and including multimodal information in the pre‐training stage of scFMs.
Cell‐type Annotation	Overall, finetuned scFMs performed well in this task.	Exploring scGPT, CellPLM and Geneformer for annotating cell types, and we encourage researchers to include cell states in the pre‐training stage.
Gene Function Prediction	Overall, finetuned scFMs performed well in this task.	Exploring Geneformer for predicting gene functions.
Perturbation Prediction	Overall, the performances of scFMs are close to task‐specific methods.	Selecting task‐specific method as a starting point, and including perturbation information in the pre‐training stage of scFMs. Moreover, exploring the prediction based on zero‐shot cell embeddings is also attractive.
Gene Network Analysis	scFMs are not good enough for modelling gene interaction networks.	Starting from gene expression profiles rather than gene embeddings for network inference and analysis.
Imputation	scFMs help on imputing spatial transcriptomics, while do not perform well in imputing scRNA‐seq data.	Exploring the ability of scGPT for imputing spatial transcriptomics with a reference‐free design.
Simulation	scFMs performed worse than task‐specific methods for simulating scRNA‐seq datasets.	Selecting task‐specific method (scDesign3) as a starting point.
**Properties**		
Scaling Law	scFMs followed scaling law for cell‐type annotation.	Investigating the setting of biological questions before enlarging model size.
Stability	scFMs performed inconsistently in batch effect correction, while performed consistently well in cell‐type annotation.	Exploring different strategies for pre‐training scFMs, and developing functions from cell embeddings or gene embeddings.
Training Strategies	Smaller learning rate (e.g. 1e‐4) can improve model performance. During the finetuning process, freezing part of models can also improve efficiency.	Using small learning rate and exploring Parameter‐Efficient Finetuning methods.
Optimizers	Optimizers in Adam family perform better based on our evaluation.	Using Adam or AdamW for model training.
**Deployment**		
Hard‐Ware Requirement	We need at least one GPU with over 40 GB MEM for finetuning.	Using NVIDIA A100/A40/A6000 for deployment.
Model Development	scFMs vary greatly in the extent to which they are open source and the number of features they have by default.	Investigating scGPT, Geneformer and CellPLM for scFM development and deployment.

The application of FMs to single‐cell data remains a promising avenue of exploration, given its impressive performance in prediction tasks. Since much has been done to optimize general FMs [94, 95, 96, 97] (including efficient tuning, model compression, and other research directions), we focus specifically on how to better train and apply single‐cell FMs. Here, we discuss several future directions:

In terms of pre‐training preparation, we need high‐quality data spanning different contexts, such as various cell types, disease states, genders, and even data from different species for pre‐training datasets. High‐quality pre‐training datasets are important for general FMs; otherwise, the performance may be reduced [98, 99]. The qualification of pre‐training data can also be verified with the online learning framework [100]. We can evaluate the performance of single‐cell FMs on downstream tasks and then decide which datasets to include. With a better pre‐training design, we may increase the scale of current models to a billion level. Moreover, the incorporation of biological information is crucial for the success of a FM in biology. Therefore, it is possible to incorporate other biological factors, including GRN and cell–cell interactions (CCI) [26] in the modeling process. With such information, we can also develop domain‐specific or tissue‐specific FMs for single‐cell data analysis. Including extra labels in the pre‐training stage might also improve the model's ability in identifying gene networks under specific context. Exploring the contribution of multimodal data to develop a multimodal FM is also a possible track. For example, incorporating text‐based biological information [101] or multi‐omic data with new tokens may help us further extend the functions of these FMs.

As for model training, both the pre‐training and fine‐tuning steps of the existing single‐cell FMs need improvement. During model pre‐training, we should focus on incorporating biological information or human feedback into the process, as opposed to just relying on the conventional masked token prediction task. For instance, integrating cell‐type or disease condition information into the pre‐training step is an intriguing approach, and it allows experts to evaluate the quality of model output during the training process, which is also meaningful. Moreover, we should also consider the security or trustworthiness of FM training [102]. Using poisoned training single‐cell datasets (e.g., wrong data or make‐up data) as an attack can test the robustness of single‐cell FMs. For model fine‐tuning, instruction tuning [103] is a potential direction to explore. In this context, cells could be considered as prompts, as described in scGPT. Another possible direction is to focus on generating unified embeddings for cells/genes and combining them with task‐specific models for downstream applications, inspired by [37, 104].

For model evaluations, we need more effective methods to assess results for certain tasks. Our recommendations for researchers to consider as evaluations in developing new single‐cell FMs are summarized in Appendix C. For instance, we may include verification from biology experiments to avoid the harm of incorrect output of single‐cell FMs (also known as FM hallucinations [105]). Furthermore, interpretability is also an important perspective we need to consider for a comprehensive evaluation. Methods such as SHAP [106] and Integrated Gradients [107] can interpret the contributions of genes during the pre‐training or fine‐tuning stage, which can be used to evaluate if scFMs are learning the correct biological rules or not. Moreover, analyzing the contributions of model scaling of single‐cell FMs is also important to explore the breakthrough contribution and significance of a model, though we have not identified a task that can only be addressed by developing single‐cell FMs in our evaluation. In addition, we should also account for the pre‐training costs (such as training time and power consumption), and avoid developing a model that consumes large resources but has no significant improvement for downstream tasks.

When it comes to task selection, we should first define the tasks rigorously. For example, in the GRN inference task defined by scGPT, we cannot treat a co‐expression network based on gene embeddings the same as a gene regulatory network. Also, from scGPT, using attention, we can infer gene‐gene correlation strength with direction, but the relation between the correlation of features and the value in the attention map is in debate [108, 109, 110]. We should also consider more meaningful, hardcore, and challenging tasks related to single‐cell and spatial data for single‐cell FMs [111]. These tasks typically need prior information from large‐scale transcriptomic data. For instance, tasks such as in‐silico treatment analysis [35], complex perturbation analysis in the single‐cell levels [112], and spatial data imputation [113] are difficult to handle without prior information, thus provides an opportunity for FMs to make a difference.

The study of benchmarking single‐cell foundation models also has limitations. The first potential drawback is the timeliness of the results. The results of the comparison may change as the model is enhanced. The second drawback is the validity of the dataset. Future base models may use the data in this paper for training, so new datasets will need to be found to evaluate the performance of new models. Finally, benchmarking studies should ideally have community support for reproducing results and extensions.

In summary, our goal in studying single‐cell data‐based FMs is to develop a large‐scale model that is capable of performing multiple tasks with stable and reliable results. Such a model should also be user‐friendly, with detailed tutorials and well‐maintained websites. Our results indicate that there is much space for improving single‐cell FMs. Although our evaluation is subject to the number of current single‐cell FMs, the scale of current single‐cell FMs, and the strategies of pre‐training, we hope that our analysis can provide insights into the best practices and guide the development of future FMs for single‐cell data analysis.

## 4 Materials and Methods

### 4.1 Problem Definition

We consider a pre‐trained FM, denoted as M(x,θ), which is based on the single‐cell dataset D. Here, θ embodies the set of both model parameters (e.g., network weights) and hyper‐parameters (e.g., epochs and learning rate). Different FMs have used distinct pre‐training datasets. The model structure for the fine‐tuning phase is defined as $M^{\prime }\left(x,\theta^{\prime }\right)$. Our objective is to ascertain the optimal set of $\theta^{\prime }$ for various sub‐tasks. Formally, we denote the loss for task k as $L_{k}\left(\cdot ,\cdot \right)$, and use the evaluation dataset $D_{\mathrm{eval}}={\left\{x_{i},y_{i}\right\}}_{i=1}^{n}$, to assess $L_{k}$. Our primary goal is to find

$$\theta^{*}=\underset{\theta^{\prime }}{arg min}E_{x,y\in D}\left[L_{k}\left(M^{\prime }\left(x,\theta^{\prime }\right),y\right)\right].$$

Our second goal is to evaluate the performance of different single‐cell FMs, that is, we intend to find

$$M^{*}=\underset{M^{\prime }}{arg min}E_{x,y\in D}\left[L_{k}\left(M^{\prime }\left(x,\theta^{*}\right),y\right)\right].$$

Our third goal is to assess other abilities of single‐cell FMs, including: (1) zero‐shot learning; (2) model scaling [82, 83, 84]; (3) cross‐species data analysis; (4) biological mechanism exploration; and (5) stability.

### 4.2 Parameters and Tasks

Most single‐cell FMs share the pre‐training process. By considering the overlap among various single‐cell FMs, we have selected scGPT, scBERT, and Geneformer as representative examples for our analysis. We also highlight the downstream tasks in Figure 1a. We focus on eight fine‐tuning tasks in total: (1) Batch Effect Correction; (2) Multi‐omic Data Integration; (3) Cell‐type Annotation; (4) Gene Function Prediction; (5) Perturbation Prediction; (6) Gene Network Analysis; (7) Imputation; and (8) scRNA‐seq Simulation. By compiling all the hyperparameters across different models, we present a list of factors that can affect the model performance categorized by types in Figure 1b. The definition of different hyperparameters is discussed in Appendix A. To analyze the effect of different hyperparameters, initial settings, and optimizers, we selected representative datasets for different tasks. For batch effect correction and cell‐type annotation, we selected Pancrm, HumanPBMC, Cell Lines, and MCA because they cover various data types. Pancrm is from Pancreas tissue and has five batches. HumanPBMC is from PBMC and has nine cell types. Cell Lines has two cell types, as a binary label dataset. MCA is from *Mus musculus*. For Multi‐omic Data Integration, we included all of the four datasets we used to analyze because their scales cover a large range. For Gene Function Prediction, we included all of the three datasets we used to analyze because they correspond to different sub‐tasks. For Perturbation Prediction, we included all of the three datasets we used to analyze because their scales cover a large range and are from different sources.

### 4.3 Explanations of scEval

Here, we introduce a framework known as scEval to evaluate the performance of different single‐cell FMs on various tasks. The whole pipeline is highlighted in Figure 1a. Since most of the single‐cell FMs choose to reconstruct the masked gene expression levels in the pre‐training stage, we can use an encoder–decoder structure (similar to an auto‐encoder [114]) as well as extra fine‐tuning blocks to unify their architectures. Moreover, we split the eight tasks into two types. For tasks included in sub‐task 1, we need an extra fine‐tuning block to generate the results. Cell‐type annotation and gene function prediction belong to this task type. For tasks included in sub‐task 2, we rely on the outputs of the encoder part and decoder part to generate the results. The rest of the six tasks belong to this task type. The idea of scEval is inspired by benchmarking analysis in both single‐cell area [62, 113, 115] and LLM area [116, 117]. According to Figure 1b, for each task, we collect the output of single‐cell FMs under different settings. Each FM has its specific running settings, and their default modes can be classified into fine‐tuning‐based models and zero‐shot‐learning‐based models, thus we propose a framework focusing on the outputs of different single‐cell FMs to unify the evaluation process. To analyze the factors affecting single‐cell FMs, we also adjusted the factors of different FMs and collected their results for evaluation. Moreover, scEval contains different evaluation pipelines for different tasks, and it can also be easily extended to evaluate more tasks by adding more functions.

### 4.4 Batch Effect Correction

Batch effect correction is an essential step following scRNA‐seq data pre‐processing. It primarily signifies the distribution disparity in scRNA‐seq datasets originating from the same tissue, which can be attributed to various factors [118]. The reduction of batch effects is critical not only to allow researchers to discern genuine biological signals but also to facilitate integrated analyses across different studies. The challenge of this task arises from the need to balance the removal of batch signals with the preservation of biological signals. We treat this task as a data integration problem.

For the batch effect correction, the metrics we considered here were inspired by scIB [62], including Normalized Mutual Information (NMI), Adjusted Rand Index (ARI), and Cell‐type Average Silhouette Width (cell‐type ASW) for the biological conservation score; and batch Average Silhouette Width (batch ASW), Principal Component Regression (PCR), Graph Connectivity (GC) and kBET [119] for the batch effect correction score. We compute the weighted average of these metrics to represent the final batch effect correction score. Details of these metrics can be found in Appendix D.1. Let $S_{bio}$ donate the average biological conservation metric and $S_{batch}$ donate the average batch effect correction metric as $S_{\text{batch}}$, the final model score is

$$S_{\text{final}}=0.6\cdot S_{\text{bio}}+0.4\cdot S_{\text{batch}}.$$

### 4.5 Multi‐Omic Data Integration

Multi‐omic Data Integration is a key step for multi‐omic data analysis [22]. It is akin to an advanced form of batch effect correction. If unpaired multi‐omic data are present, the objective is to map different datasets into a shared space for subsequent analysis. If paired multi‐omic data are present, the goal is to assess whether the use of multi‐omic data can contribute to learning a more comprehensive representation of the data. A significant challenge here is how to align omics at the feature level. For instance, the feature of the scRNA‐seq data is a gene, the feature of the scATAC‐seq data is a peak, and the feature of the protein data is a protein. The tokenization step can become complex given different modalities. We treat this task as a data integration problem. We used the same metrics for multi‐omic data integration as those for batch effect correction.

### 4.6 Cell‐Type Annotation

Cell‐type annotation is another key step following single‐cell data pre‐processing [120]. This step annotates each cell with its accurate cell‐type label, which can be achieved through prior knowledge [121] or computational methods [122]. These annotated cell‐type labels can provide essential biological information for further downstream analysis, such as cell‐type specific network analysis. In addition, drug response prediction [37] or single‐cell disease classification [123] can also be treated as a variation of this task. A common approach employed by single‐cell FMs in dealing with the cell‐type annotation task is to use single‐cell datasets for model training and treat the unannotated datasets as testing datasets. The challenge lies in predicting or annotating a set of cells that originate from studies different from the training datasets. Differently, SCimilarity pre‐trained the model with cell‐type labels to allow query of cell types directly for testing datasets. Moreover, the existence of cells with novel cell types (which are not included in the training datasets) further complicates the problem. We treat this task as a multi‐label classification problem. Regarding the choices of metrics for evaluation, we also computed the Pearson correlation coefficients between accuracy and weighted F1‐score for the top‐tier methods in our evaluation (including scGPT, Geneformer, and CellPLM), the coefficients are all 0.99 with a very small *p*‐value (*p*‐value <2.2e‐308). Therefore, accuracy is a suitable metric used in our comparison.

In this task, we chose datasets with batch effect in two different cases. The intra‐dataset case allows batch intersection, which means that the training and testing datasets can contain cells from the same batch. Here the total dataset was split into approximately 70% as a training dataset and the rest as a testing dataset. The inter‐dataset case is cross‐batch (cross‐data) annotation, which means that the training and testing datasets are from different sources. We consider two datasets from the same tissue in this setting. More specifically, we consider the Pancreas cross dataset from the Pancreas, and the Liver cross dataset from the Liver. The main score for evaluation here is accuracy, which is defined as

$$S_{\text{celltype}}=\frac{\#\text{corrected classified cells}}{\#\text{Total cells}}.$$

We also consider Precision, Recall, and F1 scores in the analysis for ablation test, and details can be found in Appendix D.2. Moreover, except the general comparison, we considered four datasets for the effect of different hyper‐parameters, initial settings and optimizers: Pancrm, HumanPBMC, Cell Lines, and MCA, which is from *Mus musculus* rather than *Homo sapiens*. Finally, we investigated the contribution of freezing layers for cell‐type annotation by freezing different numbers (from 0 to 11) of forward layers of scGPT.

### 4.7 Gene Function Prediction

Gene function prediction is important to identify the properties of genes across different conditions [35]. There are approximately 20 000 protein‐encoding genes for humans [124] and only some are annotated with functions. Accurate prediction of gene function can help us understand and infer the role of genes in biological systems. Here we consider three types of functions for genes. The first one is dosage‐sensitive or non‐sensitive. Some genes are dosage‐sensitive, which means that they are significant in the analysis of Copy Number Variants (CNVs) related to genetic diagnosis. The second one is Bivalent versus non‐methylated. Bivalent chromatin structure is important to identify key developmental genes in embryonic stem cells (ESCs). Therefore, identifying bivalently marked genes versus unmethylated genes is important. The third one is Bivalent versus Lys4‐only methylated. Lys4‐only‐methylated genes are also different from bivalently marked genes. We compare the model output with the true gene labels. We treat this task as a binary classification problem. Here, we used the same metrics as the cell‐type annotation task. We used a public dataset [35] considering only labeled genes in the dataset for prediction and evaluation.

### 4.8 Perturbation Prediction

Perturbation prediction [61] is a task based on gene editing and single‐cell sequencing technologies. After silencing some genes, we can obtain unperturbed and perturbed gene expression levels by sequencing, which allows us to explore the interactions between genes. A well‐known technique is Perturb‐seq [125]. In perturbation prediction, we intend to predict the gene expression level after gene editing. Here, a model may predict seen gene perturbation in the testing datasets (an easier one) or predict unseen gene perturbation in the testing datasets (a more difficult one). We treat this task as a regression problem. The metric we used here is MPC, and the details can be found in Appendix D.3. In the perturbation prediction task, we construct the paired input‐target datasets by selecting the cells with non‐control guide identity and then randomly sample cells under the control condition, and then combine them as the training and testing datasets. Our Perturb‐seq datasets are from GEARS [61], which contain cells with three conditions: control; one gene perturbation; and two genes perturbation. In the evaluation process, we combined Cases 2 and 3. We also compared the MPC across all genes to avoid bias in the gene selection process.

### 4.9 Gene Network Analysis

Gene Network Analysis is a downstream task for single‐cell datasets [126]. The objective is to infer specific gene networks (for example, Gene Regulatory Network (GRN) or Gene Co‐expression Network (GCN)) from different datasets. A GRN can assist in understanding the regulatory relationships between genes and predicted perturbation outcomes. The challenge in this task stems from the Granger causal relationship or time‐dependent correlation [127]. A GCN can be used to analyze genes with similar functions or uncover the characteristics of genes in some diseases [128]. GCN and GRN are two different tasks because correlation does not imply causal relation [129]. This limitation means that we cannot determine which genes are the “causes” of expression level changes in other genes only based on embeddings similarity or correlation. We treat this task as a network inference problem. In the gene network analysis task, we considered using the overlap between ground truth genes in certain pathways and inferred genes as one metric, and using the ratio of significant pathways related to inferred genes as the other metric. Details can be found in Appendix D.4.

### 4.10 Imputation

Imputation is a filling task related to missing data. Generally, we have two targets: 1. Perform imputation for scRNA‐seq data to reduce data noise and fill in technical zeros with biologically meaningful values [130, 131]. 2. Perform imputation for spatial transcriptomic data because of unseen or unmeasured genes [75, 76]. According to [132], current spatial imputation methods do not show strong performance across different datasets. Using single‐cell FMs, we can either use zero‐shot learning to impute the unseen genes, or fine‐tune our model based on reference scRNA‐seq with more genes to perform imputation. We treat this task as a matrix‐completion problem. Details of the metrics we used here can be found in Appendix D.5. In the imputation task, we used two public datasets from the mouse tissue to analyze the performance of single‐cell FMs. One dataset is a scRNA‐seq dataset, and another one is a spatial transcriptomic dataset. For the imputation of the scRNA‐seq dataset, we used the output of the model decoder as imputation results. To evaluate this task, we used the biology preservation score from the batch effect correction and compared it to the score from raw data. For the imputation of spatial transcriptomic data, we considered two different settings to perform imputation. The first setting uses scRNA‐seq to perform fine‐tuning and inference based on spatial transcriptomic data. The second setting uses a zero‐shot learning framework to directly perform inference based on spatial transcriptomic data. We consider using the correlation between known raw gene expression and known imputed gene expression as a metric.

### 4.11 Simulation

scRNA‐seq Simulation is a data generation task. Leveraging the generative pre‐training process of scGPT, we can generate gene expressions based on real datasets. Since a prevalent issue with scRNA‐seq data simulation is the considerable divergence between simulation datasets and real datasets [80], direct generation from real datasets is preferred.

By arranging different sequences of masking genes or altering different seeds, we can generate new simulated scRNA‐seq datasets from real ones. By modifying scGPT, we fine‐tuned the baseline model based on the reference scRNA‐seq dataset with reconstruction loss and utilized the outputs of the decoder part to generate the simulation datasets. Such simulation datasets do not have exactly the same gene expression profiles compared with the reference scRNA‐seq dataset, but they should preserve the biological variation information from the reference dataset. We utilized scGPT to simulate datasets with batch effect or without batch effect. To simulate datasets with batch effect, we removed the loss function for removing batch effect in the original pipeline. To simulate datasets without batch effect, we kept the original pipeline for the batch effect correction task but utilized the outputs of the decoder rather than the encoder. We did not incorporate extra information for implementing this function. The quality of our simulation datasets can be evaluated by comparing them with the outputs of current simulation methods. We treat this task as a data‐generation problem.

We used the same metrics as batch effect correction for evaluation. It is possible to produce diverse reconstruction outcomes from a single real dataset by varying the random seeds. This feature enables us to create simulated single‐cell datasets. Notably, these generated datasets retain the same gene sets as their input counterparts. We generate the simulation results by multiplying the output of the gene expression decoder and Bernoulli decoder. We have the flexibility to generate datasets either with or without batch effects. If we intend to produce datasets with batch effects, the gradient reverse loss function is omitted. Conversely, to generate datasets without batch effects, we either retain this function or utilize single‐batch data.

### 4.12 Model Scaling

Refs.[82, 83, 84] argue that due to the extensive number of parameters, FMs can manage specific tasks that small‐scale models cannot handle. Such an attribute refers to the scaling law. We hypothesize that single‐cell FMs may also possess this capability. To test this, we devise different scenarios similar to instances of model scaling to assess the performance of single‐cell FMs. Such scenarios include: Cross‐dataset cell‐type annotation, cross‐species cell‐type annotation, and spatial transcriptomic data analysis.

### 4.13 Ablation Tests

Given that there is no existing work investigating the significance of different loss function components of single‐cell FMs, we conducted a comprehensive analysis of the impact of various loss function components. These include the Masked Gene Expression loss (Mask Loss), Zero Log Probability Loss (Prob Loss), Gene Expression Prediction for Cell Modelling Loss (GEPC Loss), and Elastic Cell Similarity Loss (ECS Loss). We retain the task‐specific loss in the fine‐tuning process as the baseline condition (All remove). Consequently, our null hypothesis ($H_{0}$) is that the removal of the component C will not degrade the performance, while the alternative hypothesis ($H_{1}$) is that the removal of component C will worsen the model's performance. Comparing the score after eliminating a specific component to the score from the default setting allows us to determine whether to reject the null hypothesis. The test we employed here includes both the paired Students' t‐test and Wilcoxon Rank‐sum test. The details of the different loss function components are shown below.

1. Mask Loss: In both pre‐training and fine‐tuning, we mask the expression levels of some genes, denoted as $M_{\text{mask}}$, for gene expression prediction. The mask loss is motivated from this setting and works as follows:
$$\begin{array}{cc} {\tilde{x}}^{\left(i\right)} & =\mathrm{MLP}\left(h_{n}^{\left(i\right)}\right),\\ L_{\text{mask}} & =\frac{1}{\left|M_{\text{mask}\;}\right|}\sum_{j\in M_{\text{mask}\;}}\mathrm{ce}\left({\tilde{x}}_{j}^{\left(i\right)},x_{j}^{\left(i\right)}\right), \end{array}$$
  where ${\tilde{x}}^{\left(i\right)}$ represents the predicted gene expression levels for cell i, while ${\tilde{x}}_{j}^{\left(i\right)}$ represents the ground truth. $h_{n}^{\left(i\right)}$ represents the embeddings for cell i with n genes. MLP means that we use linear multi‐layer perceptron (MLP) Neural Networks as the output of single‐cell FMs under the setting of this loss function.
2. Prob Loss: Since single‐cell data can be treated as count data, we can use the Bernoulli distribution to model the occurrence of the expression in masked genes and use the maximum likelihood estimation (MLE) approach to estimate the parameters of Bernoulli distribution. Such loss function can be used to determine whether the given masked gene position carries zero expression levels or not. Prob Loss works as follows:
$$\begin{array}{cc} {\text{Prob}}_{i} & =\mathrm{MLP}\left(h_{n}^{\left(i\right)}\right),\\ {\text{Dist}}_{i} & =\mathrm{Bernoulli}({\text{Prob}}_{i}),\\ L_{\text{Prob}} & =-{\text{LogProb}}_{i}\left(x^{\left(i\right)}>0\right), \end{array}$$
  where ${\text{Prob}}_{i}$ represents the output of the model for the parameter estimation for cell i. The estimation is based on one MLP model. ${\text{Dist}}_{i}$ represents the Bernoulli distribution based on ${\text{Prob}}_{i}$. ${\text{LogProb}}_{i}$ is the log probability based on the relationship between gene expression levels of cell i and zero, which can be computed based on ${\text{Dist}}_{i}$.
3. GEPC Loss: This loss function is similar to Mask Loss, but now we predict the gene expression levels based on cell embeddings or cell representation. For cell i with gene j, we create a query vector $q_{j}$ and represent the cell based on $h^{\left(i\right)}c$. We can use the inner product between these two terms to predict gene expression levels. That is,
$$\begin{array}{cc} q_{j} & =\mathrm{MLP}\left({\mathrm{emb}}_{g}\left(t_{g}^{\left(i\right)}\right)\right),\\ {\tilde{x}}_{j}^{\left(i\right)} & =q_{j}\cdot {\mathrm{Wh}}_{c}^{\left(i\right)},\\ L_{\text{GEPC}} & =\frac{1}{\left|M_{\text{mask}\;}\right|}\sum_{j\in M_{\text{mask}\;}}\mathrm{ce}\left({\tilde{x}}_{j}^{\left(i\right)},x_{j}^{\left(i\right)}\right), \end{array}$$
  where ${\mathrm{emb}}_{g}\left(t_{g}^{\left(i\right)}\right)$ represents the embeddings of the gene token g in cell i. We also use one MLP to generate the query embeddings. The following process is similar to the steps for $L_{\text{mask}}$.
4. ECS Loss: This loss function is used to control the similarity of the embeddings of cells in the same batch, which is defined as
$$L_{\text{ECS}}=-{\left(\mathrm{CosSim}\left(h_{c}^{\left(i\right)},h_{c}^{\left(i^{\prime }\right)}\right)-\beta \right)}^{2},$$
  where CosSim represents the cosine similarity function, i and $i^{\prime }$ are the indices of the two cells. The idea of this loss function is to ensure the similarity between paired cells is higher than the predefined threshold β. Moreover, dissimilar pairs should be more dissimilar, respectively.

### 4.14 Task‐Specific Fine‐Tuning Process

For the experiments we have in the Results section, we load the pre‐training weights based on the requirement of different single‐cell FMs. The pre‐training weights we used can be found in our GitHub folder. After the fine‐tuning process, we recorded the related metrics and conducted more analysis. In the experiments for all tasks, we chose scGPT as a baseline and representative model for the following three reasons. Firstly, scGPT is an open‐source single‐cell FM with the largest datasets for pre‐training, and it is well‐defined with detailed tutorials. In addition, the architecture of scGPT is easy to adjust and includes multiple loss function components. The functions of Gene Function Prediction, Imputation, and Simulation for scGPT were designed in scEval for evaluation. The settings of hyper‐parameters for these tasks are transferred from the design for cell‐type annotation and batch effect correction tasks. Moreover, different single‐cell FMs have overlaps and unique terms in the pre‐training and fine‐tuning framework, but scGPT is the most general one. In each task, we also included task‐specific methods as comparisons. Moreover, for Geneformer, scBERT, CellPLM, and CellLM, we also evaluated their performance based on shared hyper‐parameters or optimizers with scGPT to verify if our rules found in scGPT can be extended for other single‐cell FMs.

### 4.15 Statistical Analysis

scEval is an evaluation framework to benchmark the performances of scFMs across different tasks based on various datasets. In total, we consider 8 tasks with 29 datasets. For data pre‐processing, we refer model‐specific processing. All single‐cell data are processed with Scanpy. Only Geneformer is processed by ranking the gene expression for generating cell and gene representations. For discrete data comparisons, we employed the Wilcoxon Rank‐Sum test for paired data and the Mann–Whitney U test for unpaired data. For continuous data comparison, we utilize Pearson Correlation Coefficient to measure data similarity. The significance level for all hypothesis tests was set at 0.05.

## 5 Data Availability and Reproducibility

We used the resources from the Yale High Performance Center (Yale HPC) to conduct all of the experiments. Our maximum running time for each dataset is 24 h. The version of GPU we used is NVIDIA A100 (40 GB). The random seed of all experiments is the same as the default setting of the original papers. The information of datasets and the download link can be found in Appendix G. This study includes no data deposited in external repositories. The selected cell lines are clean and contamination free. All single‐cell data are publicly available. The datasets and computer code produced in this study are available in the following databases: Adamson data: Web Resource (pert_data.load(data_name = ‘adamson’)); Cell Lines data (RRID: CVCL_0065, CVCL_0063): GitHub (https://github.com/JinmiaoChenLab/Batch‐effect‐removal‐benchmarking/tree/master); DC data: GitHub (https://github.com/JinmiaoChenLab/Batch‐effect‐removal‐benchmarking/tree/master); Dixit data: Web Resource (pert_data.load(data_name = ‘dixit’)); Heart atlas data: Web Resource (https://www.ebi.ac.uk/ena/browser/view/PRJEB39602); Human spatial data: Web Resource (http://research.libd.org/spatialLIBD/); HumanPBMC data: GitHub (https://github.com/JinmiaoChenLab/Batch‐effect‐removal‐benchmarking/tree/master); HSPC; Immune All Human data: Figshare (https://figshare.com/articles/dataset/Benchmarking_atlas‐level_data_integration_in_single‐cell_genomics_‐_integration_task_datasets_Immune_and_pancreas_/12420968); Immune atlas data: Web Resource (https://cellxgene.cziscience.com/collections/ddfad306‐714d‐4cc0‐9985‐d9072820c530); Liver cross data: Gene Expression Omnibus (https://www.ncbi.nlm.nih.gov/geo/query/acc.cgi?acc=GSE115469; https://www.ncbi.nlm.nih.gov/geo/query/acc.cgi?acc=GSE124395); Lung atlas data: Web Resource (https://cellxgene.cziscience.com/collections/6f6d381a‐7701‐4781‐935c‐db10d30de293); MB Spatial data: Python Package (squidpy.datasets.slideseqv2()); MCA data: GitHub (https://github.com/JinmiaoChenLab/Batch‐effect‐removal‐benchmarking/tree/master); MHSP data: GitHub (https://github.com/JinmiaoChenLab/Batch‐effect‐removal‐benchmarking/tree/master); Mouse Spatial data: GitHub (https://github.com/broadinstitute/Tangram/blob/master/tutorial_tangram_with_squidpy.ipynb); Mouse scRNA‐seq data: GitHub (https://github.com/broadinstitute/Tangram/blob/master/tutorial_tangram_with_squidpy.ipynb); Norman data: Web Resource (pert_data.load(data_name = ‘norman’)); PanglaoDB: Web Resource (https://panglaodb.se/view_data.php?sra=SRA553822&srs=SRS2119548). Pancrm: GitHub (https://github.com/JinmiaoChenLab/Batch‐effect‐removal‐benchmarking/tree/master); PBMC 368K: GitHub (https://github.com/AprilYuge/ResPAN); PBMC Multiomics: Web Resource (https://stuartlab.org/signac/articles/pbmc_multiomic); Kidney: Gene Expression Omnibus (https://www.ncbi.nlm.nih.gov/geo/query/acc.cgi?acc=GSE117498); HSPC: Gene Expression Omnibus (https://www.ncbi.nlm.nih.gov/geo/query/acc.cgi?acc=GSE151302); Endo: Web Resource (https://github.com/qinzhu/VisCello.eht); Pancreas cross: Web Resource (https://www.nature.com/articles/s41467‐023‐35923‐4#data‐availability); Liver cross: Web Resource (https://github.com/SydneyBioX/scClassify).

The codes of **scEval** can be found in https://github.com/HelloWorldLTY/scEval with MIT license.

## Author Contributions

T.L. designed the study with H.Z. and Y.W. T.L. ran the experiments with K.L. H.L. and T.L. designed the website. T.L., K.L. and H.Z. wrote the manuscript. H.Z. supervised this work.

## Conflicts of Interest

The authors declare no conflicts of interest.

## Supporting information


**Supporting File:**supinfo/advs74604‐sup‐0001‐SuppFile.zip.

## Data Availability

We used the resources from the Yale High Performance Center (Yale HPC) to conduct all of the experiments. Our maximum running time for each dataset is 24 hours. The version of GPU we used is NVIDIA A100 (40 GB). The random seed of all experiments is the same as the default setting of the original papers. The information of datasets and the download link can be found in Appendix G. This study includes no data deposited in external repositories. The datasets and computer code produced in this study are available in the following databases in the manuscript.
