# Supplementary material for: Evaluating the Utilities of Foundation Models in Single‐Cell Data Analysis
Source: Adv Sci (Weinh). 2026 Mar 23;13(27):e14490. doi: 10.1002/advs.202514490 (PMC13170260; doi:10.1002/advs.202514490)
Supplement: Supplementary file 1 — Supporting File:supinfo/advs74604‐sup‐0001‐SuppFile.zip. [file ADVS-13-e14490-s001.zip › advs74604-sup-0008-Figures-and-Tables.pdf]

A Hyper-parameters

We first define different hyper-parameters used for hyper-parameters analysis.

1. Dropout: The dropout rate is the probability of making the neural unit inactive or closed. If a unit is closed, it will not participate in the training process but will take part in the inference process. The design of dropout is to avoid or reduce overfitting. The scope is [0, 0.1, 0.2, 0.3, 0.5].

2. Loss weight: Changing the weights of different loss function components is a well-known approach to increasing the performance of neural networks. Here the loss weight means the weight of the task-specific loss component. For example, for the Batch Effect Correction task, such weight represents the loss weight for the loss of the gradient reverse network. The scope is [1, 5, 10, 50, 100].

3. Bins: To reduce the batch effect during the pre-training or fine-tuning process in the pre-processing step, the gene expression is divided into different intervals and the endpoint values of the intervals are used to replace the original gene expression. The intervals here are the bins. Formally, the binned expression value $x_j^{(i)}$ for cell i is:

$$x_j^{(i)} = \begin{cases} k, & \text{if } x_j^{(i)} > 0 \text{ and } x_j^{(i)} \in [b_k, b_{k+1}], \\ 0, & \text{if } x_j^{(i)} = 0, \end{cases}$$

where $k \in \{1, 2, ..., B\}$ and B represents the number of bins. The scope is [51, 101, 151, 201, 501, 1001].

4. Mask ratio: In the pre-training and fine-tuning process, we choose to mask a fixed ratio of gene expression levels and try to use our decoder to reconstruct the expression levels of the masked genes. Such a ratio is defined as a mask ratio. The scope is [0.1, 0.3, 0.4, 0.5, 0.7, 0.9].

5. Epoch: Epoch represents the number of steps we use to train our model based on the whole training dataset. The scope is [1, 5, 10].

6. Learning rate: Learning rate is designed for the optimizer, it represents the step size of gradient descent. The scope is [5e-5, 1e-4, 1e-3, 1e-2].

7. ECS threshold: The threshold β in the ECS Loss represents the upper bound for the similarity between paired cells. The scope is [0, 0.2, 0.4, 0.6, 0.8, 1.0].

Details of the settings of hyper-parameters we used in the evaluation for all of the methods are summarized in Supplementary file 4.

B Initial Settings

In this section, we delineate the initial settings for Batch Effect Correction, Multi-omic Data Integration, Cell-type Annotation, and Perturbation Prediction tasks.

1. Zero-shot learning: This refers to using our pre-trained model without any further training for unseen datasets on specific tasks. This setting aids in validating the significance of pre-training.

2. No pre-training: This setting entails starting the fine-tuning process without any pre-trained weights, allowing us to train a *de novo* single-cell FM. This configuration helps ascertain the importance of pre-training.

3. Cross-entropy (default setting): Here, we utilize the cross-entropy loss function for the reconstruction of gene expression levels, which is the default setting in scGPT.

4. Mean Squared Error (MSE) loss: In this setting, we employ the MSE loss function for the reconstruction of gene expression levels.

5. Freeze except encoder: This setting involves freezing the weights of the encoder component during the fine-tuning process.

6. Freeze except decoder: Here, we freeze the weights of the decoder section during the fine-tuning process.

7. Training by reward network: The successful application of Human-feedback Reinforcement Learning (HFRL) to the training process for FMs has been established [133], and we borrow ideas from [93, 134]. We consider cell types as the human label for different cells, akin to the labels of sentences in the NLP area. A reward network is employed to predict the cell types of the provided cells and minimize the loss in this process. This procedure can also be integrated into the pre-training process or prompt-based learning framework. This approach can also be regarded as joint fine-tuning for Batch Effect Correction and Cell-type Annotation.

The results from this analysis provide valuable insights into how to configure scGPT most effectively for this specific task.

C For developers: How to justify a good foundation model for single-cell analysis?

By summarizing our experimental results, here we list essential factors and criteria for selecting benchmarking datasets for developers of single-cell foundation models.

Regarding important factors, we recommend that developers and researchers consider the following:

- Including basic tasks to demonstrate its functionality. Current single-cell foundation models work well for basic tasks, including cell-type annotation, gene function prediction, and perturbation prediction. These tasks can be utilized as a starting point for developing foundation models.
- Improving model performances under challenging tasks. We demonstrated that single-cell foundation models could have performed better in batch effect correction, imputation, and simulation, which should be considered as hard but important tasks for resolving with new foundation models. For simulation, it is important to investigate the generator used for simulating the gene expression profiles to preserve the correct gene-gene correlation.
- Demonstrating the biological discoveries from foundation models. Training a foundation model has a high cost, and thus the developers may consider novel tasks in single-cell analysis and compare the foundation model with simple baselines and well-defined metrics. If necessary, developers are encouraged to collaborate with biologists for experimental verification.
- Exploring the model capacity for both zero-shot learning and fine-tuning. The ultimate task of a single-cell base model is to be able to understand the underlying biological system on a cellular basis, so it is important to measure the zero-shot

learning capacity after model training. We understand that there still exits performance gap between zero-shot learning capacity and fine-tuning capacity for the same task, but we encourage the developers to explore effective approaches to enclose the gap or recommend better designs for the given task.

- Incorporating multimodel data in the training stage to increase capacity. The information from transcriptomic data is limited, and thus, introducing data from other domains might enhance the ability of single-cell foundation models to understand biology.
- Aligning single-cell foundation models with principles from other areas. There are some abilities of Large Language Models (for example, scaling law in various tasks and content generation) that are not observed in single-cell foundation models, so the developers may seek explanations or methods to bridge the gap.
- Ensuring trust and robustness. We demonstrated that single-cell foundation models suffer from unstable problems in specific tasks, and foundation models always risk being attacked. Therefore, developing a safe and robust model should be taken into consideration.
- Enhancing accessibility by open-source and user-friendly designs. We encourage open-science research, and thus, developing fully open-source models is recommended. Moreover, considering the hardware limitation of biologists, developing a model with fewer resources for deployment is encouraged.

Regarding dataset selection, we recommend developers and researchers consider the following:

- Selecting suitable datasets for pre-training is important. Many tasks in single-cell analysis can be handled by methods developed based on cell embeddings or gene embeddings without further fine-tuning, and thus, learning a good representation of cells and genes in the pre-training stage is important. The developers should consider this point in the data collection stage and the training/validation/testing separation stage.
- Evaluating the model with commonly used datasets. Considering the faster development speed in the single-cell analysis area, many techniques are outdated and have limited contribution even after integration. Therefore, evaluating the performances of single-cell foundation models in commonly used datasets (like scRNA-seq from 10X) is encouraged. Moreover, methods with functions for handling spatial transcriptomics should also consider datasets in different resolutions.
- Considering the diversity of datasets. Multiple biological factors can affect the variation of single-cell datasets, including tissue type, disease state, cell type, perturbation, etc. Ensuring and demonstrating that the model can learn information from various data sources are important.
- Avoiding data leakage. It is essential to ensure no intersection between training and testing datasets, and an improper split might inflate model performance in the testing stage.

D Metrics Information

Here we describe details about the metrics we used in the evaluation process for different tasks.

D.1 Batch Effect Correction, Multi-omic Data Integration and Simulation

1. Normalized Mutual Information (NMI): NMI is a score to evaluate the performance of biological information preservation. We calculate this score based on computing the mutual information between the optimal Leiden clusters and the known cell type labels and then take the normalization. Therefore, NMI $\in (0,1)$ and higher NMI means better performance.

2. Adjusted Rand Index (ARI): ARI is a score to evaluate the performance of biological information preservation. ARI can measure the agreement between optimal Louvain clusters and cell type labels. ARI $\in (0,1)$ and higher ARI means better performance.

3. Average Silhouette Width (ASW): Here we have cell type ASW (ASW_{cell}) and batch ASW (ASW_{batch}). For one cell point, ASW calculates the ratio between the inner cluster distance and the intra cluster distance for this cell. Therefore, higher ASW_{cell} means better biological information preservation and lower ASW_{batch} means better batch effect correction. To make them consistent, for ASW_{cell}, we take the normalization, that is:

$$ASW_{cell} = \frac{ASW_{cell}^{raw} + 1}{2}.$$

Similarly, for ASW_{batch}, we take the inverse value of the normalization, that is:

$$ASW_{batch} = 1 - \frac{ASW_{batch}^{raw} + 1}{2}.$$

Both of metrics are in (0,1), and a higher score means better model performance. For multi-omic data integration, all the metrics are the same.

4. Graph Connectivity (GC): GC means the connectivity of cells in different cell types. If the batch effect is substantially removed, the connectivity of cells of the same cell type from different batches will have a higher connectivity score based on the k-NN neighbor graph. Therefore, we can calculate the GC score for each cell type and take the average. GC score is in (0,1) and higher means better batch effect correction performance.

5. Principal Component Regression (PCR): PCR is a metric to evaluate the performance of batch effect correction. We calculate the R^2 for a linear regression of the covariate of interest onto each principal component. The variance contribution of the batch effect for all the PCs is based on the sum of the product between the variance of each PC and the R^2 of each PC across all the PCs. Therefore, the score can be represented as:

$$PCR = \sum_{i=1}^{G} \operatorname{Var}(C \mid \mathrm{PC}_i) \times R^2(\mathrm{PC}_i \mid B),$$

where G denotes the number of PCs and B denotes the batch information. PCR is in (0,1) and a higher score means better performance.

6. kBET: the kBET algorithm is used to determine if the label composition of the k-nearest-neighbors of a cell is similar to the expected label composition. For the batch label mixture of cells in the same cell types, the proportion of cells from different batches for the neighbors of one cell should match the global level distribution. The kBET score is in (0,1) and higher means better batch effect correction performance.

We computed the S_{bio} based on the average value of NMI, ARI, and ASW_{cell}. The clustering scores are computed based on searching the leiden resolution to achieve the best performances based on scIB. We computed the S_{batch} based on the average value of ASW_{batch}, GC, PCR, and kBET. $S_{final} = 0.6S_{bio} + 0.4S_{batch}$. We also investigated the choices of weights for computing the final score. scIB chose to assign a higher weight for S_{bio} based on the prior information that biological variation plays a more important role in the analysis. To compare the difference between the default setting of weights and other settings of weights, we designed two methods for computing the final rank of different methods across different datasets.

The first method we tried is to adjust the weights of S_{bio} from 0.5 to 0.9, and then compute the metrics for each method under each dataset based on different weights. For each weight, we could compute the final score and ranked these methods by different datasets. Thus, we finally had five different rank tables. Now we averaged the ranks of different methods across datasets to get the final average rank of each method, and such value was used for our final evaluation. The result is shown in Figures S 46 (a).

The second method is the same as our original method. The result is shown in Figures S 46 (b).

Therefore, we only need to check the difference between the first method and the second method to see whether we need to optimize the weights for S_{bio} and S_{batch} or we can treat them based on our prior. We compared the average rank results from Figures S 46, and we found that the top three methods are exactly the same under different methods. Moreover, only the average rank of two different methods was affected by adjusting methods for computing the ranks, and their actual average rank is also very close. Furthermore, we computed the Spearman correlation coefficient between these two rank results, and we found that the correlation is 0.98 with a p-value smaller than 1e-55. Therefore, the results of these two methods are in strong correlation. It means that we do not need to adjust weights for different scores and the original choice of scIB and our manuscript is robust enough to evaluate the performance of different methods in the batch effect correction task.

For specific datasets with trajectory information, we also include the trajectory score to evaluate the conservation of trajectory information after batch effect correction. According to [62], to compute this score, we first calculate the diffusion pseudotime with a known initial cluster and trajectory relationship, and then we compute the Spearman correlation coefficient s_{sp} between the pseudotime values before and after integration. Therefore, the trajectory score is defined as:

$$Traj_{score} = \frac{s_{sp} + 1}{2}.$$

Higher $Traj_{score}$ means better trajectory conservation performance.

These metrics are also used for evaluating Multi-omic Data Integration. For simulation analysis, we only evaluated the biological information preservation for the simulation output with batch effect setting.

We also considered qualitative assessment. By visualizing the cell embeddings of these tasks using UMAPs, we could evaluate the performance of different methods for removing the batch effect and preserving the biological variation. A dataset without large batch effect should have a pattern that cells from the same cell type but different batches in the same or similar clusters.

D.2 Cell-type Annotation and Gene Function Prediction

1. Accuracy: We calculate the accuracy based on the ratio between the total number of cells (or genes) being classified correctly and the total number of cells (or genes).

2. Precision, Recall, and F1: For classification results, we calculate true positives (tp), false positives (fp), true negative (tn), and false negatives (fn). Precision, Recall, and F1 score are calculated as follows:

$$\begin{aligned} Precision &= \frac{tp}{tp + fp}, \\ Recall &= \frac{tp}{tp + fn}, \\ F1 &= \frac{2 \cdot Precision \cdot Recall}{Precision + Recall}. \end{aligned}$$

Note that precision, recall, and F1 are calculated based on the weighted average, where the weight of each cell type is determined by the cell number.

For the Gene Function Prediction task, all of the metrics are the same but for genes rather than cells.

D.3 Perturbation Prediction

We use the Mean Pearson Correlation (MPC) and Mean Squared Errors (MSE) in the validation dataset to evaluate the performance of scGPT across different datasets.

Considering we have n genes in total. The Pearson correlation for the predicted gene expression g_i' of gene i across all the cells and the ground truth gene expression g_i of gene i across all the cells is:

$$\begin{aligned} S_{\mathrm{PC}i} &= \frac{COV(g_i', g_i)}{\sigma_{g_i'} \cdot \sigma_{g_i}}, \\ S_{\mathrm{MPC}} &= \frac{\sum_i^n S_{\mathrm{PC}i}}{n}, \end{aligned}$$

where $COV(,)$ is used to compute the covariance of two vectors, and σ is the standard deviation of the given vector. Higher S_{MPC} means better performance.

Similarly, we have MSE defined as:

$$S_{\text{MSE}} = \frac{\sum_i^n \sum_j^m (g_{ij} - g'_{ij})^2}{nm},$$

where m represents number of cells. Lower S_{MSE} means better performance.

D.4 Gene Network Analysis

Here we extract the genes related to Human Immunology pathway. Moreover, we extract genes that have significant correlations with CD3- genes from our gene embedding dataset. We then measure the Jaccard similarity for these two gene sets G_{path} and G_{net} as follows,

$$Jaccard = \frac{|G_{path} \cap G_{net}|}{|G_{path} \cup G_{net}|},$$

where G_{path} represents the genes contained in the prior known pathway, and G_{net} represents the genes contained in the GCN generated by single-cell FMs. The Jaccard similarity measures the similarity between two gene sets to evaluate the quality of GCN inference proposed by single-cell FMs. Higher Jaccard similarity means better performance.

Moreover, we performed the gene enrichment analysis for genes from G_{net}. We extracted the pathways enriched in these genes and calculated the total number of significant pathways, after which we divided this number by the number of all pathways to get the final ratio. Higher ratio means better performance. The p-value threshold was adjusted based on Bonferroni correction.

We also considered qualitative assessment. By visualizing the gene embeddings of these tasks using UMAPs, we could evaluate the performance of different methods for generating meaningful embeddings by checking the co-embedded conditions for marker genes with similar expression patterns by cell type. Such marker genes from the same cell type should be located in the same cluster, while the difference of marker genes from different cell types should be preserved, in a space from good gene emebddings.

D.5 Imputation

1. Average bio: The average bio score is calculated based on cell-type NMI and ARI scores using the latent space.

2. Correlation: The correlation score is calculated based on the average correlation between the raw data and imputed data for known common genes. The justification of this metric is based on the assumption that the target of spatial imputation is to predict the expression of missing or unmeasured genes while keeping the distribution of known gene expression levels. We use Pearson correlation here.

3. Significance proportion: The significance proportion corresponds to the proportion of genes with their p-value of correlation smaller than a specific threshold, which is 0.005 in our evaluation. The proportion can be used as a metric to evaluate the similarity between two paired data.

E Evaluation Methods

Here we describe the details of the implementation of the methods discussed in this manuscript.

Single-cell FMs include:

- tGPT: tGPT is a single-cell FM based on the GPT-2 [135] structure. It utilized large-scale scRNA-seq datasets for pre-training and set the pre-training task as predicting gene expression rankings. The downstream applications of tGPT follow the zero-shot learning framework and include clustering, batch effect correction, and bulk RNA-seq analysis. We evaluate the performance of tGPT for Batch Effect Correction, Cell-type Annotation, and Perturbation Prediction.
- scBERT: scBERT is a pre-training-based single-cell FM focusing on cell-type prediction. It is based on Performer [136] with gene embeddings initialized by Gene2vec [60]. It has six self-attention blocks. The default fine-tuning process of scBERT on downstream datasets is freezing the penultimate layer. scBERT is considered for Cell-type Annotation.
- Geneformer: Geneformer is a single-cell FM using transfer learning to predict cell types and gene functions. The Geneformer tokenization step is done based on ranking gene expression values in single cells following scaling across the whole training dataset. Cells are represented as token strings with genes rankings as tokens. We evaluate the performance of Geneformer for Batch Effect Correction, Cell-type Annotation, Gene Function Prediction, Perturbation Prediction, and Gene Network Analysis.

  Geneformer is used for the Cell-type Annotation task and the Gene Function Prediction task. Both tasks were performed by fine-tuning on the basis of the published pre-trained Geneformer network. The default hyper-parameters were used for Geneformer fine-tuning. Prior to tokenization, gene names in all datasets were converted to ENSEMBL IDs using python packages *mygene* [137] and *pyensembl* [138], and most of unmatched genes are non-coding RNAs.

  For consistency with other benchmarked FMs, Geneformer's workflow in Gene Function Prediction and non-cross Cell-type Annotation was altered to split all labeled genes into the training and the testing datasets using the same manners mentioned in our Methods section, instead of using the cross-validation evaluation method provided by the authors.
- CellLM: CellLM is a single-cell FM using three different pre-training strategies. The pre-training loss function includes: 1. masked gene expression level reconstruction; 2. cell condition discrimination; and 3. self-supervised contrastive learning. Moreover, it incorporated protein-protein interaction networks as prior information during the pre-training process. The downstream tasks of CellLM are all related to Cell-type Annotation.

  Here we modified CellLM so that it can perform cross-dataset cell-type annotation, by separating the training and the testing datasets.
- scFoundation: scFoundation employs a pre-training methodology similar to BERT and introduces Bayesian down-sampling as a data pre-processing step. The input data of scFoundation also contain target total counts and input total counts as extra

information. The downstream tasks of scFoundation include clustering (a function of cell embeddings across all models), drug response prediction (belong to Cell-type Annotation) and Perturbation Prediction. We evaluate the performance of scFoundation for Batch Effect Correction, Cell-type Annotation, Perturbation Prediction, and Gene Network Analysis.

- SCimilarity: SCimilarity declares that it serves as a foundation model for new data querying or searching based on the cell embeddings generated from known large-scale scRNA-seq datasets. It pre-trains a MLP rather than transformer-based models. The downstream tasks of SCimilarity include Batch Effect Correction and Cell-type Annotation. We evaluate the performance of SCimilarity for Batch Effect Correction, Cell-type Annotation, and Perturbation Prediction.
- CellPLM: CellPLM utilizes cells as tokens and also pre-trains a transformer-based model based on both scRNA-seq datasets and spatial transcriptomic datasets. CellPLM also constructs the latent space based on Gaussian Mixture priors and their decoder part accepts the latent variables sampled from this latent distribution. We evaluate the performance of CellPLM for Batch Effect Correction, Cell-type Annotation, and Imputation. The codes for Perturbation Prediction based on CellPLM were not evaluated because they did not release codes for performing this task.
- UCE: UCE utilizes genes as tokens and sorts the gene expressions by the genomic location of genes. UCE pre-trains a transformer-based model augmented by gene tokens from a large protein language model (PLM) based on mega-scale atlas scRNA-seq datasets. UCE is capable of Batch Effect Correction, Cell-type Annotation, and Gene Expression Prediction. We evaluate the performance of UCE for Batch Effect Correction, Cell-type Annotation, and Perturbation Prediction because they did not release codes for performing other tasks.
- GeneCompass: GeneCompass pre-trains a transformer-based model with external knowledge embeddings including Gene Regulatory Network (GRN), Promoter, Gene family, and Gene co-expression. It has an expression decoder and gene ID encoder to perform self-supervised learning. GeneCompass can perform tasks including Cell-type Annotation, Perturbation Prediction, Dose Response Prediction, and GRN Inference. We did not evaluate the performance of GeneCompass because it is totally closed-source with no pre-training weights and instructions.

Task-specific methods are SOTA models in their areas based on their description. They include:

- ResPAN: ResPAN is a batch effect correction tool based on Generative Adversarial Network (GAN) [139, 140]. The high-level idea of ResPAN is based on the distribution alignment or domain adaption across data from different batches. Such requirement can be treated as optimal transport, which can be accomplished by training a GAN. ResPAN is used for evaluating the batch effect correction task.
- Harmony: Harmony is a batch effect correction tool starting from PCs based on self-supervised learning and vector correction. Harmony sets up the initial clustering labels for uncorrected data. For cells with the same cluster labels, Harmony performs correction for cell embeddings of each cluster, and then repeats the clustering process until convergence. Harmony is used for evaluating the Batch Effect Correction task.

- scVI (scANVI): scVI is a batch effect correction tool based on variational inference and variational auto-encoder [141]. scVI encodes the gene expression data with batch information using a neural network and sets the output of the network as parameters for a distribution. Based on such distribution of the latent space, scVI can correct the batch effect in the latent space as well as the original space, as long as we consider the output of the decoder model. scANVI is a version for label transfer.
- scJoint: scJoint is a multi-omic data integration tool based on an auto-encoder structure. It contains three steps. In the first step, scJoint performs semi-supervised transfer learning. In the second step, scJoint performs label transfer based on the kNN classification for the joint cell embeddings. In the last step, scJoint starts joint training for the labeled data to optimize the cell embeddings. scJoint is used for evaluating the Multi-omic Data Integration task.
- scGLUE: scGLUE (GLUE) is a multi-omic data integration tool based on VAE and Graph Neural Network (GNN). It contains two parts. For the feature-encoding part, scGLUE utilizes a Knowledge-based guidance graph with GNN to generate feature embeddings. For the cell-encoder part, scGLUE utilizes VAE to generate cell embeddings. The decoder process is constructed by performing inner-product based on the cell embeddings and feature embeddings. scGLUE is used for evaluating the Multi-omic Data Integration task.
- *Vanilla* NNs: This neural network contains three MLP layers with batch normalization and uses Mish [142] as the activation function. *Vanilla* NNs are used for evaluating the Cell-type Annotation task and the Gene Function Prediction task. We trained *Vanilla* NNs based on different input datasets. The learning rate is set as 1e-4, the optimizer is Adam, and the epoch is set as 10. We tuned the best model by splitting training datasets and testing datasets for different tasks.
- TOSICA: TOSICA is a deep learning-based method for one-stop cell type annotation. TOSICA is designed with the self-attention multi-heads transformer without pre-training. It also provides interpretation for researchers about the attention embeddings and uses attention embeddings to perform biological analysis. TOSICA is used for evaluating the Cell-type Annotation task.
- SVM_{rej}: SVM_{rej} is a machine learning based classifier for cell-type annotation. This method is designed based on support vector machine with calibration settings. This method was evaluated in [59] and was a top-tier method. SVM_{rej} is used for evaluating the Cell-type Annotation task.
- Gene2vec: Gene2vec is a tool to generate gene embeddings based on Word2vec [143]. It trains a Word2vec model to predict the gene-gene interaction using known datasets, and generates gene embeddings of these genes after training. Gene2vec is used for evaluating the Gene Function Prediction task.
- GEARS: GEARS is a tool for single and multi-gene perturbation prediction based on single-cell RNA sequencing datasets. It combines gene-gene interaction network as prior information and uses a cross-gene neural network with a graph neural network to predict gene expression after perturbation.
- Tangram: Tangram is a toolbox for spatial transcriptomic data analysis based on neural networks. The key idea behind Tangram is using neural networks to find a good mapping function from single-cell data space to spatial data space. After the

mapping process, by integrating the information from the single-cell level and spatial level, it can perform several downstream tasks, including data imputation, cell-type deconvolution, and others. Tangram is used for evaluating the Imputation task.

- scDesign3: scDesign3 is a model based on Copula distribution [144] to generate different single-cell datasets. Such datasets can be multimodal. Moreover, based on the input parameters and requirements of scDesign3, it can also generate datasets with specific conditions, including batch effect, cell conditions, and the stages of cell differentiation. The data generation of scDesign3 is based on real datasets. scDesign3 is used for evaluating the Simulation task.
- Splatter: Splatter is a model based on joint probabilistic inference. It models scRNA-seq data by estimating certain parameters from known scRNA-seq data. Then Splatter combines the estimated parameters with additional parameters to simulate datasets under different conditions. All the distributions used in Splatter are the known distributions. Splatter is used for evaluating the Simulation task.
- SATURN: SATURN is a model designed for learning cross-species cell embeddings and integrating datasets from different species. It has two stages, including a pre-training stage as well as a fine-tuning stage based on supervised contrastive learning by using the matched cell-type labels. To make a fair comparison for the cross-species cell-type annotation analysis, we utilize the cell embeddings of SATURN from the pre-training stage to annotate the cell types for datasets from different species.
- Novae: Novae is a model pre-trained with large-scale spatial transcriptomics data. It is based on the optimal transport and contrastive learning to learn the spot representations in a joint space. We utilize Novae in evaluating the batch effect correction performance for spatial transcriptomics data.
- scWGCNA: scWGCNA is a tool for estimating gene-gene co-expression networks in scRNA-seq data. It incoporates the pipeline from WGCNA and aggregates gene expression profiles from cells into pseudobulk setting by selecting neighbors as the first step.

F Model Architecture

The model architecture of scGPT, scBERT, CellLM, Geneformer, tGPT, SCimilarity, UCE, CellPLM and scFoundation are provided in the Supplementary file 5.

G Data availability

No new sequencing data were generated for this current study. Supplementary file 6 provides the sources and download links for the datasets used in each task. These datasets come from the following papers:

Batch Effect Correction: [49, 62, 115, 145–147].
Cell-type Annotation: [23, 49, 62, 115, 148, 149].
Gene Function Prediction: [46].
Multi-omic Data Integration: [150–153].
Perturbation Prediction: [61].
Imputation: [75].
Gene Network Analysis: [62, 115].

Simulation Analysis: [115].

Model Scaling: [115, 148, 154].

H Running statistics

We summarized the running time, peak CPU memory usage and peak GPU memory usage, in Supplementary file 7.

I Supplementary Figures

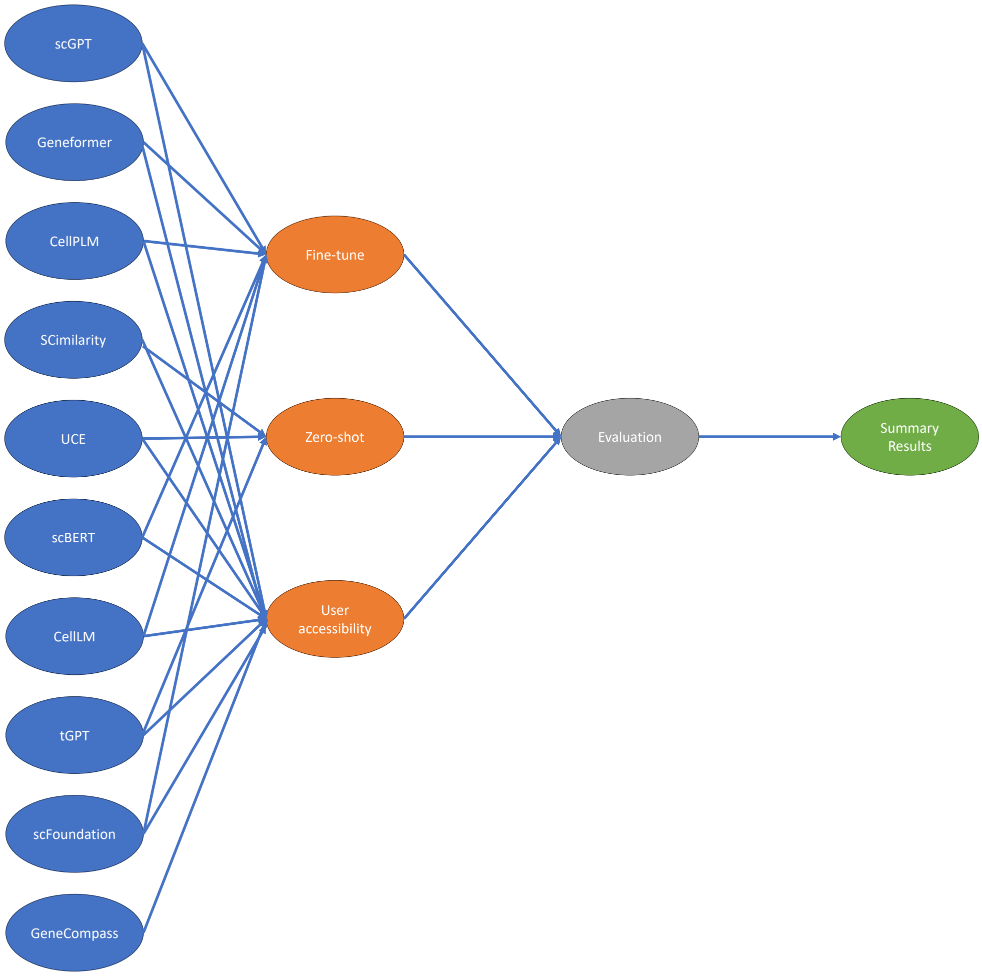

Figure S 1: Workflow of our evaluation. Here we considered ten single-cell FMs and generated their outputs based on their default usage settings. We also consider the evaluation of user accessibility for both open-source and closed-source tools. We then evaluated the outputs with task-specific metrics. Finally, we summarized our discoveries in this manuscript.

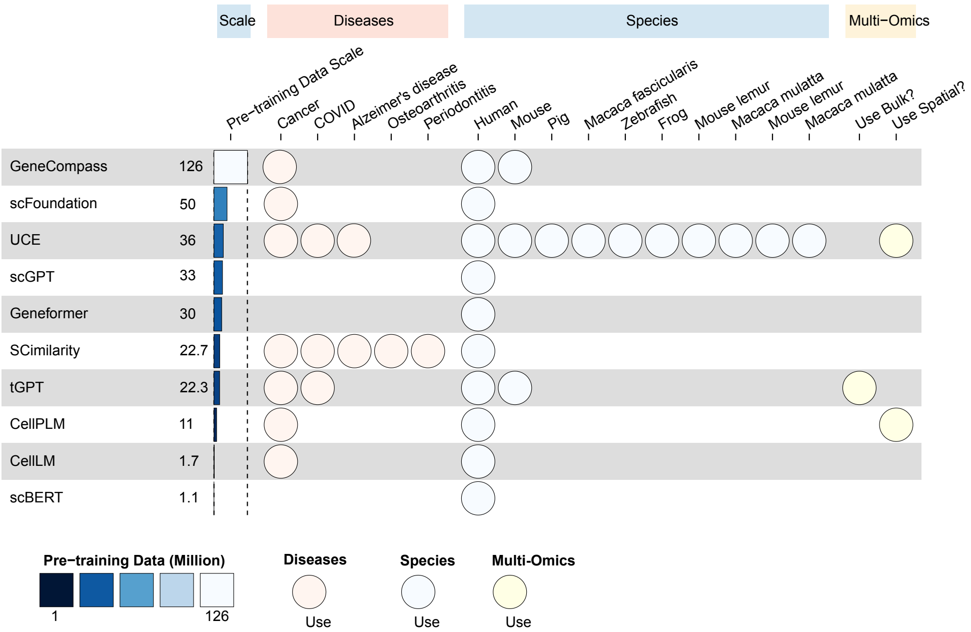

Figure S 2: Statistics of pre-training datasets for different single-cell FMs. Here we consider summarizing the scale of pre-training datasets, the overlap for major diseases in the pre-training datasets, the overlap for major species in the pre-training datasets, and the overlap of multi-omics in the pre-training datasets, across all the methods. The scale of pre-training dataset is a continuous variable, whether other statistics are recorded based on a binary variable.

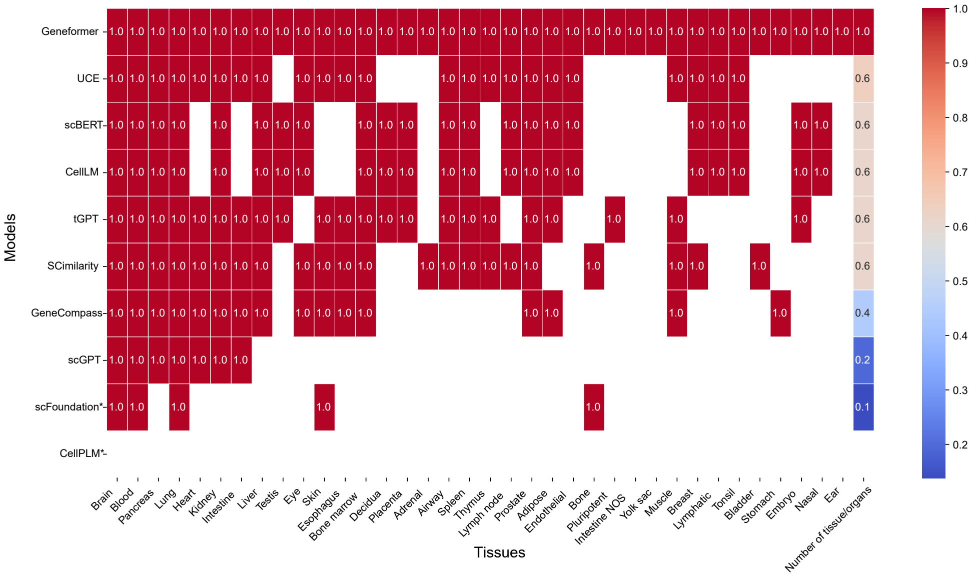

Figure S 3: The overlap of major human tissues or organs across different single-cell FMs' pre-training datasets. Here we choose tissues or organs included by Geneformer as a baseline because it is published and it includes most of the major information for the cells from human. *: For scFoundation, we only record the major tissues or organs based on its manuscript since we did not find the data sources description from its manuscript and supplementary files. For CellPLM, we did not record the major tissues or organs since we did not find the data source description from its manuscript and supplementary files.

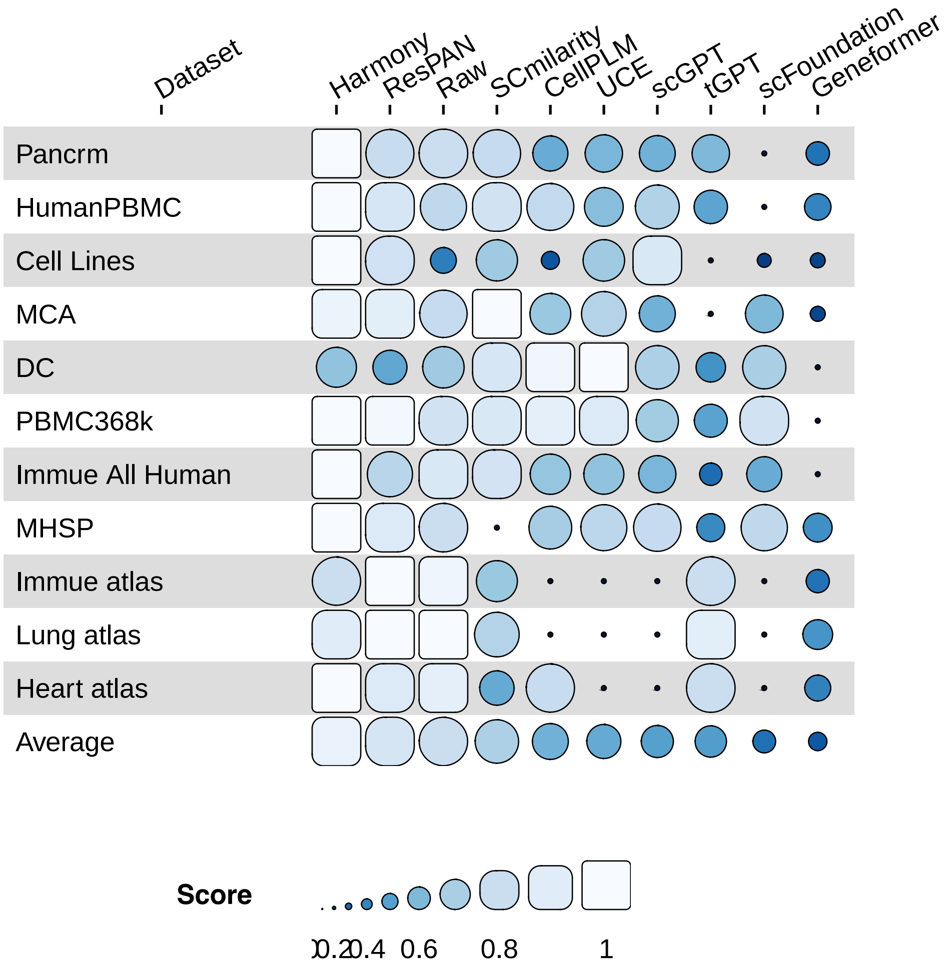

Figure S 4: S_{final} across different scFMs based on the zero-shot setting.

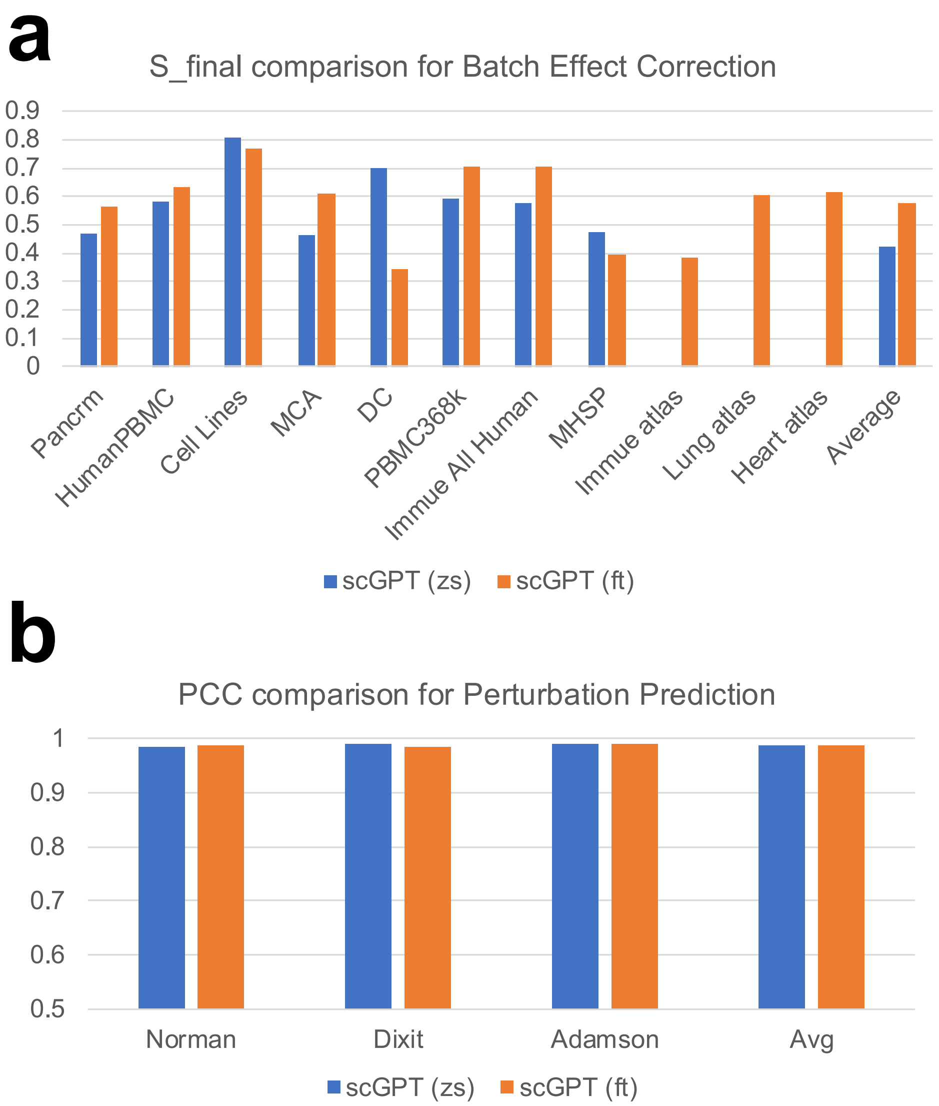

Figure S 5: Compassion of zero-shot setting and fine-tuning setting of scGPT. (a) Comparison based on S_{final} for batch effect correction. (b) Comparison based on PCC for perturbation prediction.

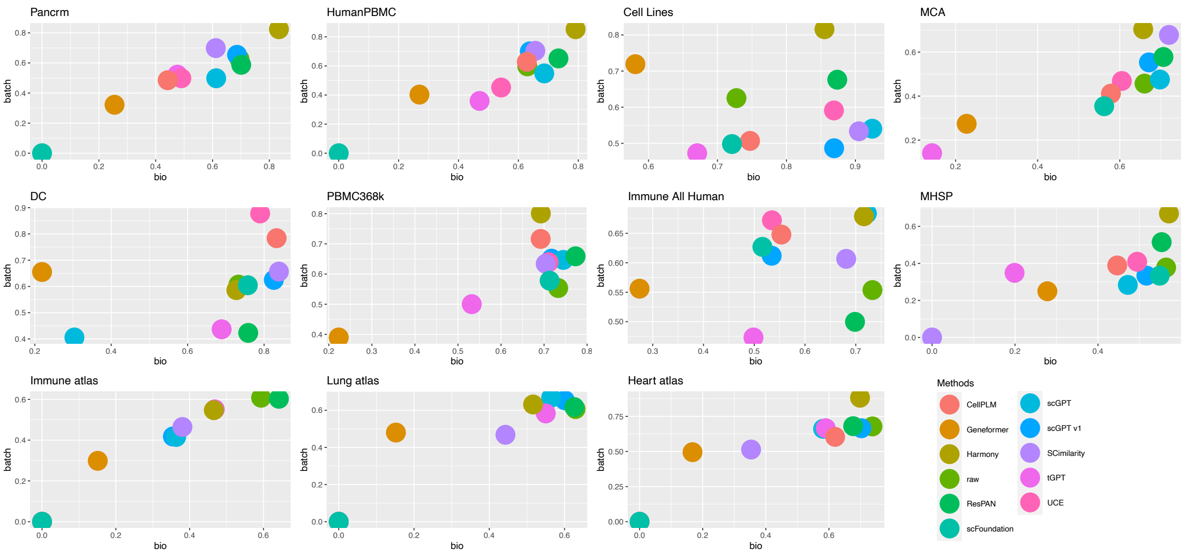

Figure S 6: Visualization of batch effect removal score and biology variation conservation score in the evaluation of batch effect correction task for each dataset.

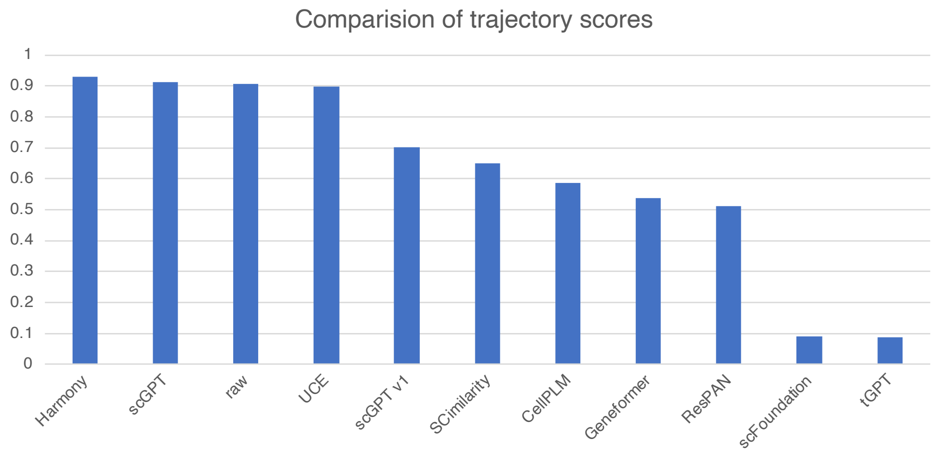

Figure S 7: Comparisons for different methods on preserving the trajectory information based on the trajectory score.

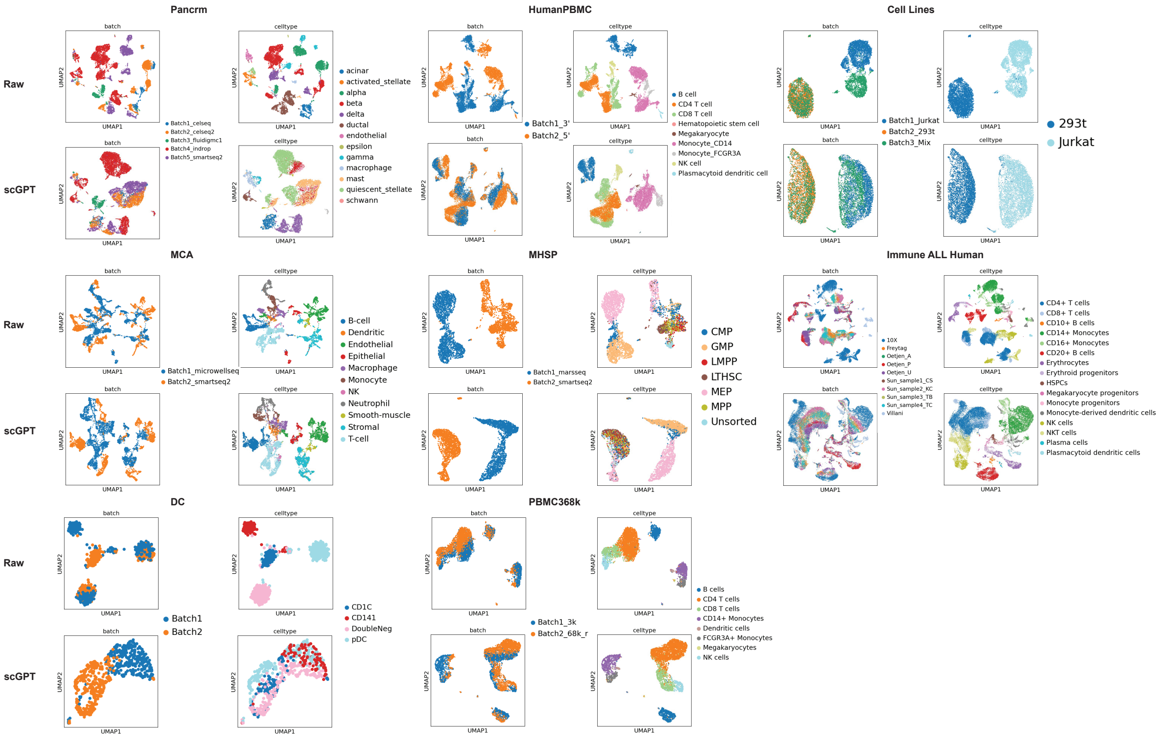

Figure S 8: UMAPs for raw data and embeddings of scGPT after batch effect correction.

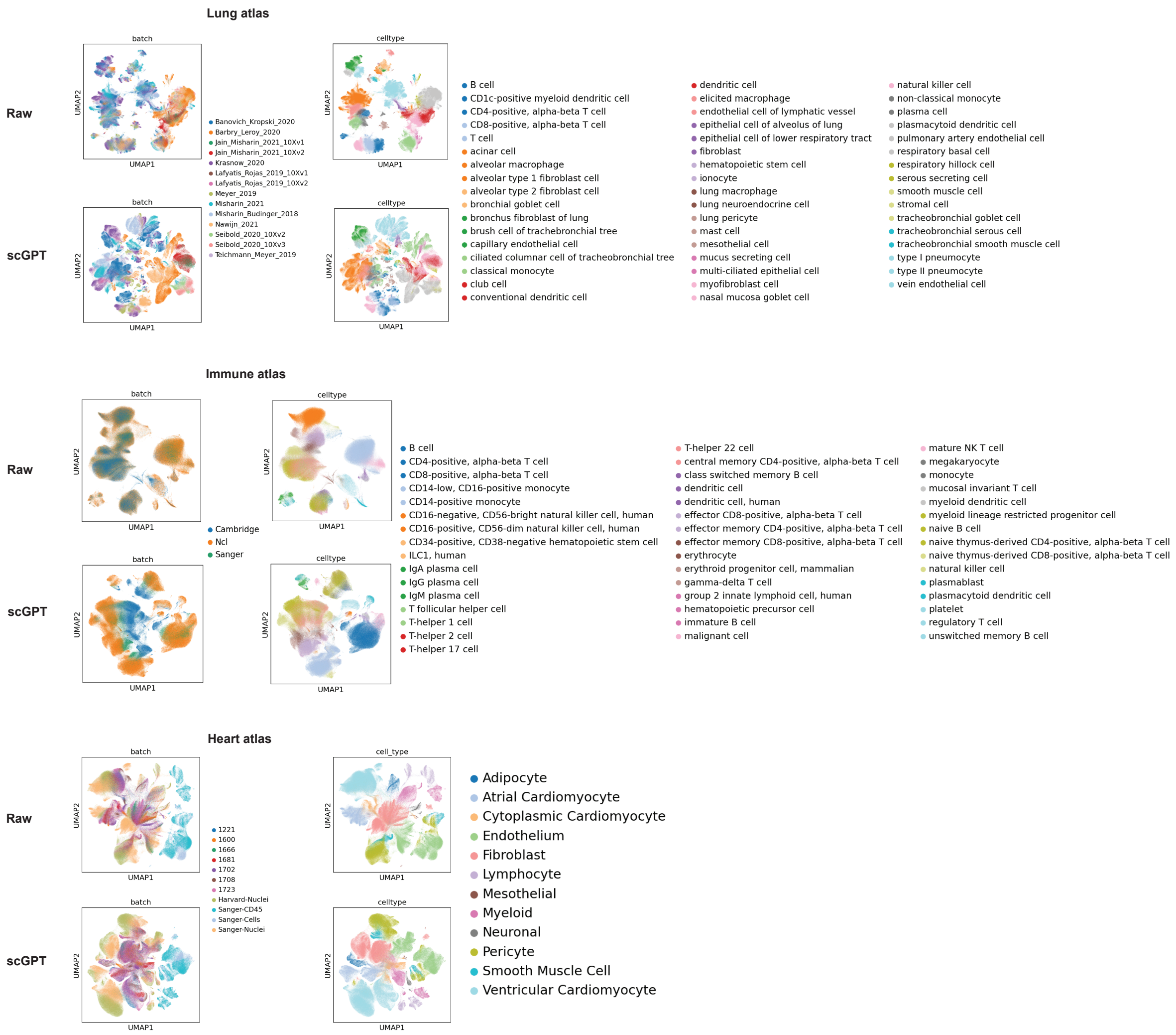

Figure S 9: UMAPs for raw data and embeddings of scGPT after batch effect correction.

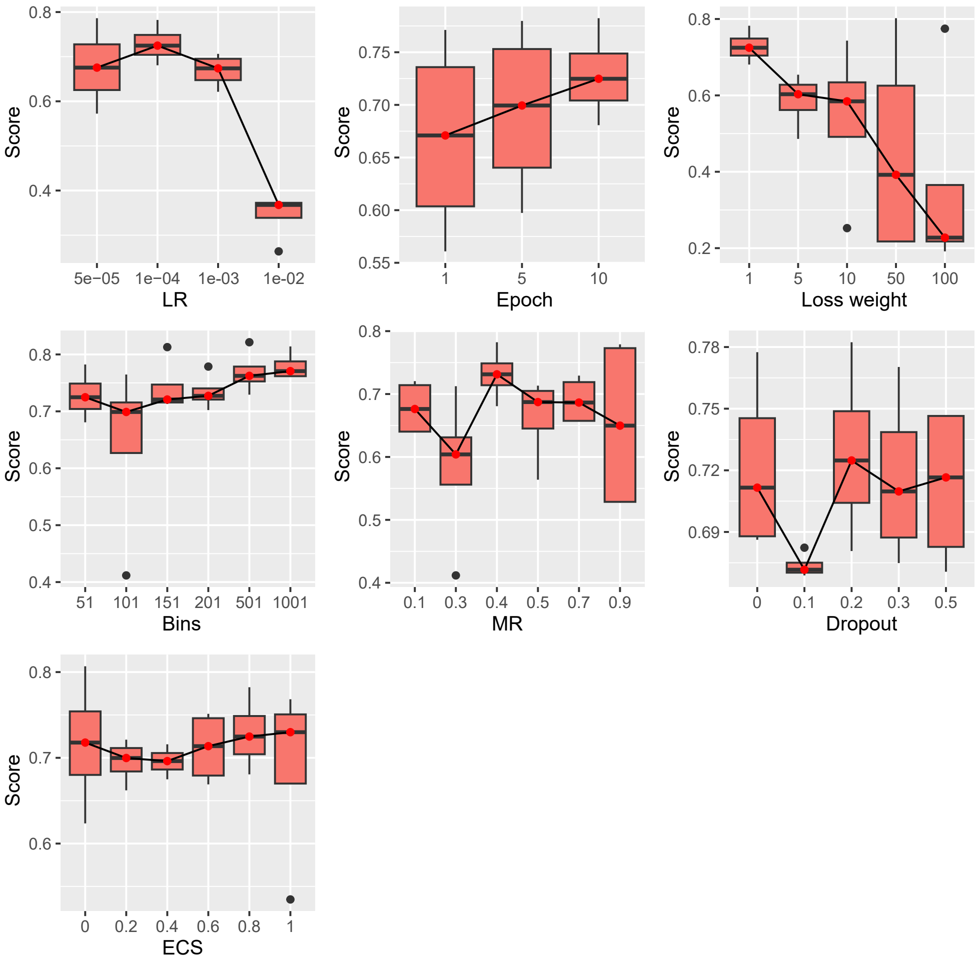

Figure S 10: Benchmarking results for different hyper-parameters for batch effect correction (one-sided Wilcoxon Rank-sum Test, $p - value = 0.03, n = 11$).

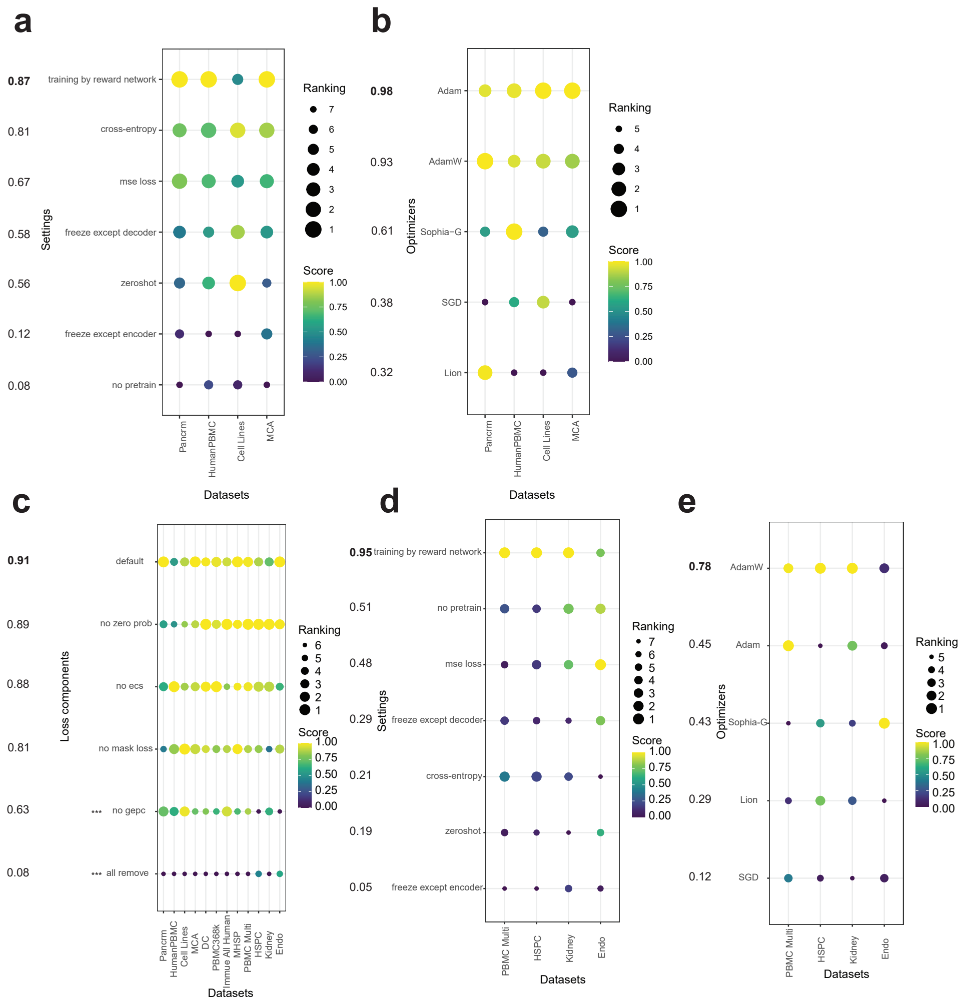

Figure S 11: Overall evaluation of different components of scGPT. (a): The final score of different settings across different datasets for batch effect correction. (b): The final score of different optimizers across different datasets for batch effect correction. (c): The final score of including different loss function terms across different datasets. The number of stars represents the significance level (one sided Wilcoxon Rank-Sum test, sample size $n = 12$, $*** : p - value < 0.005, ** : p - value < 0.05, * : p - value < 0.1$). The numbers on the left side of each sub-figure represent the average score across different datasets for one condition. We combine the datasets for batch effect correction and multi-omic data integration for testing. (d): The final score of different settings across different datasets for multi-omic data integration. (e): The final score of different optimizers across different datasets for multi-omic data integration.

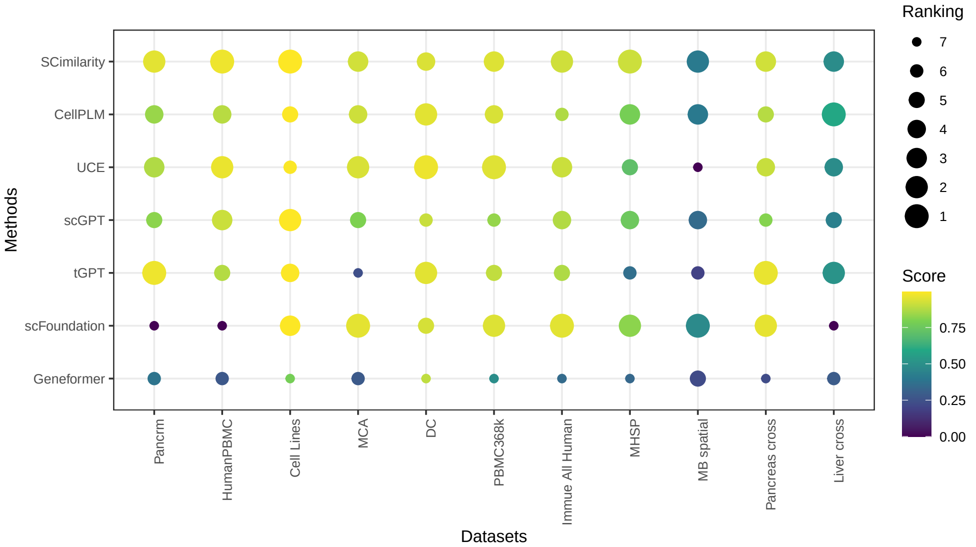

Figure S 12: Accuracy of different scFMs (zero-shot performances) for cell-type annotation.

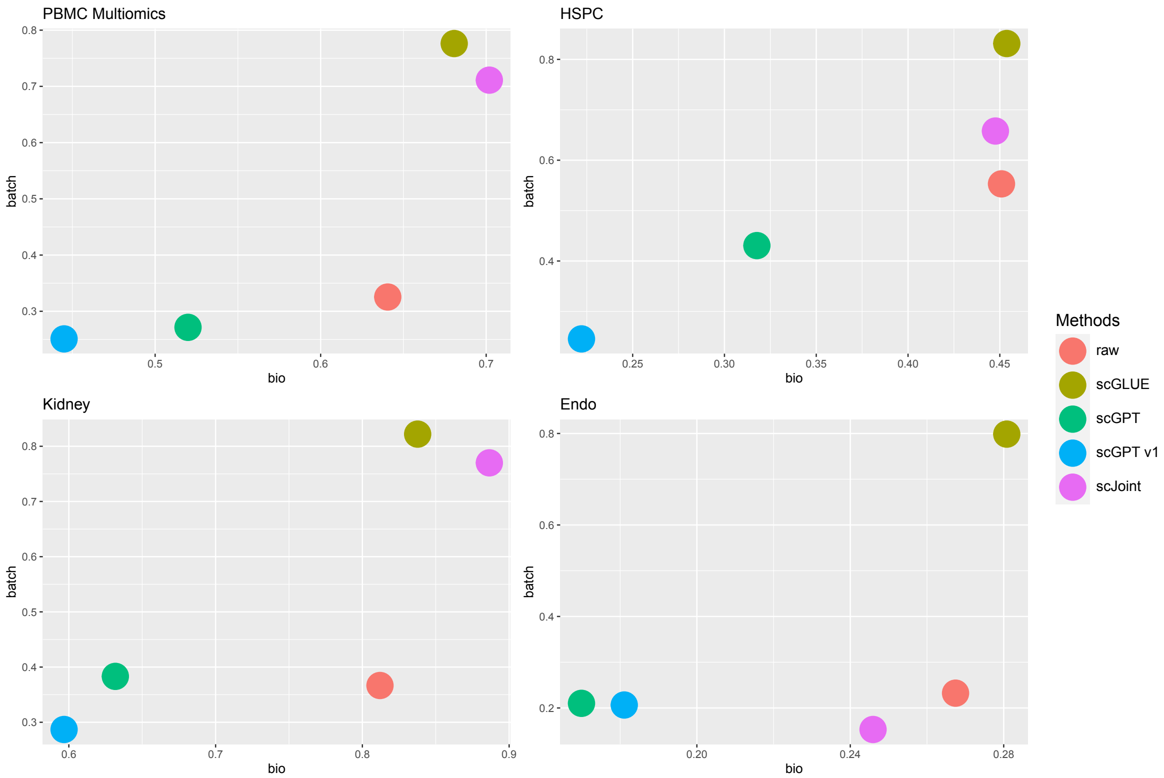

Figure S 13: Visualization of batch effect removal score and biology variation conservation score in the evaluation of multi-omic data integration task for each dataset.

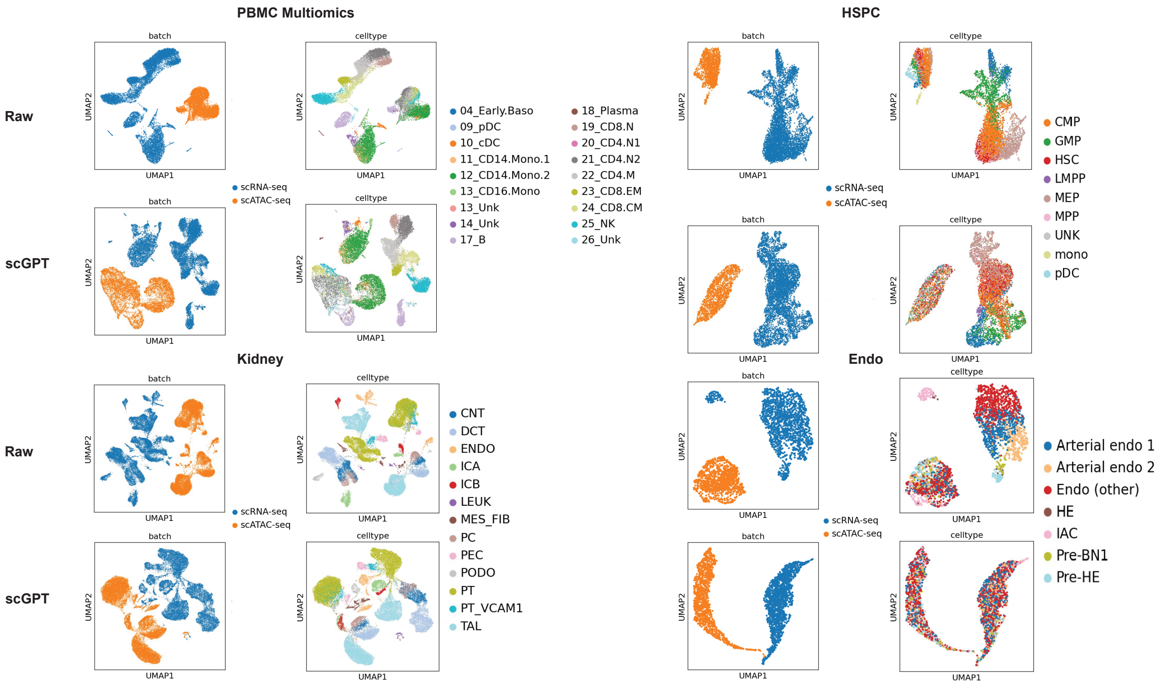

Figure S 14: UMAPs for raw data and embeddings of scGPT after multi-omic data integration.

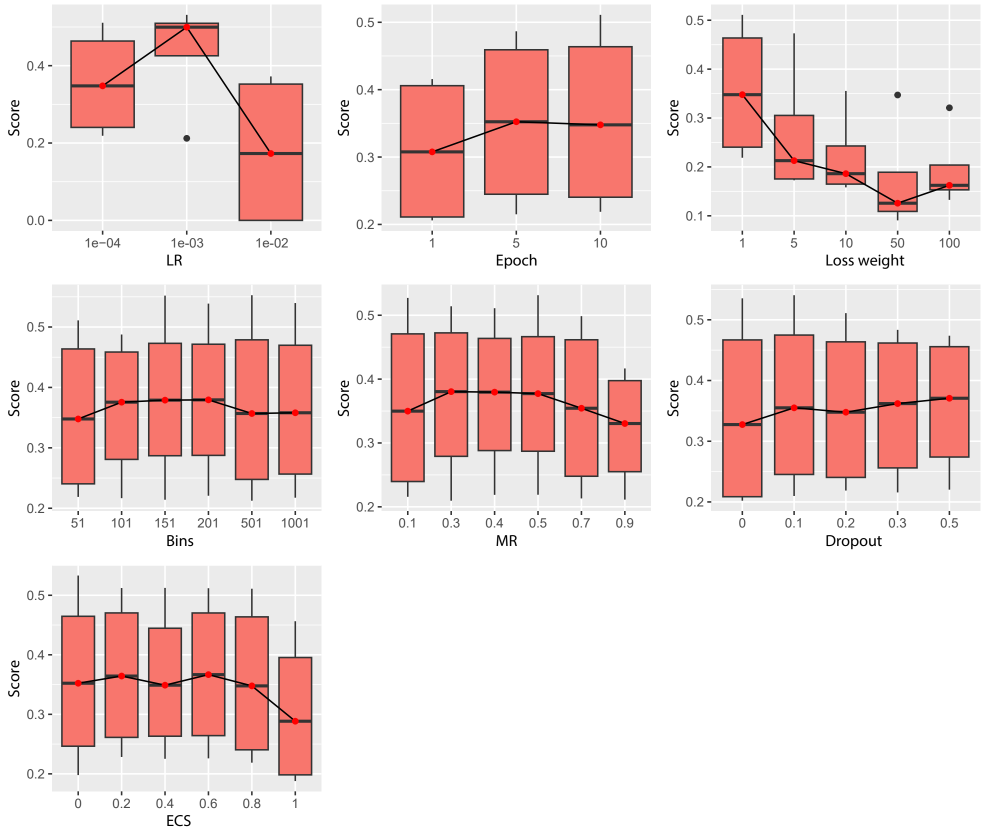

Figure S 15: Tuning parameters for multi-omic data integration. Sub-figures represent the score of scGPT under different hyper-parameters after training (sample size $n = 4$).

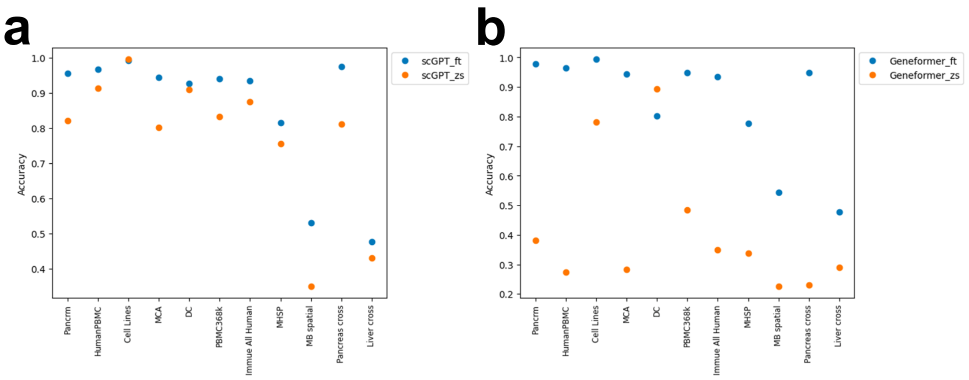

Figure S 16: Results of cell-type annotation with the different modes of scGPT and Geneformer. (a) The performance of cell-type annotation of scGPT based on both fine-tuning mode and zero-shot learning mode. (b) The performance of cell-type annotation of Geneformer based on both fine-tuning mode and zero-shot learning mode.

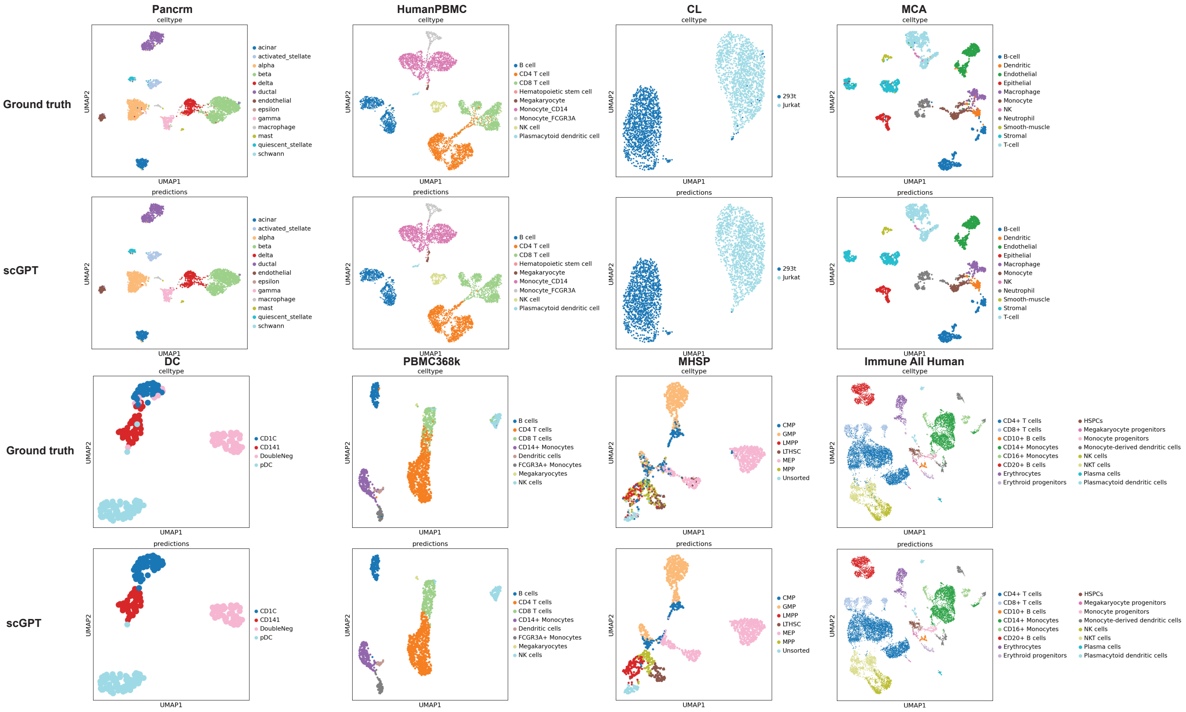

Figure S 17: UMAPs for ground truth cell types and prediction results based on scGPT.

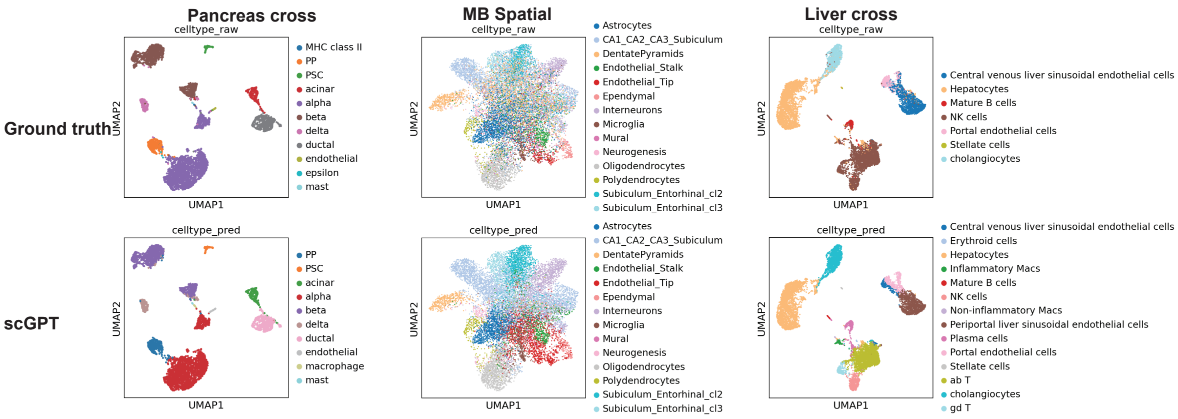

Figure S 18: UMAPs for ground truth cell types and prediction results based on scGPT.

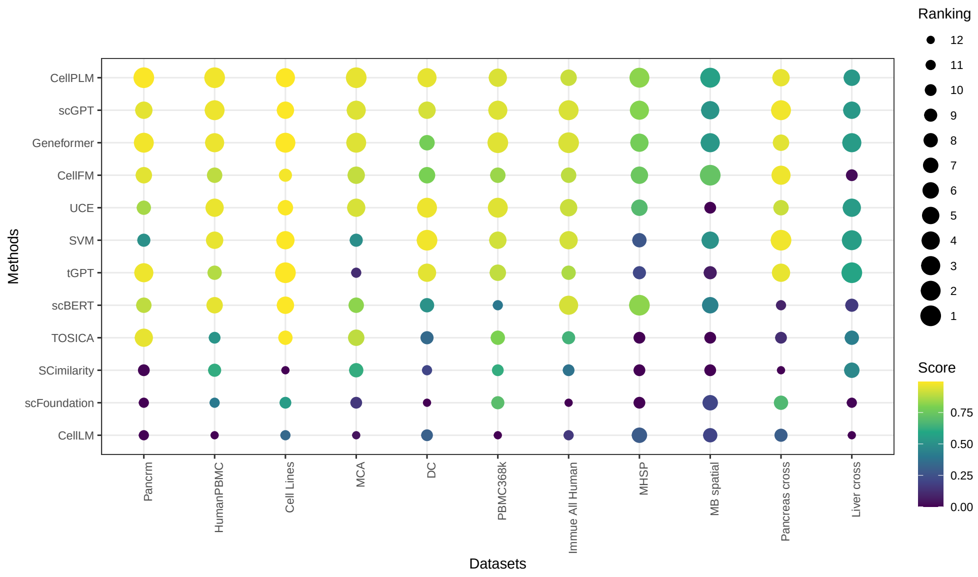

Figure S 19: Weighted F1 score across different methods for cell-type annotation.

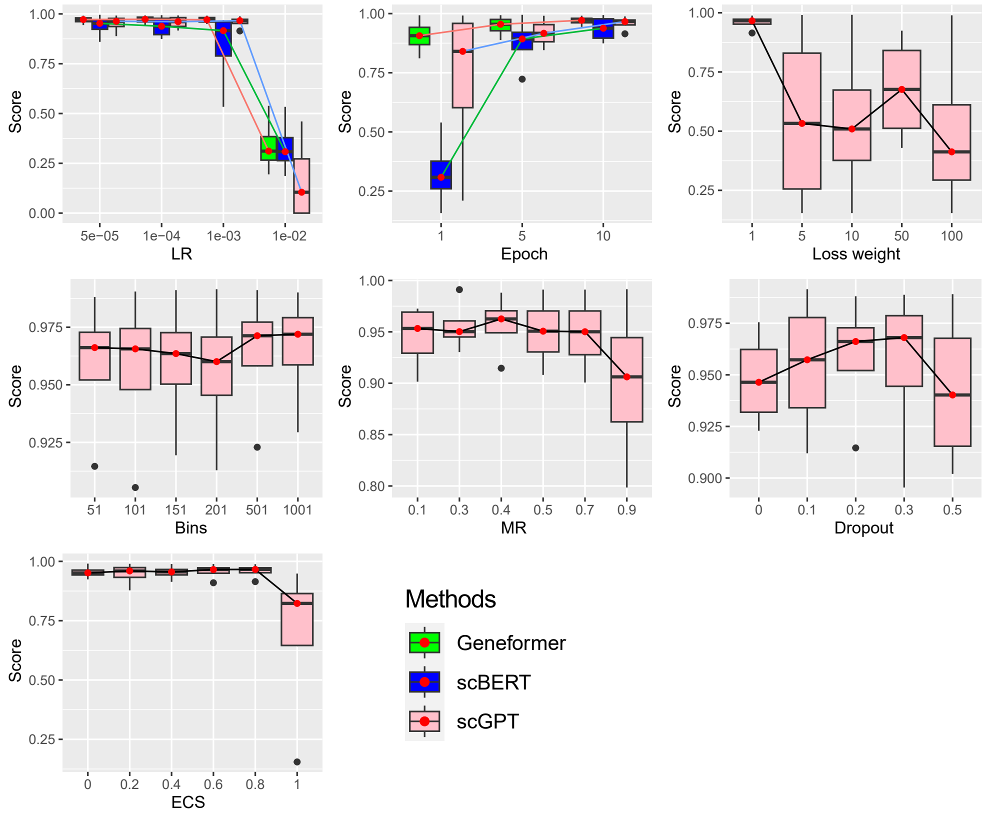

Figure S 20: Tuning parameters for cell-type annotation. Sub-figures represent the score of scGPT under different hyper-parameters after training (sample size $n = 4$).

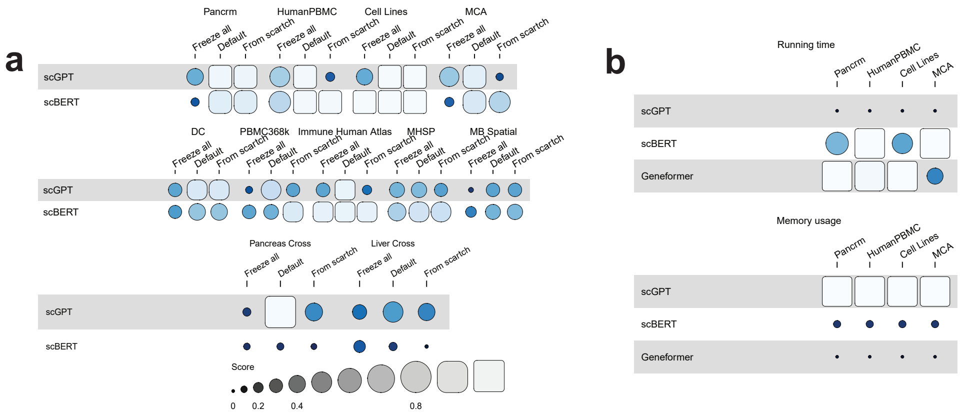

Figure S 21: Results of different settings, running time, and memory usage for cell-type annotation task. (a): Accuracy of scGPT and scBERT for the Cell-type Annotation task across different datasets. (b): Scaled running time (up) and scaled memory usage (down) statistics for all three single-cell FMs.

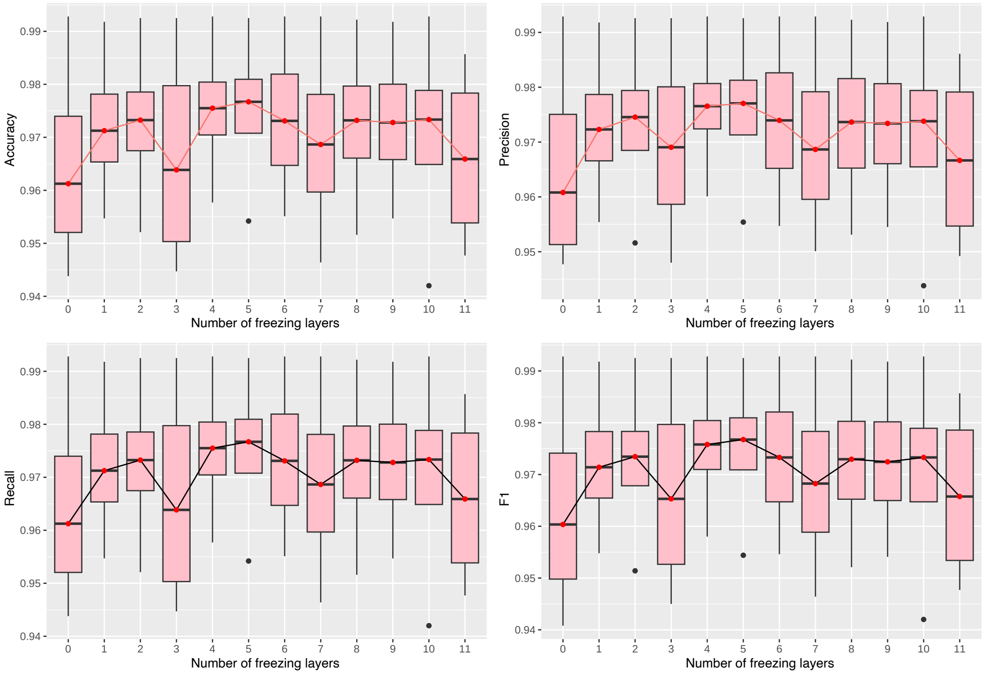

Figure S 22: Results of cell-type annotation with the different number of freezing layers based on scGPT. The x-axis represents the number of freezing layers and the y-axis represents the value of specific metrics (sample size $n = 4$).

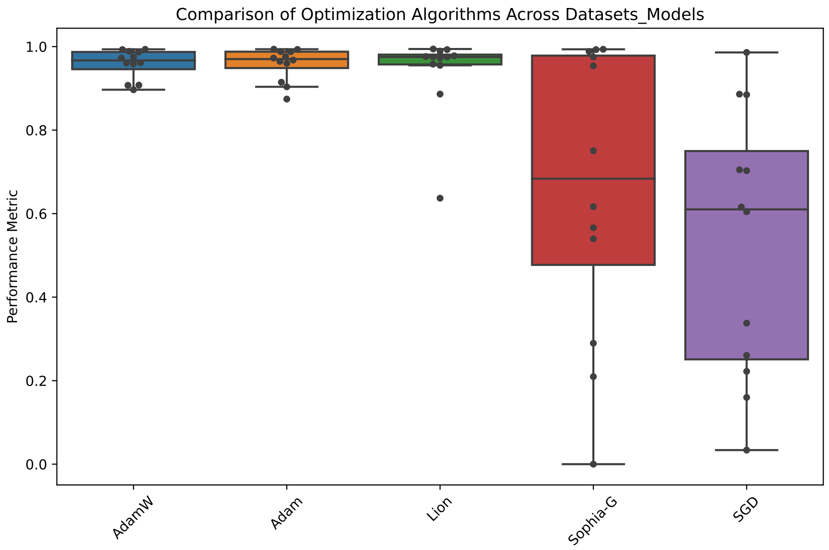

Figure S 23: Benchmarking results for different optimizers for cell-type annotation. Here each box contains the scores of fine-tuned scFMs across different datasets (sample size $n = 10$).

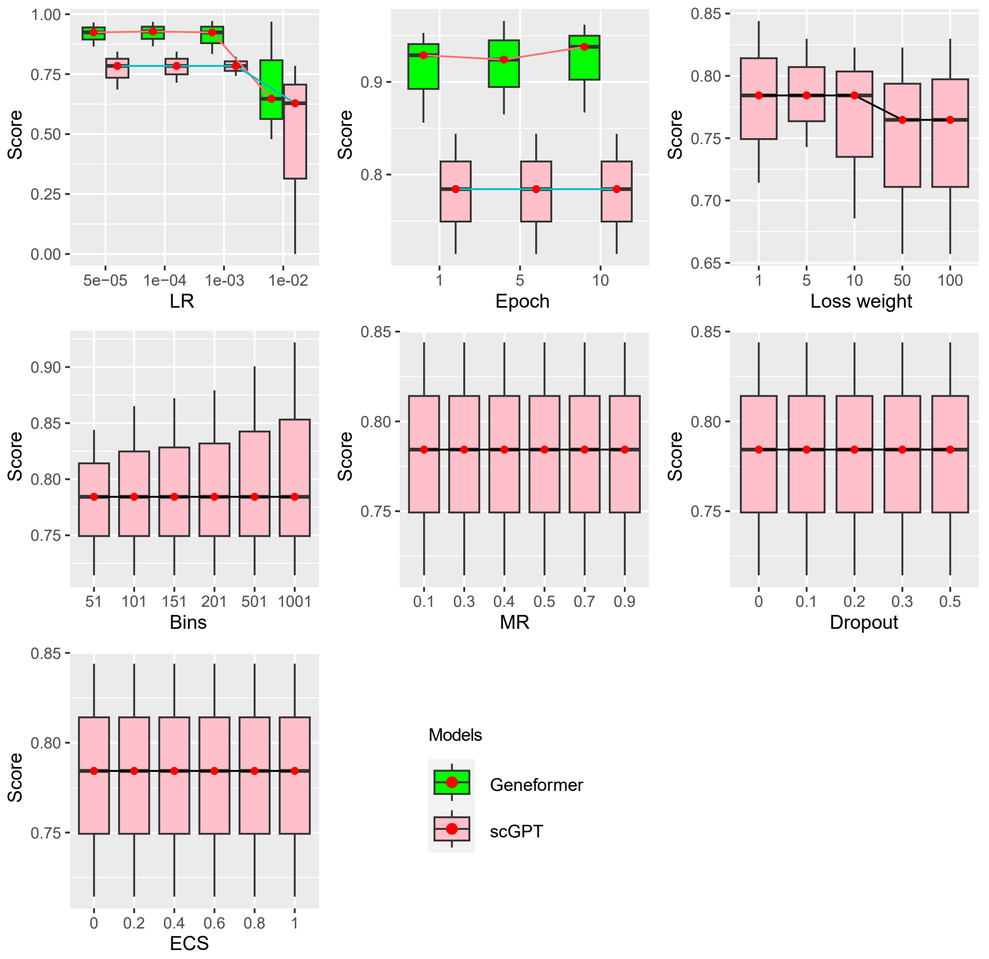

Figure S 24: Tuning hyper-parameters for gene function prediction. Sub-figures represent the score of scGPT under different hyper-parameters after training (sample size $n = 3$).

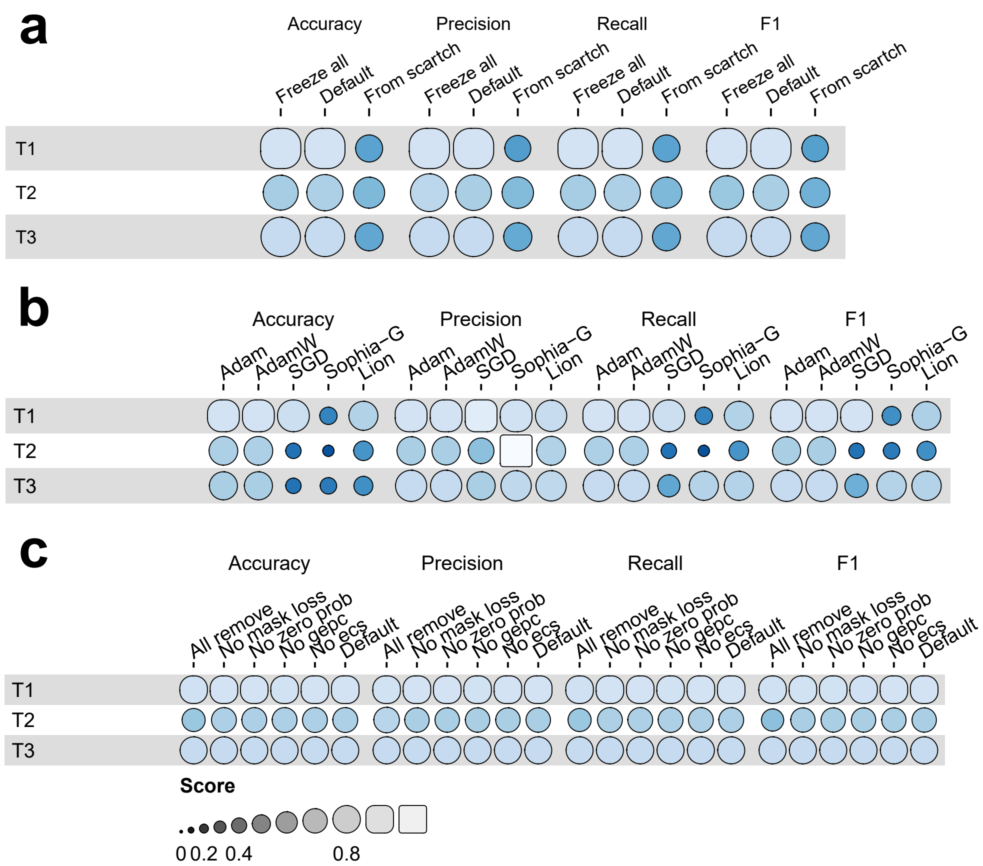

Figure S 25: Benchmarking results of different initial settings, optimizers, and loss components for gene function prediction. T1-T3 represent different gene prediction cases. (a): Results of adjusting initial settings. (b): Results of adjusting optimizers. (c): Ablation tests based on different loss components.

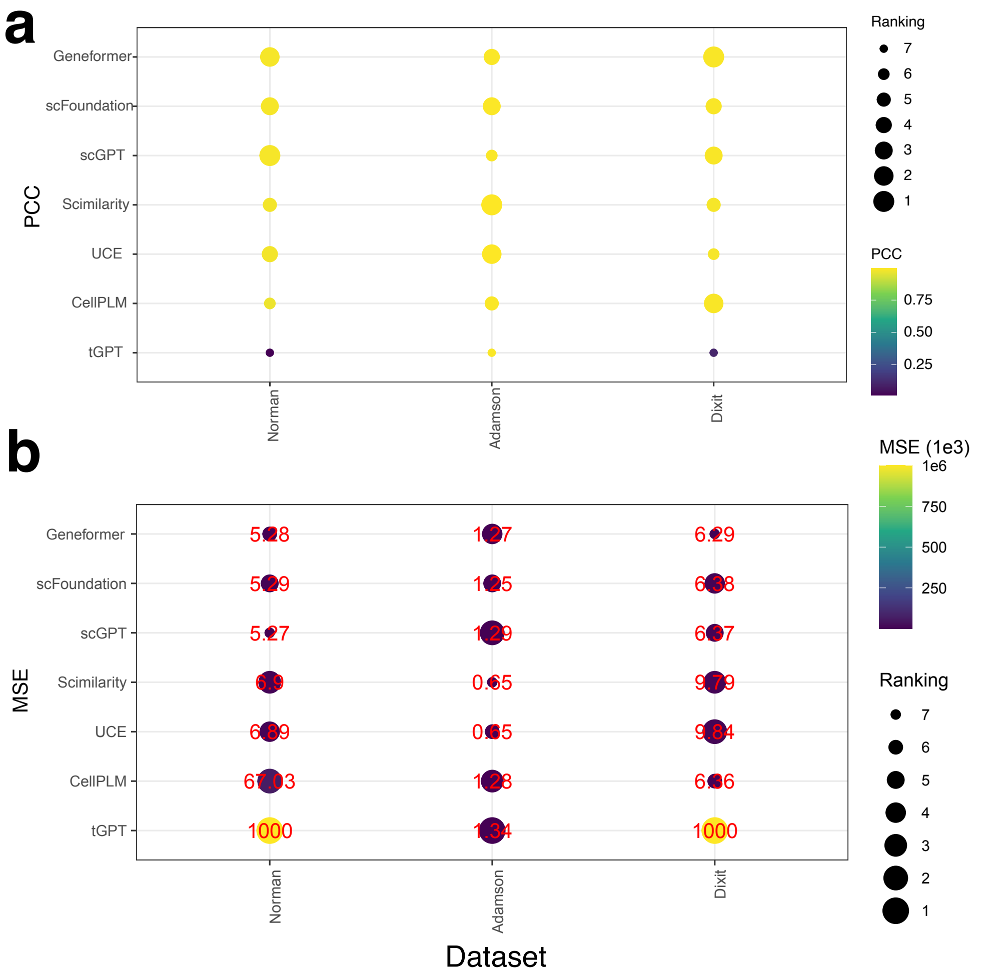

Figure S 26: Comparison based on the zero-shot settings of different scFMs. (a) PCC based on the results from different scFMs across three datasets. (b) MSE based on the results from different scFMs across three datasets.

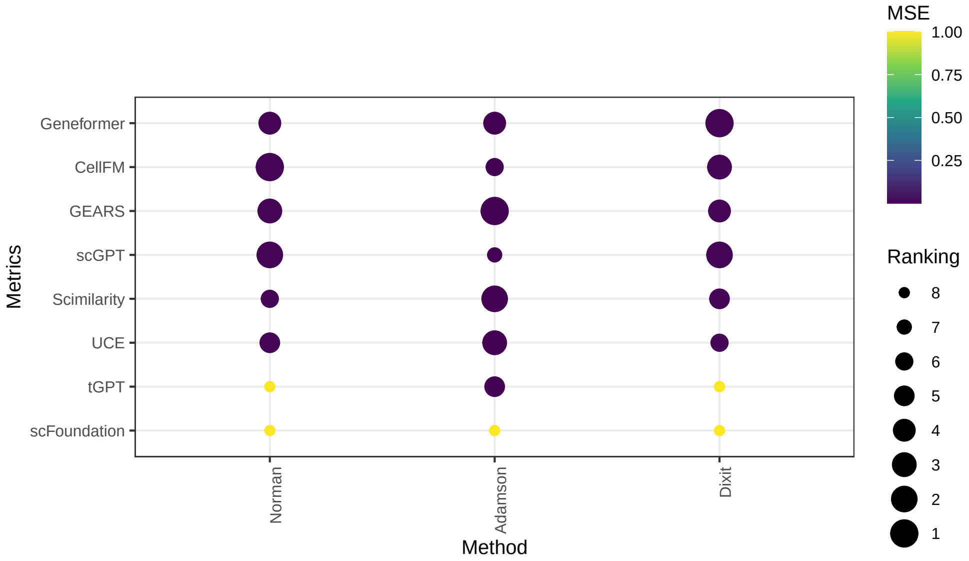

Figure S 27: MSE across different models to evaluate the performances for perturbation prediction. The maximal value for display is 1 for better visualization, while the true MSE of tGPT is larger than 1e5 for both Norman dataset and Dixit dataset. scFoundation meets the OOT error.

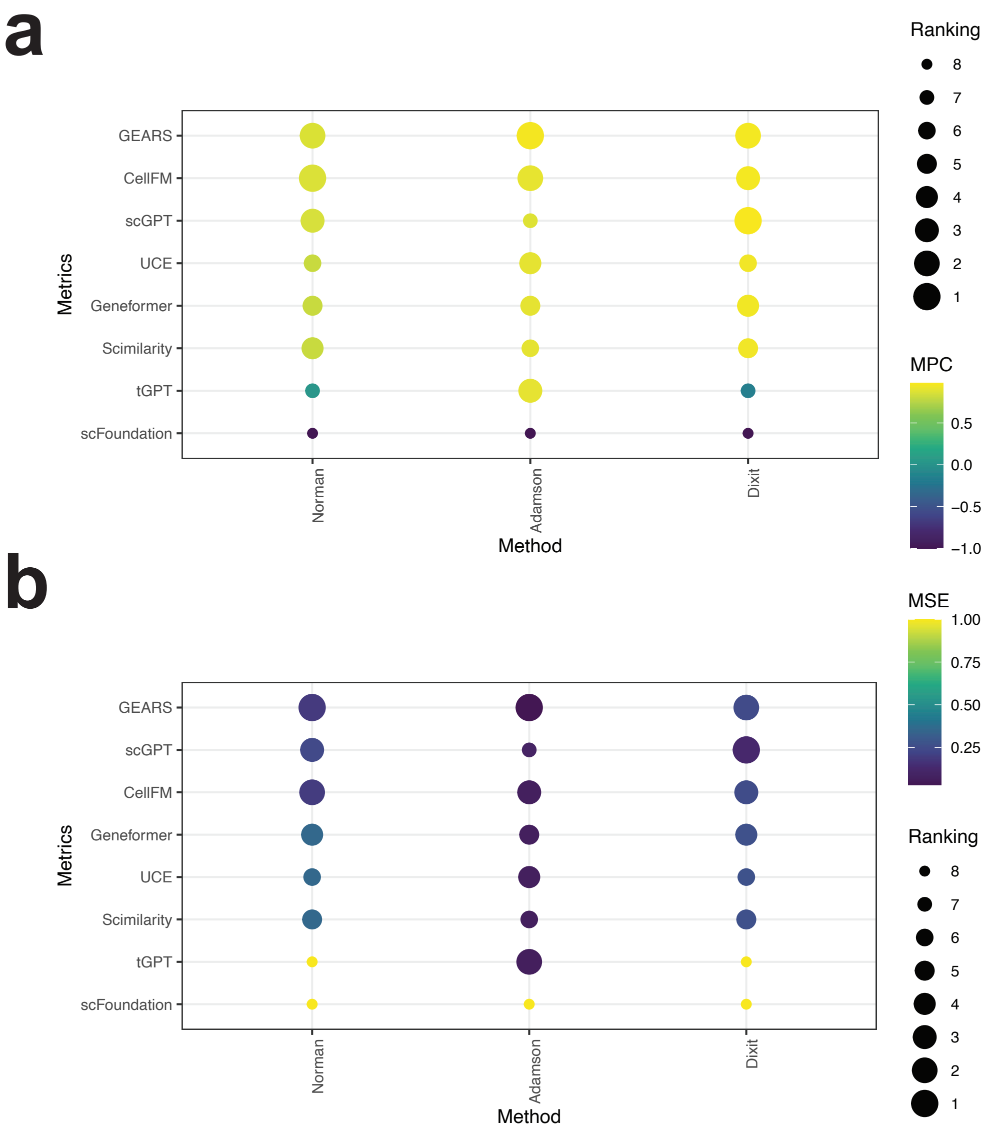

Figure S 28: Analysis of models for perturbation prediction based on DEGs. (a) MPC across different models to evaluate the performances for perturbation prediction. The averaged scores are reported in the figure. (b) MSE across different models to evaluate the performances for perturbation prediction.

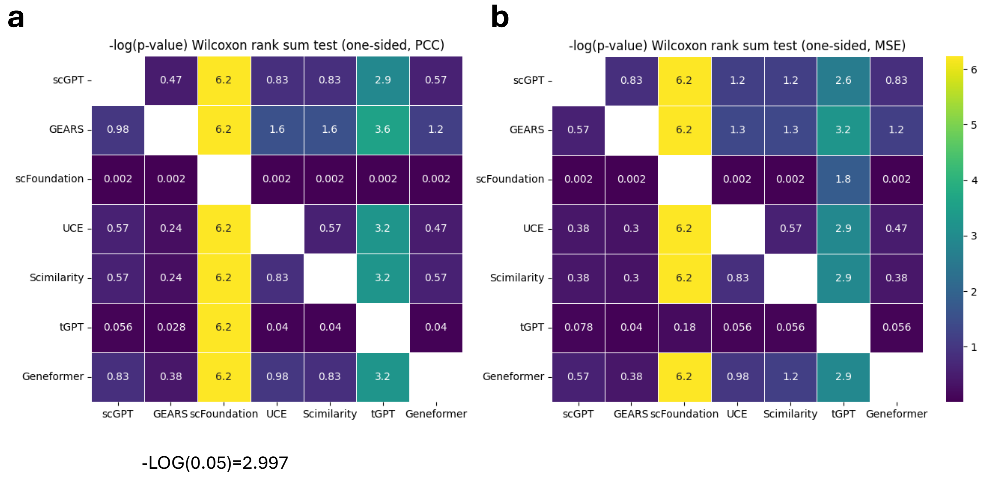

Figure S 29: Heatmap of $-log(p-value)$ to support the statistical analyses results of the perturbation prediction task. We conducted one-sided Wilcoxon Rank sums test based on different metrics, including (a) PCC (including both all genes and DEGs) and (b) MSE (including both all genes and DEGs), to examine the comparisons of different models. We mark the significance threshold 0.05 and corresponding $-log(p-value)$.

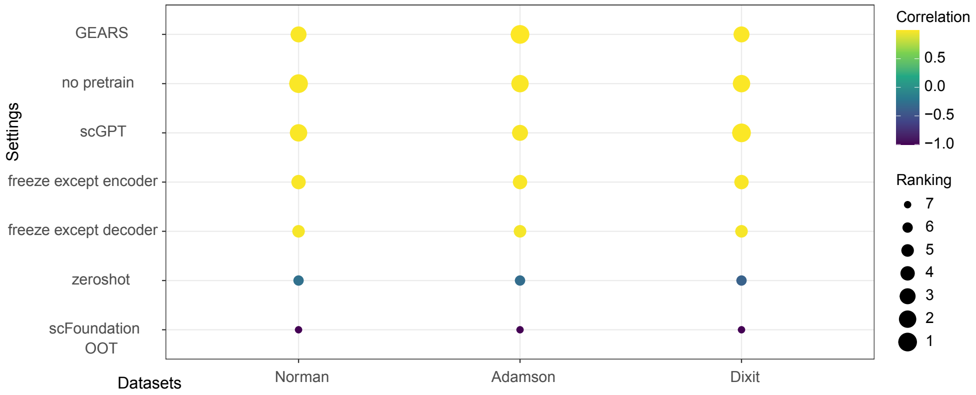

Figure S 30: MPC across different fine-tuned-based models and settings of scGPT to evaluate the performances for perturbation prediction.

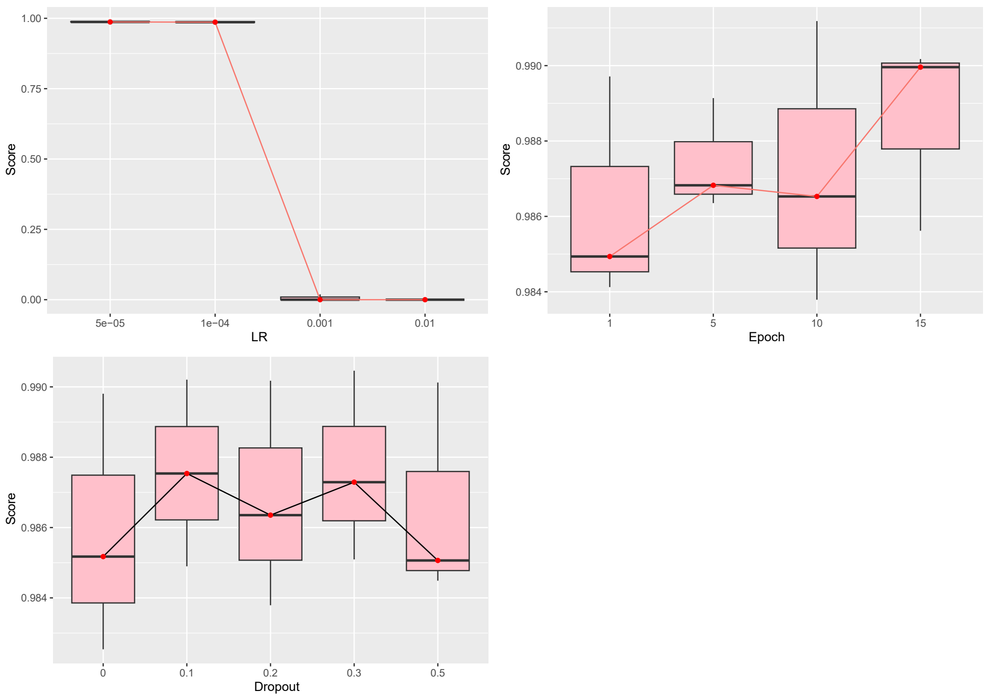

Figure S 31: Tuning hyper-parameters for perturbation prediction. Sub-figures represent the score of scGPT under different hyper-parameters after training (sample size $n = 3$).

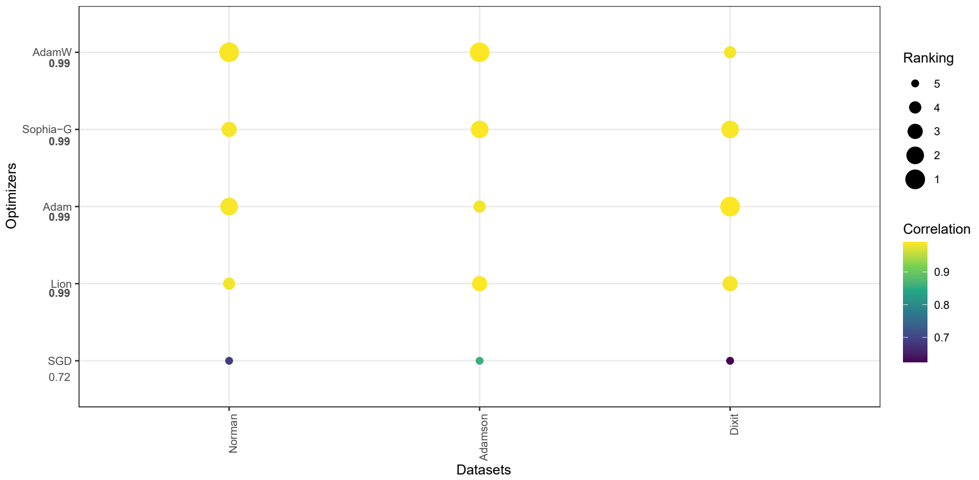

Figure S 32: Benchmarking results of different optimizers for perturbation prediction.

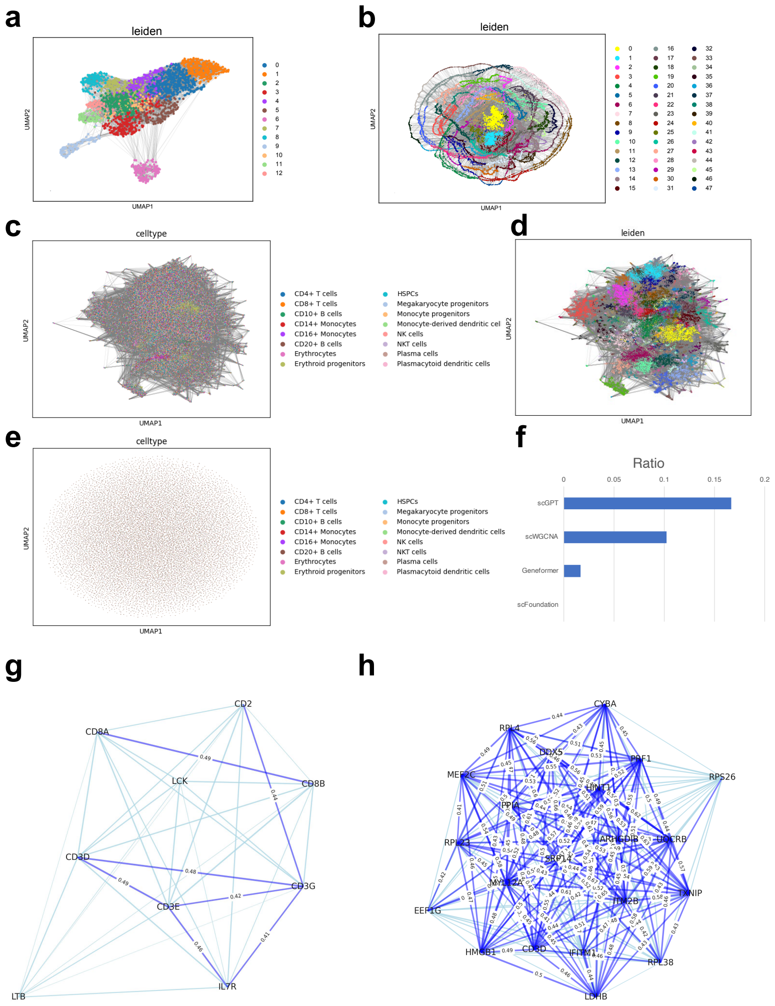

Figure S 33: Examples of GCN inference results for the Immune Human Atlas (IHA) dataset. (a): Dataset-level gene embeddings from scGPT colored by Leiden clusters. (b): Dataset-level gene embeddings from Geneformer colored by Leiden clusters. (c): Cell-type-level gene embeddings from scGPT colored by the cell types. (d): Leiden cluster results based on the cell-type-level gene embeddings from scGPT. (e): Cell-type-level gene embeddings from Geneformer colored by the cell types. We omitted plotting the Leiden cluster results of Geneformer because of the plotting size limit. (f): Comparison of significant pathways ratio between scGPT and Geneformer for CD3-related gene sets. (g): An example of GCN for IHA based on scGPT. It is a network with CD3-related genes as major nodes. (h): An example of GCN for the IHA dataset based on Geneformer.

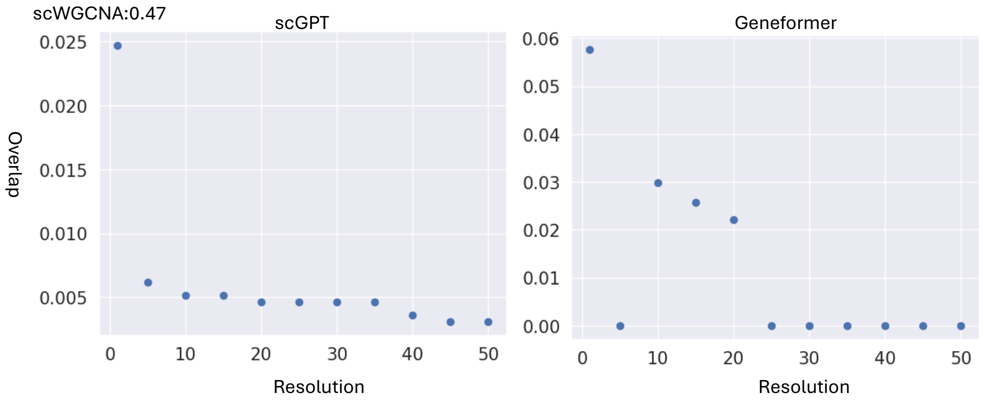

Figure S 34: Results of overlap ratio by adjusting resolution based on the gene embeddings from (a) scGPT and (b) Geneformer.

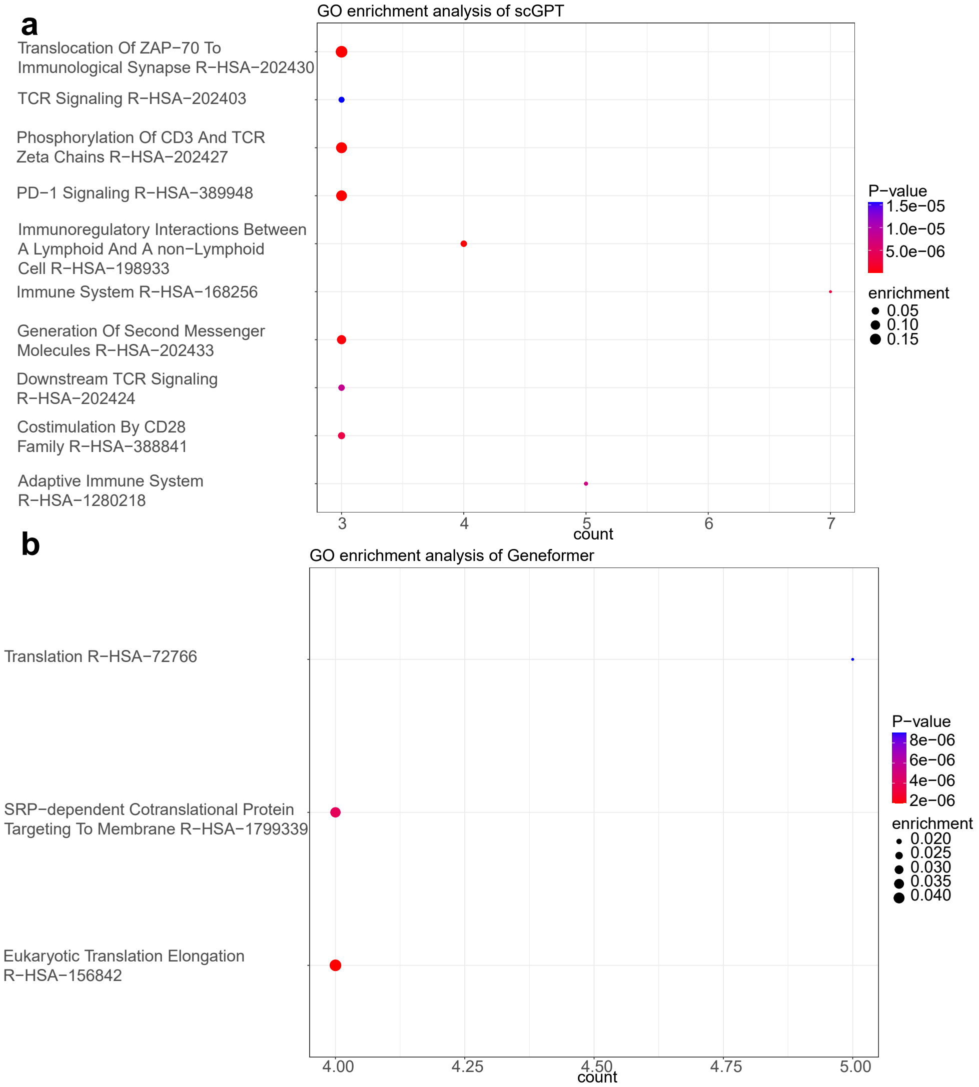

Figure S 35: Detailed pathway information. (a) The pathway enrichment information from scGPT. (b) The pathway enrichment information from Geneformer.

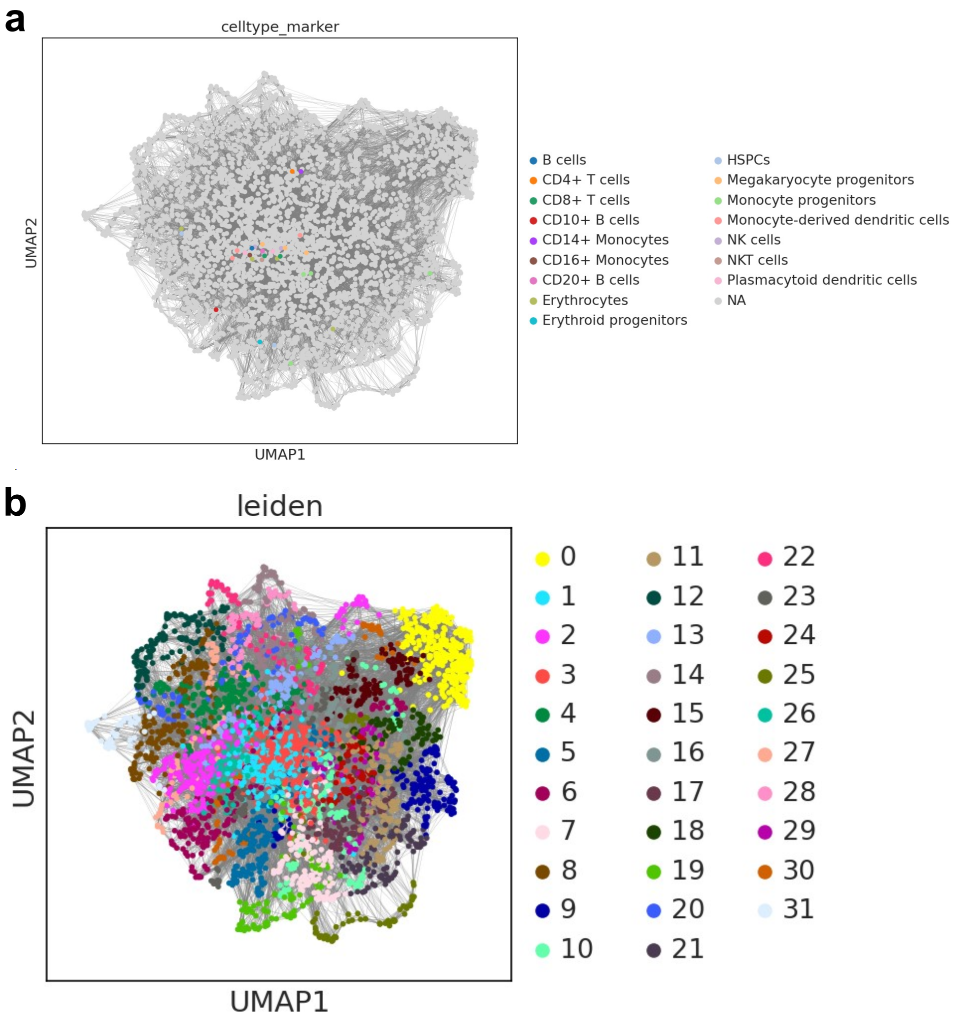

Figure S 36: Examples of dataset-level GCNs from Geneformer based on HVGs. (a) Dataset-level gene embeddings from Geneformer colored by sources of marker genes. (b) Dataset-level gene embeddings from Geneformer colored by Leiden clusters.

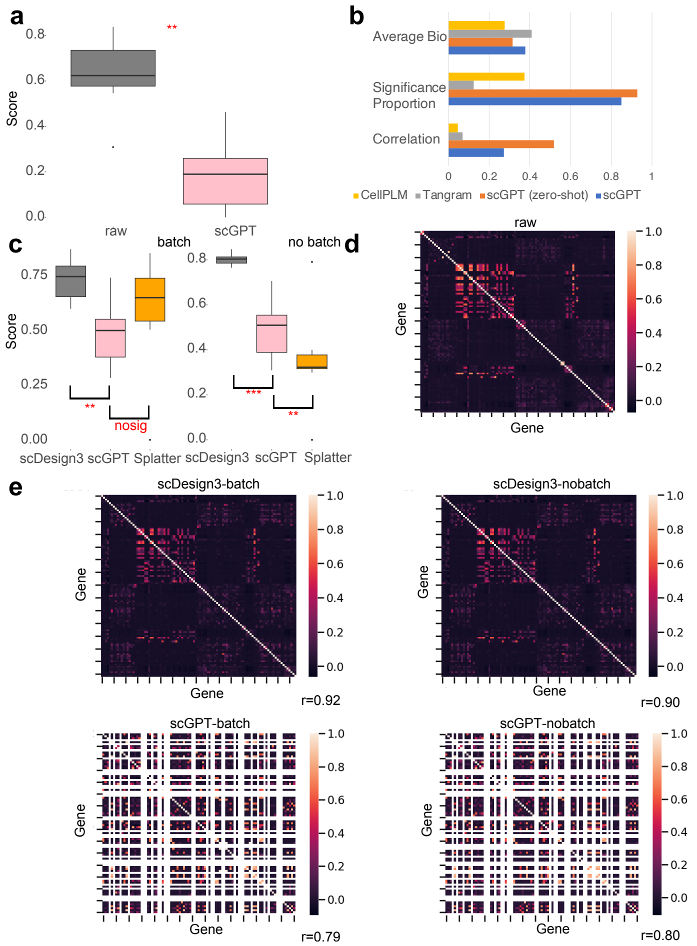

Figure S 37: Experimental results of the Imputation task and the Simulation task. The number of stars represents the significance level (one-sided Wilcoxon Rank-sum test, sample size $n = 8$, $*** : p - value < 0.005, ** : p - value < 0.05$). (a): Comparison of the average bio score between the raw data and imputed data by scGPT in the scRNA-seq imputation task. (b): Comparison of the average bio score, average correlation score, and average significance level score among Tangram, CellPLM, scGPT, and scGPT (zero-shots) in spatial transcriptomics imputation task. (c): Comparison of the average bio score among scDesign3, Splatter, and scGPT for simulation. (d): Gene-gene correlation heatmap from the raw HumanPBMC dataset. We select the subset of the top 100 highly variable genes. (e): Comparison of different simulation methods by correlation. The heatmap represents the top 100 highly variable genes (for raw and scDesign3) or the subset of the top 100 highly variable genes (for scGPT) based on the HumanPBMC dataset. The correlation "r" represents the Pearson correlation between the gene correlation of raw data and the gene correlation of simulation data. The white section represents NaN values caused by the problematic model outputs.

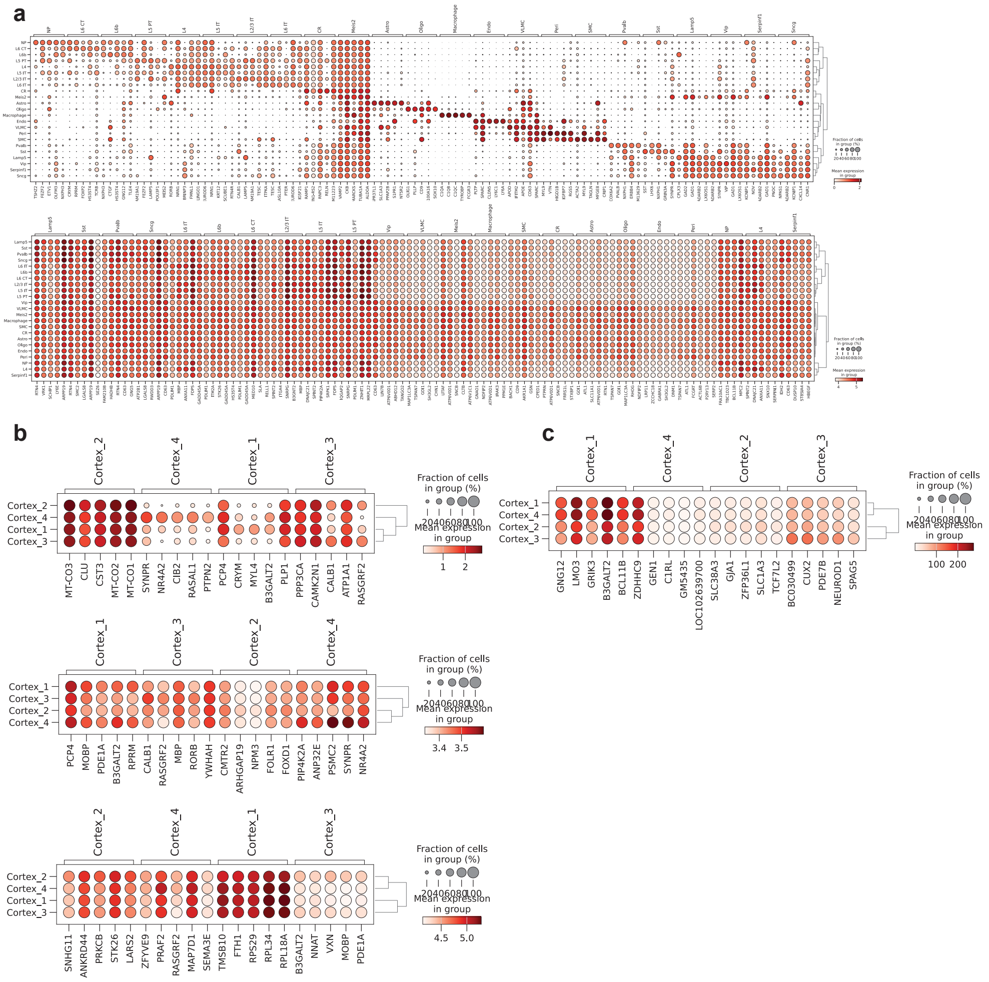

Figure S 38: Deferentially expressed gene discovery based on results before imputation and after imputation. We used the Mouse scRNA-seq and the Mouse spatial transcriptomic datasets as examples. (a): Deferentially expressed genes by cell types for scRNA-seq data based on pre-imputation data (top) and post-imputation data (bottom). (b): Deferentially expressed genes by cluster types for spatial transcriptomic data based on pre-imputation data (top), post-imputation data based on zero-shot learning (middle), and post-imputation data based on fine-tuning (bottom). (c): Deferentially expressed genes by cluster types for spatial transcriptomic data based on Tangram.

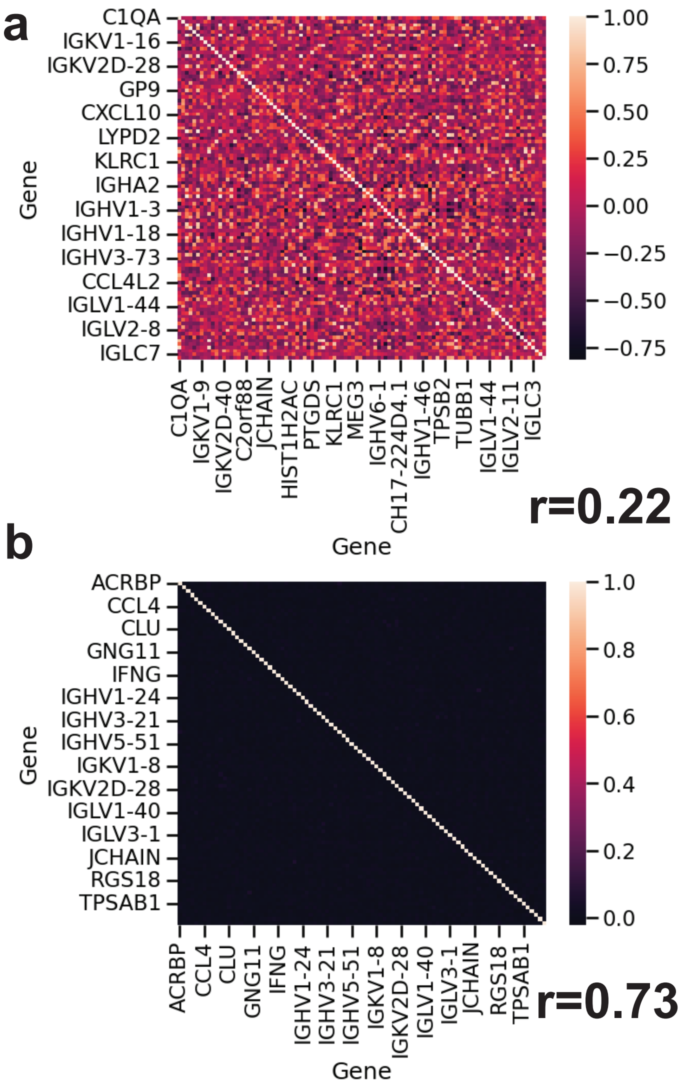

Figure S 39: Heatmaps for the gene-gene correlation of datasets simulated by Splatter, using the HumanPBMC dataset as an example. The correlation "r" represents the Pearson correlation between the gene correlation of raw data and the gene correlation of simulation data. (a): The heatmap for the simulation dataset with batch effect. (b): The heatmap for the simulation dataset without batch effect.

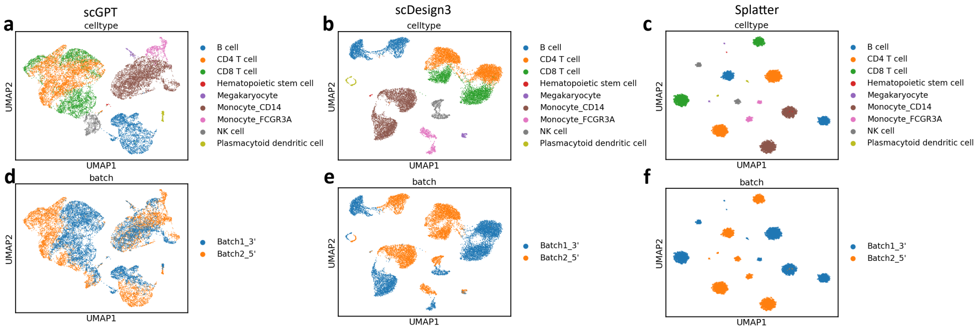

Figure S 40: UMAPs for the simulation results with batch effect, using the HumanPBMC dataset as an example.

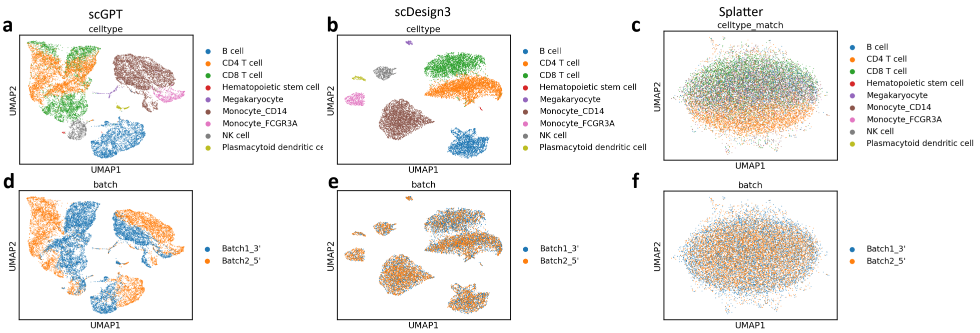

Figure S 41: UMAPs for the simulation results without batch effect, using the HumanPBMC dataset as an example.

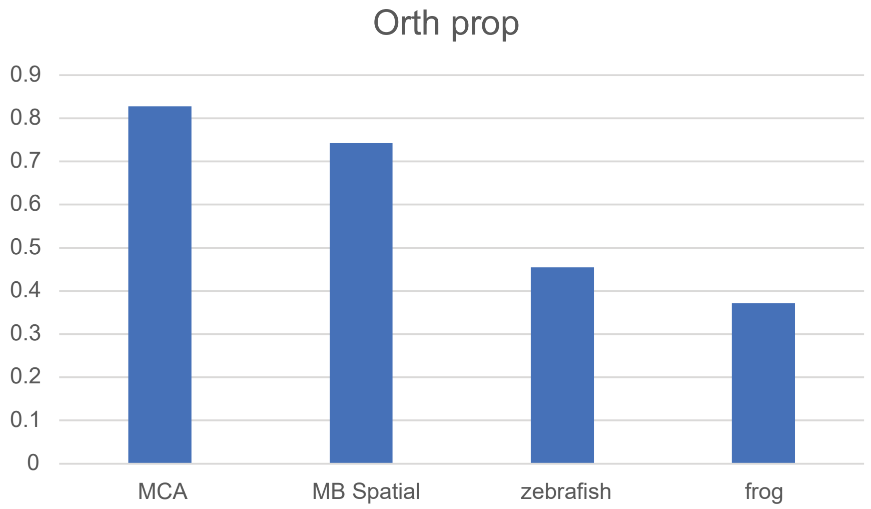

Figure S 42: Proportion of orthologous genes between specified species and human.

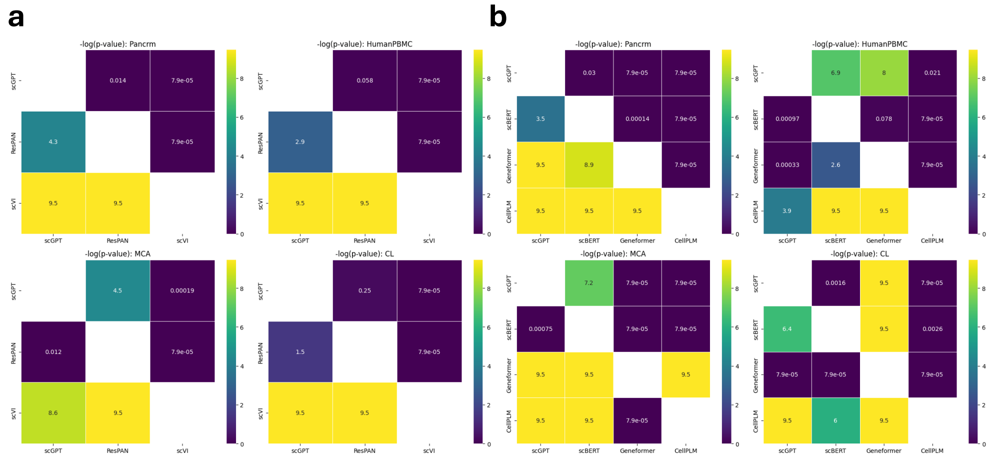

-LOG(0.05)=2.997

Figure S 43: Heatmap of $-log(p - value)$ to support the statistical analyses results of the stability examination. We conducted one-sided Wilcoxon Rank sums test based on different tasks, including (a) batch effect correction and (b) cell-type annotation, to examine the comparisons of different models. We mark the significance threshold 0.05 and corresponding $-log(p - value)$.

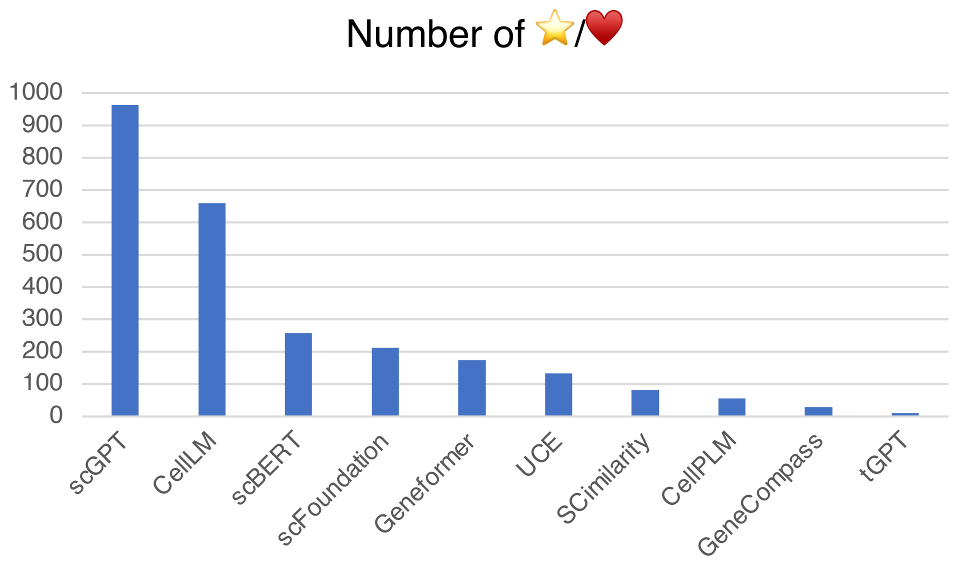

Figure S 44: The number of stars in GitHub or likes in Huggingface for different single-cell FMs.

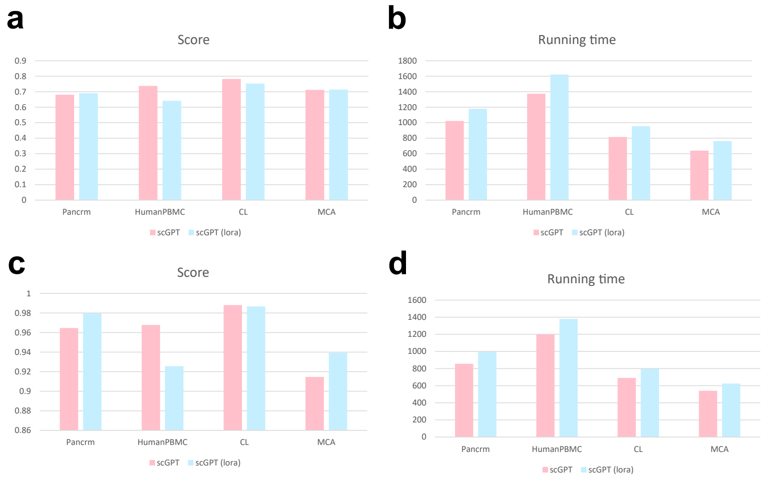

Figure S 45: Preliminary results of parameter-efficient fine-tuning based on LoRA for Batch Effect Correction (the upper two figures) and Cell-type Annotation (the bottom two figures). (a): The comparison of scores for scGPT with/without LoRA. (b): The running time for scGPT with/without LoRA. (c): The comparison of scores for scGPT with/without LoRA. (d): The running time for scGPT with/without LoRA.

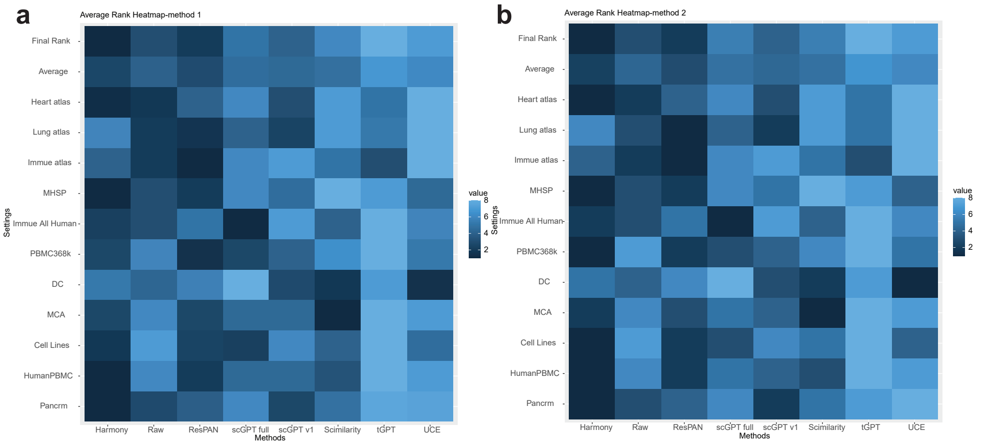

Figure S 46: Heatmaps for the ranks of different models across different datasets. (a): The heatmap of ranks based on the first method, known as adjusting the weights and computing the ranks. (b): The heatmap of ranks based on the second method, known as using the default weights for S_{bio} and S_{batch}.
